# Nuclear Factor I‐B Delays Liver Fibrosis by Inhibiting Chemokine Ligand 5 Transcription

**DOI:** 10.1002/advs.202511311

**Published:** 2025-12-23

**Authors:** Qianqian Chen, Fajuan Rui, Zhiwen Fan, Hongju Yang, Nan Geng, Chenqi Lu, Wenjing Ni, Yue Huan, Junping Shi, Chao Wu, Shengxia Yin, Wei An, Xia Lu, Qianwen Zhao, Jie Li

**Affiliations:** ^1^ Department of Infectious Diseases Nanjing Drum Tower Hospital Clinical College of Nanjing University of Chinese Medicine Nanjing Jiangsu China; ^2^ Department of Infectious Disease Nanjing Drum Tower Hospital, Affiliated Hospital of Medical School Nanjing University Nanjing Jiangsu China; ^3^ Institute of Viruses and Infectious Diseases Nanjing University Nanjing Jiangsu China; ^4^ Department of Gastroenterology The Second People's Hospital of Huai'an/The Affiliated Huai'an Hospital of Xuzhou Medical University Huai'an Jiangsu China; ^5^ Department of Pathology Nanjing Drum Tower Hospital Affiliated Hospital of Medical School Nanjing University Nanjing Jiangsu China; ^6^ Division of Geriatric Gastroenterology The First Affiliated Hospital of Kunming Medical University Kunming Yunnan China; ^7^ Jiangsu Key Laboratory of Oral Diseases Jiangsu Province Engineering Research Center of Stomatological Translational Medicine Department of General Dentistry Affiliated Hospital of Stomatology Nanjing Medical University Nanjing Jiangsu China; ^8^ Department of Infectious & Hepatology Diseases The Affiliated Hospital of Hangzhou Normal University Hangzhou Zhejiang China; ^9^ Department of Cell Biology Capital Medical University and the Municipal Key Laboratory for Liver Protection and Regulation of Regeneration Beijing China; ^10^ Department of Cardiology Shanghai Sixth People's Hospital Affiliated to Shanghai Jiao Tong University School of Medicine Shanghai China; ^11^ State Key Laboratory of Analytical Chemistry for Life Science Nanjing China

**Keywords:** Chemokine C─C motif ligand 5, hepatic stellate cells, liver fibrosis, metabolic dysfunction‐associated steatohepatitis, nuclear factor I‐B

## Abstract

Metabolic dysfunction‐associated steatohepatitis (MASH) is a significant contributor to liver fibrosis due to hepatic stellate cells (HSCs) activation. The anti‐apoptotic gene Nuclear Factor I‐B (NFIB) has been implicated in regulating cell proliferation and differentiation in the context of liver injury. However, its role in HSCs differentiation remains unclear. Our study reveals NFIB as the most markedly down‐regulated transcription factor during HSCs activation, as evidenced by single‐cell sequencing analysis of liver fibrosis. Clinical examination of liver tissues from MASH fibrosis patients corroborated diminished NFIB expression in HSCs across varying fibrosis stages. Using a murine model of liver fibrosis induced by a choline‐deficient, amino acid‐restricted high‐fat diet (CDAHFD) and carbon tetrachloride (CCl_4_) exposure, we observed reduced fibrosis levels following NFIB overexpression. Subsequent RNA sequencing elucidated the mechanism by which NFIB operates in liver fibrosis. Specifically, NFIB is found to directly interact with the promoter region of the chemokine C─C motif ligand 5 (CCL5), suppressing its expression and thereby mitigating liver fibrosis by inhibiting oxidative stress. These findings uncover a previously unrecognized role of the NFIB/CCL5 axis in liver fibrosis progression, presenting a novel therapeutic target for liver fibrosis management.

## Graphical abstract

This study identifies the transcription factor Nuclear Factor I‐B (NFIB) as a key suppressor of liver fibrosis. NFIB expression declines during hepatic stellate cell activation, and its overexpression reduces fibrosis in mice models. The mechanism involves NFIB directly repressing chemokine C─C motif ligand 5 (CCL5), thereby alleviating oxidative stress. The novel NFIB/CCL5 axis presents a promising new therapeutic target for combating progressive liver disease.

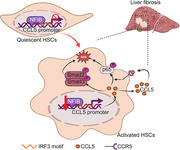

## 1 Introduction

Chronic liver injury can result in the progressive accumulation of extracellular matrix (ECM) proteins at the site of tissue damage, thereby driving the onset and progression of liver fibrosis [1]. Fibrosis is a response to injury that aims to limit tissue damage; however, prolonged fibrosis can impair the structure and function of tissue, leading to the development of cirrhosis and hepatocellular carcinoma (HCC) [2]. Metabolic dysfunction‐associated steatohepatitis (MASH), developed from metabolic dysfunction‐associated steatotic liver disease (MASLD), is a major route to induce liver fibrosis [3, 4, 5, 6]. It is reported that 20%–30% of patients with MASH will develop liver fibrosis, and of these individuals, approximately 20% will progress to cirrhosis, which severely affects the prognosis of patients [5, 6, 7]. However, there are currently no definitively effective clinical strategies to delay or treat liver fibrosis. Therefore, it is crucial to investigate the mechanisms underlying the development of liver fibrosis and identify effective therapeutic targets for its treatment.

Hepatic stellate cells (HSCs) are recognized as central drivers in the pathogenesis and advancement of liver fibrosis [8]. Under homeostasis, quiescent HSCs serve as retinol‐storing pericytes in the liver. However, under substantial chronic or acute stimulation by toxic factors, liver fibrosis is mainly mediated by the activation of liver‐resident HSCs, which produce dualplexing of collagen I/III and elastic fibers for ECM accumulation and scar formation [9]. Activated HSCs involve a series of cascades, including reactive oxygen species production, cell proliferation, and differentiation [10]. Although previous studies have explained the underlying mechanisms of liver fibrosis, the target of HSC activation is still unclear.

Chemokine C─C motif ligand 5 (CCL5) plays a crucial role in modulating inflammation and reactive oxygen species production in HSCs. Specifically, CCL5, originating from macrophages, triggers the activation of HSCs' inflammasome and pro‐fibrotic markers. The inhibition of CCL5 with a neutralizing antibody has been shown to mitigate Hepatitis C virus‐induced liver fibrosis [11]. Similarly, HSCs‐derived CCL5 can exacerbate hepatic steatosis and inflammation, promoting the progression of MASH [12]. Targeting CCL5 and its receptor C‐C chemokine receptor type 5 (CCR5) in HSCs has been demonstrated to alleviate renal fibrosis by modulating the transforming growth factor‐beta 1 (TGF‐β1)/Smad/Snail signaling pathway [13]. Therefore, focusing on CCL5 represents a promising approach to impede the activation of HSCs.

Nuclear factor I‐B (NFIB), identified as the upstream of CCL5 in this study, is a member of the NFI family, which is composed of four members: NFIA, NFIB, NFIC, and NFIX [14, 15]. These proteins are known to bind to DNA as either homodimers or heterodimers [16]. Among the nuclear factor I (NFI) proteins, NFIB is widely expressed across human tissues and often participates in various types of cancer, including small cell lung cancer [17], melanoma [18], and breast cancer [19]. Recently, a study has found that NFIB may exert an inhibitory role in pulmonary fibrosis [20]. However, the precise molecular mechanisms underlying this regulatory function remain incompletely understood. Moreover, the potential involvement of NFIB in the differentiation of HSCs into myofibroblasts, as well as its role in the pathogenesis of liver fibrosis, has yet to be elucidated [21]. In the present study, we aimed to investigate the functional role of NFIB in liver fibrosis and to systematically elucidate the molecular mechanisms by which it modulates HSC activation.

Here, we used HSCs‐specific overexpression and blocking NFIB in mice, two animal models, to explain the potential role of NFIB/CCL5 in liver fibrosis. In the present study, we sought to clarify the role of NFIB in the liver fibrosis process with the following outstanding question: 1) Is NFIB actionable for liver fibrosis intervention? 2) How does NFIB transmit the anti‐fibrosis signal to HSCs? Our data suggest that NFIB inhibits CCL5 transcription activation in HSCs, inhibiting the phosphorylation of SMAD2/3 and the accumulation of reactive oxygen species (ROS), which attenuates liver fibrosis.

## 2 Results

### 2.1 NFIB Induction in HSCs Is Downregulated by MASH and Tracks With Human Liver Fibrosis

**FIGURE 1:**
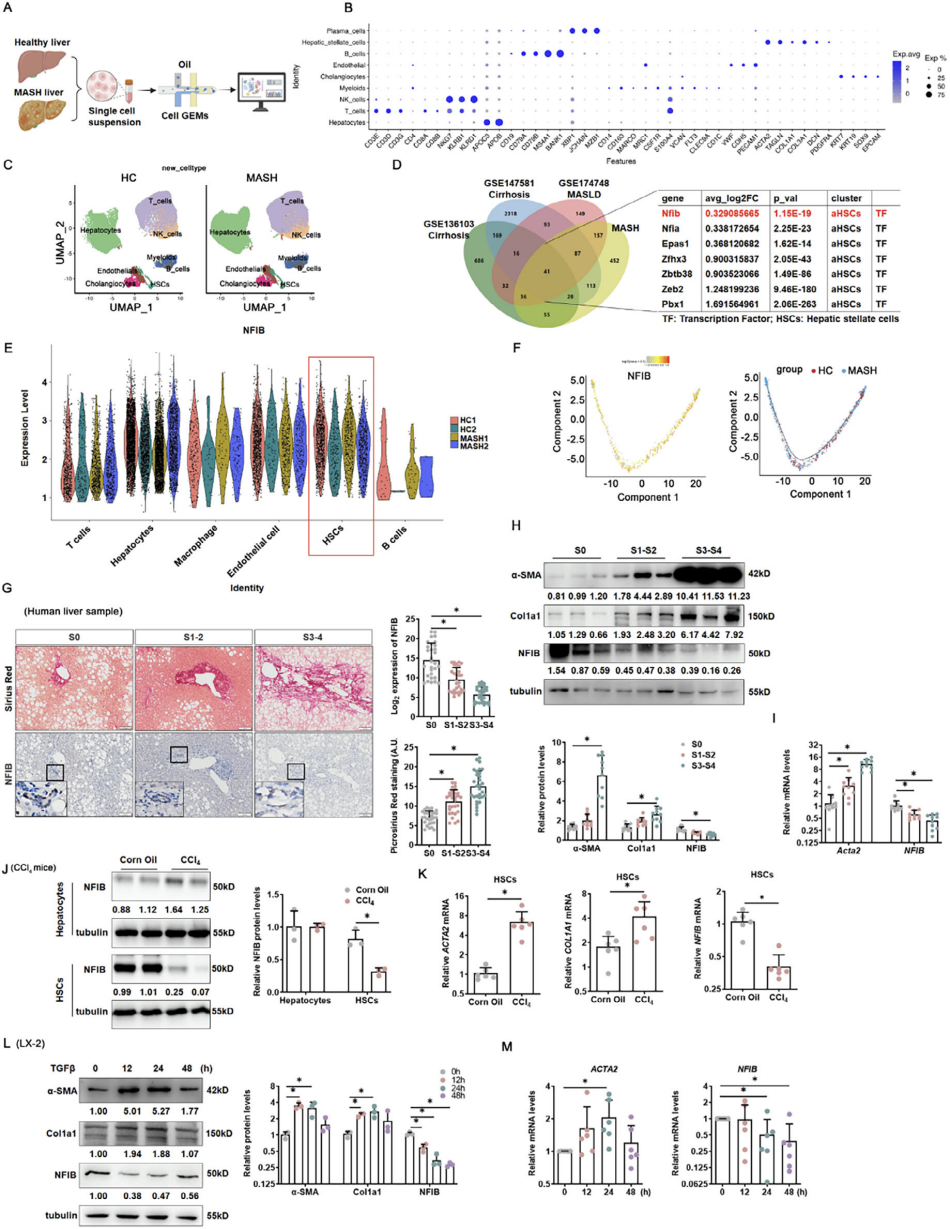
NFIB induction in HSCs is downregulated by MASH and tracks with human liver fibrosis. (A) Workflow for MASH and a healthy liver to conduct single cell sequence. (B) Dot‐plot. (C) Uniform manifold approximation and projection (UMAP) plots. (D) Wayne's analysis of single‐cell sequencing of patients with cirrhosis (GSE147581, GSE136103) and MASH (GSE174748, our own MASH single‐cell dataset). (E) NFIB expression in T cells, hepatocytes, macrophages, endothelial cells, HSCs, and B cells (our own MASH single‐cell dataset). (F) Pseudo‐time analysis of HSCs and NFIB level plotted on the trajectory. (G) Picrosirius red staining (100×) and representative NFIB immunohistochemistry(IHC) staining (100×) in human liver biopsy specimens with different fibrosis levels. *N *= 40 patients in each group. Scale bar: 50 µm. (H) Protein expression in human livers. *N *= 10 patients in each group. (I) Gene expression in human livers. *N *= 10 patients in each group. (J) Primary HSCs and hepatocytes were isolated from mice with or without carbon tetrachloride (CCl_4_)‐induced liver fibrosis. Cells were harvested after 3 weeks. Protein expression of NFIB in hepatocytes and HSCs, respectively. *N *= 3. (K) *NFIB, COL1A1*, and *ACTA2* mRNA were examined. *N *= 6. Protein (L) and gene (M) expression of NFIB and profibrotic genes in LX‐2 cells with different time courses. *N *= 3. Data are expressed as the mean±SD. *p < 0.05, two‐tailed Student's *t*‐test.

To uncover key transcription factors in HSC activation, we performed transcriptomic screening through integrated analysis of existing single‐cell sequencing datasets (GSE136103) performed with liver tissues from patients with liver cirrhosis, GSE147581 performed with liver tissues from another cohort of patients with cirrhosis, and GSE174748 performed with liver tissues derived from patients with MASLD) and the single‐cell sequence conducted by our research group. As shown in Figure 1A, human liver puncture samples (2 relative ‘Healthy’ liver vs. 2 MASH liver) were used for single‐cell sequencing. Based on canonical markers, hepatocytes, T cells, NK cells, myeloids, cholangiocytes, endothelial cells, B cells, HSCs, and plasma cells were identified (Figure 1B). We observed concordant clustering of cells based on their type‐specific gene expression patterns, where each cluster corresponded clearly to a distinct cellular identity. (Figure 1C). Because HSCs are the final effector cells of liver fibrosis [22], we focus on screening genes with significant differences in HSCs. Using 2× fold change and *p *< 0.05 as cutoff parameters, we identified 41 differentially expressed genes in the HSC population, of which 7 were transcription factors (Figure 1D). NFIB was prioritized for further investigation due to its top statistical significance among the candidates and the lack of established functional characterization in HSC activation. Indeed, our single‐cell sequence analysis showed that NFIB was only downregulated in HSCs (Figure 1E). Pseudo‐time trajectory analysis showed that NFIB was decreasingly expressed in HSCs during MASH (Figure 1F). Moreover, NFIB exhibited the same trend after re‐analysis GEO datasets (GSE136103, GSE147581, GSE174748). NFIB levels were inversely correlated with the induction of pro‐fibrotic genes (collagen type I alpha 1 [Col1a1], collagen type III alpha 1 [Col3a1]) (Figure S1A), as well as the lower level in HSCs (Figure S1B–D).

Mouse single‐cell sequence datasets were also used to identify the expression of NFIB under MASH conditions: GSE156057 and GSE192740 performed with liver tissues form MASH mice induced by western diet (WD) for 36 weeks, GSE218299 performed with liver tissues form MASH mice induced by WD for 52 weeks. Using 2x fold change and *p*<0.05 as cutoff parameters, 291 differentially expressed genes, 12 of which were transcription factors, were identified in HSCs. As shown in Figure S2A, NFIB still ranked the most significant. Besides, NFIB only reduced in the HSC population (Figure S2B–D).

To further identify the effect of NFIB in liver fibrosis, liver samples were then collected from patients with different hepatic fibrosis levels (S0: non‐fibrosis; S1‐S2: moderate fibrosis; S3‐S4: severe fibrosis). Immunohistochemical and picrosirius red staining with human tissues also revealed decreased NFIB levels with increasing fibrosis severity (Figure 1G,*N* = 40). Moreover, Western blotting and mRNA analyses showed decreased NFIB levels and increased fibrotic levels (Figure 1H,I, *N *= 10). While NFIB is also expressed in hepatocytes [21], we isolated hepatocytes and HSCs from fibrotic mice. Our findings demonstrate a significant downregulation of NFIB specifically in HSCs under fibrosis conditions (Figure 1J). Similar changes were observed in the livers of mice subjected to a CCl_4_ (Figure 1K) or bile duct ligation (BDL) challenge (Figure S3). LX‐2 cells (a cell line derived from human HSCs) treatment with TGF‐β also exhibited reduced NFIB and hyperexpression of pro‐fibrotic molecules during HSC activation (Figure 1L,M). Indeed, the spontaneous activation of HSC is accompanied by a decrease in NFIB expression (Figure S4). These results indicated that NFIB and MASH‐related liver fibrosis were inversely regulated in HSCs.

### 2.2 NFIB Induction Attenuates HSC Activation via Inhibiting Oxidative Stress

**FIGURE 2:**
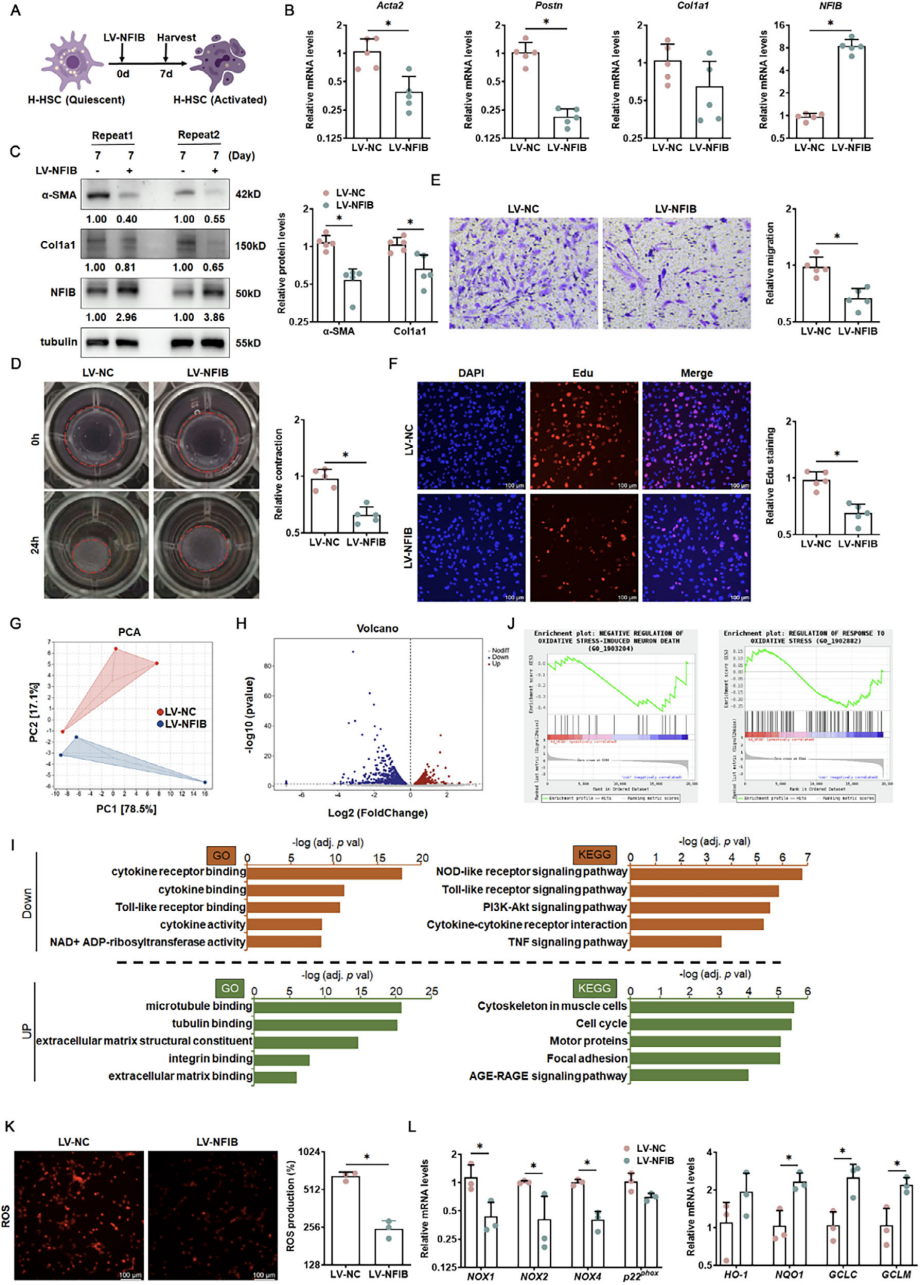
NFIB induction attenuates HSC activation via inhibiting oxidative stress. Primary human HSCs are allowed to undergo spontaneous activation with NFIB overexpression (lentivirus containing the NFIB [LV‐NFIB]) or control virus (LV‐NC). The cells were harvested on the seventh day for testing and RNA sequencing. (A) Schematic of the cell protocol in vitro. Myofibroblast marker genes were examined using quantitative PCR (B) and Western blotting (C). *N *= 5. (D) Collagen contraction assay. *N *= 5. (E) Boyden chamber transwell assay (100×). *N *= 5. (F) Cell proliferation was evaluated using EdU (200×). *N *= 5. (G) Principal component analysis plot. (H) Volcano plot. (I) Pathway analyses. (J) Gene set enrichment analysis. (K) DCF‐DA staining (200×) for ROS production in H‐HSC. *N *= 3. (L) The mRNA levels of pro‐oxidant and anti‐oxidant genes. *N *= 3. Data are expressed as the mean±SD. *p < 0.05, two‐tailed Student's *t*‐test.

Next, we sought to investigate the functional involvement of NFIB in the spontaneous activation of HSC and the potential mechanism in vitro (Figure 2A). Therefore, a lentivirus containing the NFIB plasmid was used to overexpress NFIB in primary human HSC (H‐HSC), resulting in significant dampening of H‐HSC activation, as demonstrated by the suppression of fibrotic molecules at both the mRNA and protein levels (Figure 2B,C). Gel contraction assays further revealed marked inhibition of the contractile ability of H‐HSC after NFIB overexpression (Figure 2D). Additionally, transwell assays (Figure 2E) and 5‐ethynyl‐2′‐deoxyuridine (EdU) incorporation assays (Figure 2F) confirmed that NFIB delayed the proliferation of H‐HSC. In line with H‐HSC regulation, NFIB overexpression in TGF‐β stimulated LX‐2 cells and effectively reduced the fibrosis response with the inhibition of cell proliferation, profibrotic protein expression, and cell construction (Figure S5). Collectively, these results suggested that NFIB may be a potential therapeutic target for inhibiting HSC activation. In addition, RNA‐seq experiments were performed to compare the transcriptomes of human primary HSCs cultured with or without lentivirus containing the NFIB (Figure S6). A principal component analysis indicated drastic alterations in the cellular transcriptome caused by NFIB overexpression (Figure 2G). Using *p* < 0.05 and a two‐fold change as a cutoff, 2086 differentially expressed genes (DEGs) were identified (Figure 2H). Gene Ontology (GO), Kyoto Encyclopedia of Genes and Genomes (KEGG, Figure 2I), and Gene Set Enrichment Analysis (Figure 2J; Figure S7) revealed that NFIB overexpression primarily affected the nod‐like receptor signaling and oxidative stress‐related pathways. Indeed, NFIB reduced the accumulation of ROS in HSCs (Figure 2K). Moreover, compared with control group, HSCs with overexpressed NFIB exhibited higher levels of antioxidant genes and lower levels of nicotinamide adenine dinucleotide phosphate (NAD(P)H) oxidase subunits (Figure 2L). These results indicate that NFIB restrains HSC activation through constricting oxidative stress.

### 2.3 HSCs‐Specific NFIB Overexpression Mitigates CDAHFD‐ and CCl4‐Induced Fibrosis in Vivo

**FIGURE 3:**
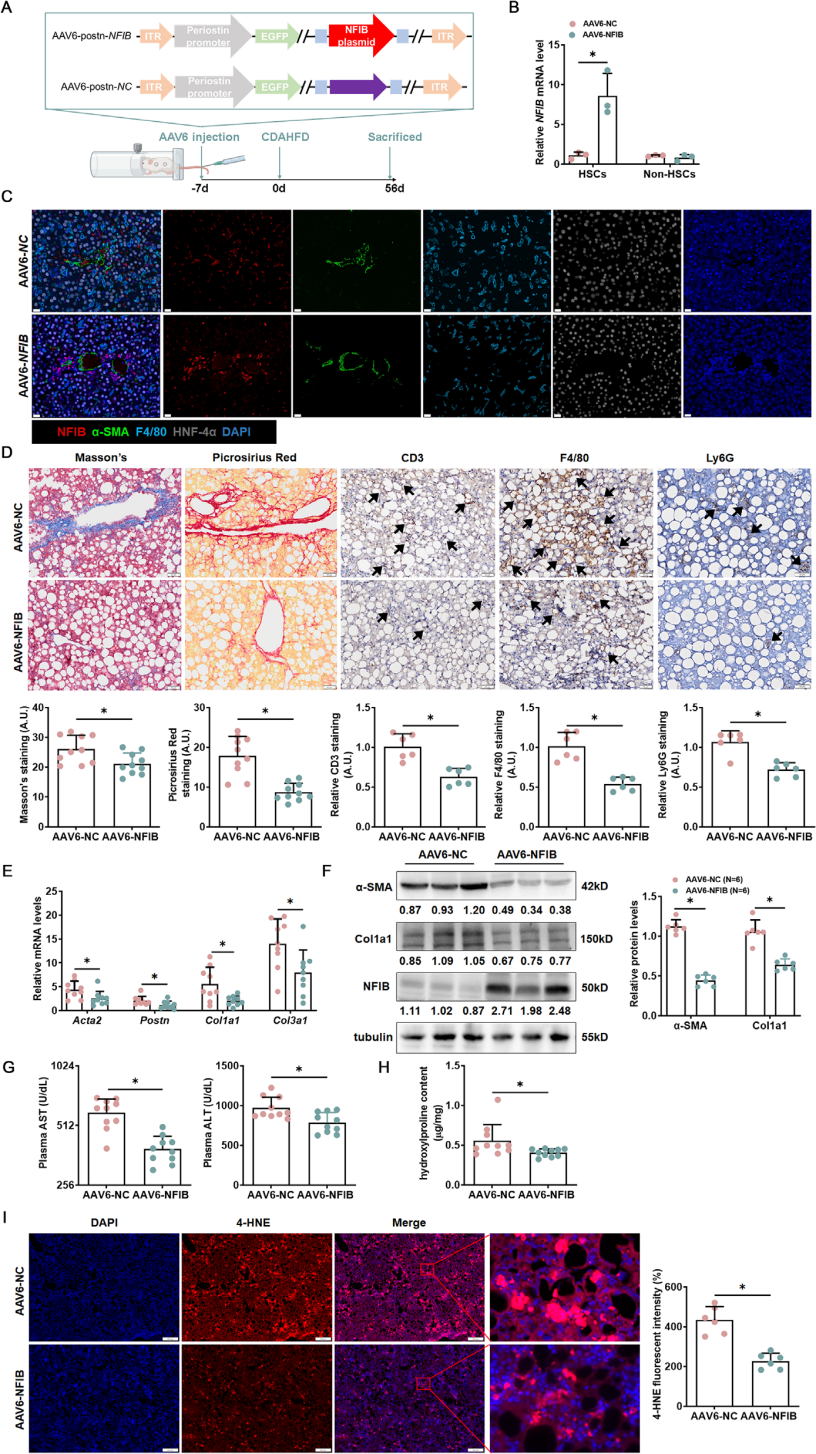
HSCs‐specific NFIB overexpression mitigates CDAHFD‐induced MASH fibrosis in vivo. C57BL/6 mice were injected with lentivirus carrying the overexpression plasmid targeting NFIB (AAV6‐NFIB) or control plasmid (AAV6‐NC), followed by administration with CDAHFD for 8 weeks. (A) Schematic of the animal protocol. (B) Primary HSCs were isolated, and NFIB expression in the “HSCs” and in the “Non‐HSCs” was examined by qPCR. *N *= 3. (C) Immunofluorescence staining (400×) was performed with the indicated antibodies in CDAHFD‐induced fibrosis tissues. (D) Paraffin sections were stained with picrosirius red (100×), Masson's trichrome (100×), and IHC (100×) (cluster of differentiation 3 [CD3], F4/80, lymphocyte antigen 6G [Ly6G]). *N *= 6–10. Scale bar: 50 µm. Hepatic profibrogenic genes were examined using quantitative PCR (E) and Western blotting (F). *N *= 6–9. (G) Plasma AST, ALT levels. *N *= 10. (H) Hepatic hydroxyproline levels. *N *= 10. (I) Immunofluorescence (IF)IHC staining (50×) for 4‐Hydroxynonenal (4‐HNE) expression in liver tissues. *N *= 6. Data are expressed as the mean±SD. *p<0.05, two‐tailed Student's *t*‐test.

To further investigate the impact of NFIB on liver fibrosis in vivo, mouse livers were injected with the AAV6‐NFIB virus to induce NFIB hyperexpression before the choline‐deficient, amino acid‐defined high‐fat diet (CDAHFD) challenge. In contrast to the AAV8 virus, the AAV6 virus targeted the liver interstitium. Thus, plasmid targeting NFIB or control plasmid (NC) were strategically positioned downstream of the *Postn* promoter before being included in AAV6 [23, 24] (Figure 3A). The NFIB level did dramatically increase in HSCs, but not in hepatocytes and macrophages (Figure 3B,C). Masson, picrosirius red staining, and histopathological analysis of liver sections revealed that hepatic fibrosis, macrophage, T cell, and neutrophil infiltration were collectively attenuated in AAV6‐NFIB‐injected mice, compared with AAV6‐NC‐injected mice (Figure 3D). Moreover, NFIB overexpression in HSCs effectively inhibited the gene and protein expressions of pro‐fibrotic molecules (Figure 3E,F). Furthermore, steatotic liver injury, using plasma alanine aminotransferase (ALT) and aspartate aminotransferase (AST) levels, was markedly alleviated as a result of NFIB overexpression (Figure 3G). Hepatic hydroxylproline quantification confirmed that overexpressed NFIB attenuated liver fibrosis in mice (Figure 3H). Similar observations were made in CCl_4_‐induced liver fibrosis (Figure S8).

Consistent with GO and KEGG analysis, TGF‐β promoted ROS accumulation in LX‐2 cells, while being reduced by NFIB treatments (Figure S9A). Similarly, pro‐inflammatory genes (interleukin‐1, interleukin‐6 [IL‐6], tumor necrosis factor‐α) were highly induced by TGF‐β, whereas being greatly inhibited after NFIB insult (Figure S9B). More interestingly, hydrogen peroxide (H_2_O_2_) impaired NFIB protein stability, leading to degradation; however, protein degradation was blocked by N‐acetyl‐l‐cysteine (NAC, Figure S9C). On the other hand, oxidative stress was highly restricted in NFIB overexpressed mice (AAV6‐NFIB) after CDAHFD challenge. As shown in Figure S9D, antioxidants, including superoxide dismutase (SOD), glutathione (GSH), and catalase (CAT), were strongly increased, while pro‐oxidants (H_2_O_2_ and malondialdehyde [MDA]) decreased in AAV6‐NFIB mice. Besides, AAV6‐NFIB mice showed a lower level of NAD(P)H oxidase subunits (Figure S9E) and a higher level of antioxidant genes (Figure S9F), compared with the control groups (AAV6‐NC). IF staining identified that lipid peroxidation product 4‐HNE (Figure 3I) was dramatically reduced in AAV6‐NFIB mice after CDAHFD challenge. Similar observations were made in CCl_4_‐induced liver fibrosis (Figure S10).

### 2.4 HSCs‐Specific Blocking NFIB Accelerates CDAHFD‐Induced MASH Fibrosis in Mice

**FIGURE 4:**
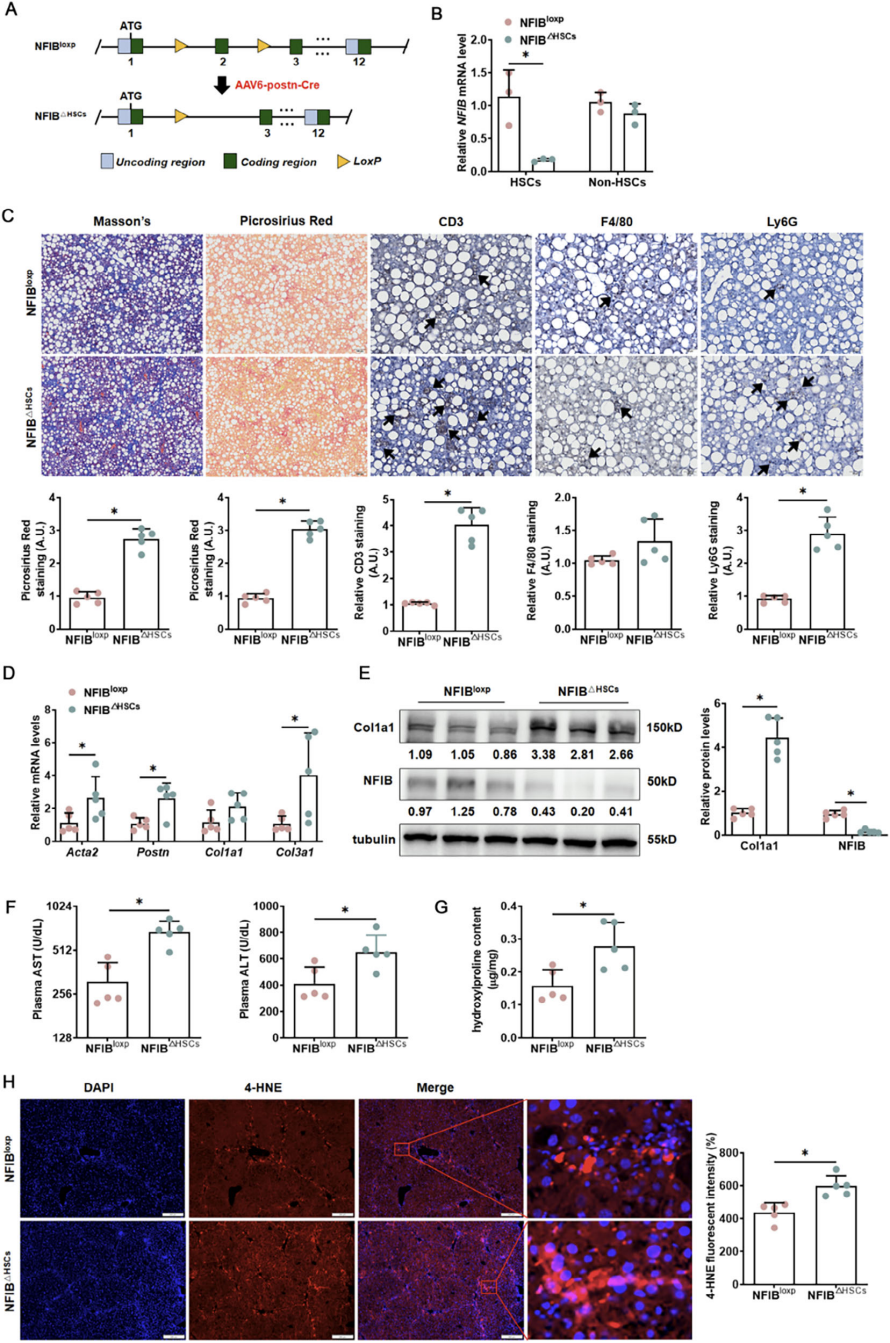
HSCs‐specific blocking NFIB accelerates CDAHFD‐induced MASH fibrosis in mice. NFIB LoxP mice were injected with lentivirus carrying CRE (NFIB^△HSCs^) or not (NFIB^loxp^) followed by administration with CDAHFD for 8 weeks. (A) Schematic of the animal protocol. (B) Primary HSCs were isolated, and NFIB expression in the “HSCs” and in the “Non‐HSCs” was examined by qPCR. *N *= 3. (C) Paraffin sections were stained with picrosirius red (50×), Masson's trichrome (50×) and IHC (100×) (CD3, F4/80, Ly6G). *N *= 5. Scale bar: 50 µm. Hepatic profibrogenic genes were examined using quantitative PCR (D) and Western blotting (E). *N *= 5. (F) Plasma AST, ALT levels. *N *= 5. (G) Hepatic hydroxyproline levels. *N *= 5. (H) IF staining (50×) for 4‐HNE expression in liver tissues. *N *= 5. Data are expressed as the mean±SD. *p<0.05, two‐tailed Student's *t*‐test.

Whether blocking NFIB in HSCs could further promote liver fibrosis, NFIB LoxP (NFIB^loxp^) mice were generated and subsequently administered the AAV6‐*postn*‐CRE virus to induce HSCs conditional blocking NFIB mice (NFIB^△HSCs^) (Figure 4A). As shown in Figure 4B, NFIB expression was reduced in HSCs following this intervention. Subsequent analysis utilizing Masson's trichrome and picrosirius red staining, along with histopathological examination of liver tissue sections, demonstrated increased hepatic fibrosis, as well as heightened infiltration of T cells and neutrophils in NFIB^△HSCs^ mice compared to NFIB^loxp^ mice (Figure 4C). Notably, the inhibition of NFIB in HSCs significantly upregulated the gene and protein expression levels of pro‐fibrotic molecules (Figure 4D,E). Moreover, NFIB inhibition in HSCs exacerbated steatotic liver injury in mice (Figure 4F). Hepatic hydroxylproline quantification further confirmed that NFIB blockade promoted liver fibrosis in this murine model (Figure 4G). 4‐HNE staining also confirmed the higher level of oxidative stress in NFIB^△HSCs^ mice (Figure 4H). These findings underscore the pivotal regulatory role of NFIB in the progression of liver fibrosis.

### 2.5 CCL5 is a Potential Target of NFIB During HSC Activation

**FIGURE 5:**
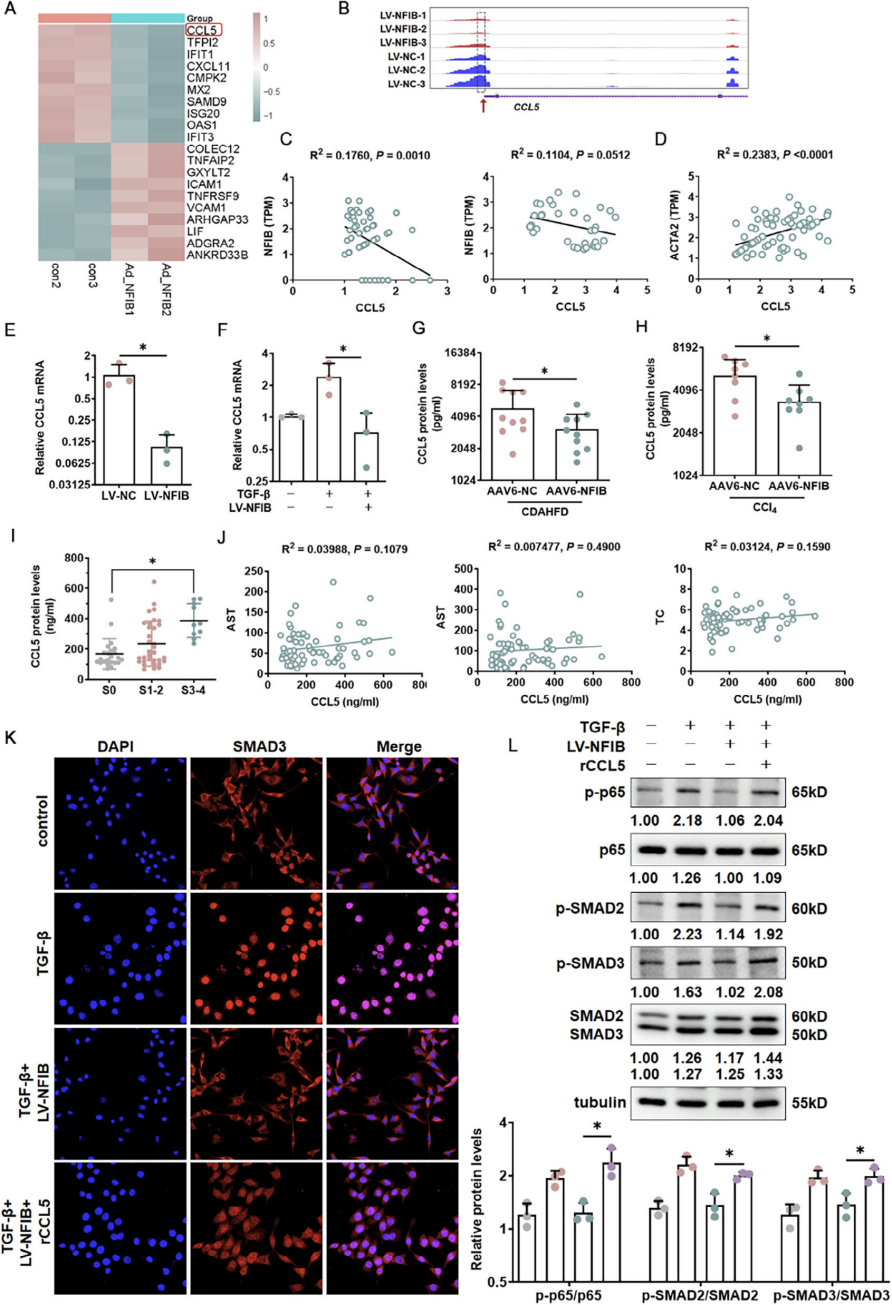
CCL5 is a potential target of NFIB during HSC activation. Primary human HSCs were transfected with overexpressed NFIB (LV‐NFIB) and control virus (LV‐NC) for 48 h. (A) Heatmap. (B) IGV plots of NFIB RNA‐seq showed the binding peaks at the CCL5 loci. (C, D) The NFIB, ACTA2, and CCL5 levels were detected in the single‐cell sequences of patients with cirrhosis (GSE147581) and MASH single‐cell sequence. The Pearson correlation was performed using GraphPad. (E) CCL5 mRNA level in primary H‐HSCs. *N *= 3. (F) CCL5 mRNA level in LX‐2 cells. *N *= 3. Elisa detects the release of CCL5 in CDAHFD‐induced mice (G) and CCl_4_‐induced mice (H) *N *= 8–10. (I, J) Plasma CCL5 protein expression in different fibrosis levels, as well as the correlation between CCL5 and AST, ALT, and TC, respectively. (K) IF staining (200×) for SMAD3 expression in LX‐2 cells. (L) Protein expressions of p‐p65, p65, p‐SMAD2, p‐SMAD3, SMAD2 and SMAD3 were examined using Western blotting. *N *= 3. Data are expressed as the mean±SD. Pearson was used for C and J. A two‐tailed Student's *t*‐test was used for E–I, K, and L. *p < 0.05.

To determine the transcriptional mechanism of the inhibition of HSC activation by NFIB, RNA‐seq experiments were performed to analyze the potential downstream of NFIB. Interestingly, RNA‐seq showed that C─X─C motif chemokines, which are key regulators of inflammatory and oxidative stress‐related pathways [25], were changed. Especially, in primary H‐HSCs, CCL5 expression was significantly decreased following NFIB overexpression (Figure 5A). RNA‐seq revealed decreased chromatin accessibility at several gene loci in human primary HSCs with lentivirus comprising the NFIB (Figure 5B). Moreover, a single‐cell analysis of GSE147581 and our MASH‐related fibrosis tissue samples confirmed that CCL5 was negatively correlated with NFIB in HSCs, while positively correlated with *ACTA2* (Figure 5C, D). Thus, we focus on CCL5 as the main target of NFIB during MASH‐related liver fibrosis. Indeed, HSCs‐specific NFIB overexpression resulted in lower CCL5 levels (Figure 5E,F). Of note, plasma CCL5 was highly inhibited in NFIB overexpressed mice under CDAHFD and CCl_4_ challenge (Figure 5G,H) compared to the control mice. Importantly, single cell sequence results of MASH‐related liver fibrosis also confirmed that CCL5 expression only strongly increased in HSCs (Figure S11). The expression of plasma CCL5 increased with the degree of fibrosis in patients. (Figure 5I), But there was no obvious correlation in indicating liver injury (Figure 5J). Previous studies have also shown that CCL5 is a key factor in promoting heart fibrosis [26]. Blocking the CCL5 receptor could reduce the level of renal fibrosis [27]. Therefore, whether CCL5 promotes MASH fibrosis through TGF‐β/Smad signaling pathway should be identified. As shown in Figure 5K,L, recombinant CCL5 (rCCL5) treatment accelerated the SMAD3 nuclear transfer and then upregulated the phosphorylation of p65, SMAD2, SMAD3. Collectively, these results indicated that NFIB/CCL5 axis might be the vital target in HSCs during the development of MASH fibrosis. Besides, the antioxidant effect of NFIB was found to depend on the suppression of CCL5. We found inhibiting CCL5 expression in HSCs reinstated NFIB's effect, while overexpressing CCL5 suppressed it in a dose‐dependent manner (Figure S12).

### 2.6 NFIB Transcriptionally Inhibits CCL5 Induction in HSCs

**FIGURE 6:**
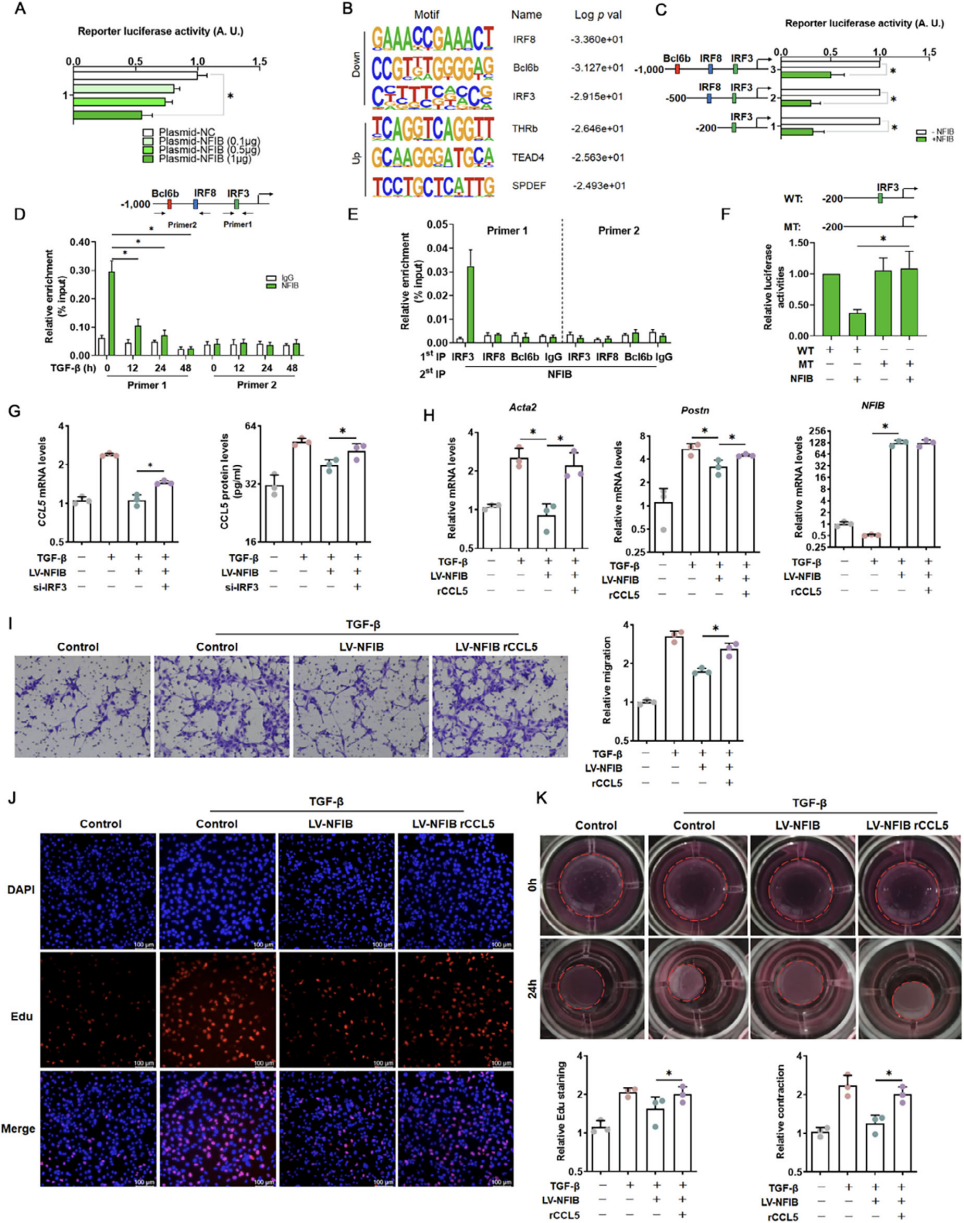
NFIB transcriptionally inhibits CCL5 induction in HSCs. (A) A CCL5 promoter luciferase (−1000) was transfected to LX‐2 cells with increasing doses of NFIB. Luciferase activities were normalized by protein concentration and green fluorescent protein. *N *= 3. (B) Hypergeometric optimization of motif enrichment analysis. (C) Truncated CCL5 promoter–luciferase constructs were transfected into LX‐2 cells with or without NFIB. Luciferase activities were normalized by protein concentration and green fluorescent protein. *N *= 3. (D) LX‐2 cells were treated with TGF‐β (2 ng/mL) and harvested at the indicated time points. ChIP assays were performed with anti‐NFIB or IgG. *N *= 3. (E) Re‐ChIP assays were performed with the indicated antibodies. *N *= 3. (F) Wild‐type and mutated CCL5 promoter‐luciferase constructs were transfected into HEK293 cells with or without NFIB. Luciferase activities were normalized by protein concentration and green fluorescent protein. *N *= 3. (G) The mRNA and protein levels of CCL5 were detected by PCR and ELISA. *N *= 3. LX‐2 cells were treated with recombinant CCL5 (20 ng/mL) for 24 h after NFIB overexpression. (H) The mRNA levels of *Acta2, Postn*, and *NFIB* in LX‐2 cells were measured by quantitative PCR. *N *= 3. (I) Boyden chamber transwell assay (100×). *N *= 3. (J) Cell proliferation was evaluated using EdU (200×). *N *= 3. (K) Collagen contraction assay. *N *= 3. Data are expressed as the mean±SD. *p < 0.05, two‐tailed Student's *t*‐test.

To further determine whether NFIB directly binds to CCL5, a luciferase reporter assay was performed. NFIB overexpression attenuated CCL5 promoter–luciferase reporter activity in a dose‐dependent manner (Figure 6A). Hypergeometric optimization of motif enrichment analysis indicated that interferon regulatory transcription factor 8 (IRF8), B cell CLL/lymphoma 6 member B (Bcl6b) and interferon regulatory transcription factor 3 (IRF3) ranked top among the transcription factors inhibited by NFIB treatment (Figure 6B). We then constructed serial deletions of CCL5 promoter–luciferase plasmid. As shown in Figure 6C, NFIB activated the CCL5 promoter, and this effect depended on a proximal segment (−200 to −10 bp relative to the TSS) harboring an IRF3 consensus motif. Consistent with this, chromatin immunoprecipitation (ChIP) assays in TGF‐β–stimulated LX‐2 cells confirmed NFIB binding specifically to the IRF3‐containing region of the CCL5 promoter, with no enrichment observed in distal promoter areas (Figure 6D). Re‐ChIP assays also confirmed that IRF3, but not IRF8 or Bcl6b, could be assembled into a complex with NFIB on the CCL5 promoter when LX‐2 cell was stimulated with TGF‐β (Figure 6E). Mutation of IRF3 motif, however, desensitized the CCL5 promoter to NFIB overexpression, again illustrating that NFIB might be recruited to the CCL5 promoter to inhibit its transcription depending on IRF3 region (Figure 6F). Indeed, once blocking IRF3 level in LX‐2 cells, the synthesis and release of CCL5 were highly raised even under NFIB treatment (Figure 6G). These results indicated that NFIB could directly bind to CCL5 promoter, which contains IRF3 motif, and then inhibit CCL5 expression. In addition, rCCL5 was used to further identify the pro‐fibrotic effects. As shown in Figure 6H, rCCL5 treatment upregulated pro‐fibrotic genes at mRNA level even under NFIB overexpressed cell. Transwell (Figure 6I), EdU (Figure 6J) and gel construction assays (Figure 6K) confirmed that rCCL5 treatment promoted the proliferation, migration and contractile ability of LX‐2 cell, which were being restricted after NFIB insult, identifying that CCL5 might be a downstream of NFIB during HSC activation. Moreover, the receptors of CCL5 were further confirmed. As shown in Figure S13, CCR5 was the main receptor of CCL5 under liver fibrosis conditions.

### 2.7 CCL5 neutralization Attenuates MASH Fibrosis in Mice

**FIGURE 7:**
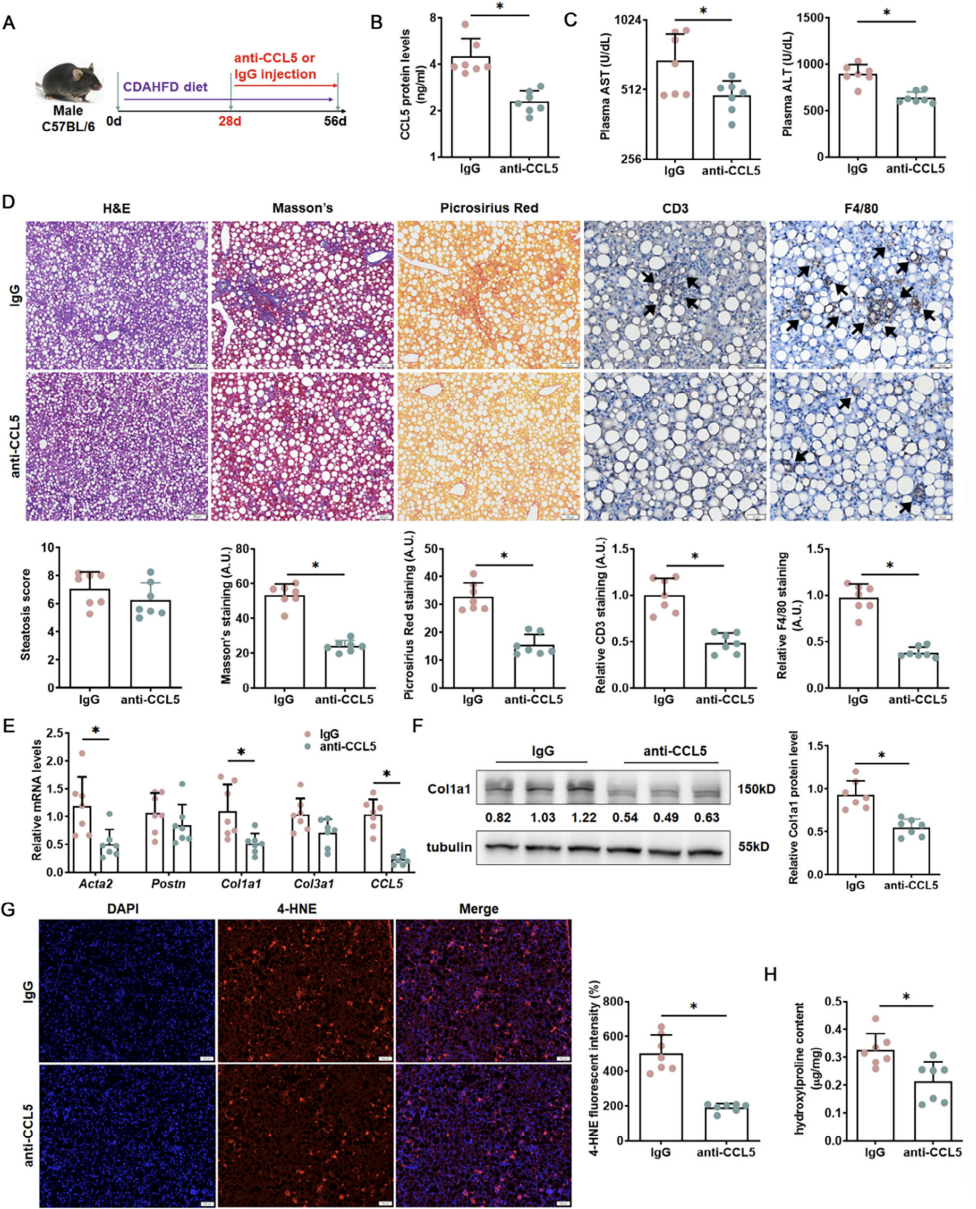
CCL5 neutralization attenuates MASH fibrosis in mice. C57BL/6 mice were fed on a CDAHFD diet for 8 weeks, and administration of the CCL5‐neutralizing antibody was started at the fifth week. (A) Scheme of protocol. (B) Plasma CCL5 level. *N *= 7. (C) Plasma AST and ALT levels. *N *= 7. (D) Liver sections were stained with H&E (50×), picrosirius red (50×), Masson's (50×), and IHC (100×) (CD3, F4/80) staining. *N *= 7. (E) Expression levels of NFIB and pro‐fibrotic genes were examined by qPCR. *N *= 7. (F) Protein expression of Col1a1 was examined using Western blotting. *N *= 7. (G) IF staining (50×) for 4‐HNE expression in liver tissues. *N *= 7. (H) Hepatic hydroxyproline levels. *N *= 7. Data are expressed as the mean±SD. *p < 0.05, two‐tailed Student's *t*‐test.

We then confirm the role of CCL5 in MASH fibrosis in vivo, and a neutralizing antibody was used after CDAHFD challenge (Figure 7A). The release of CCL5 was restricted after injection with anti‐CCL5 antibody (Figure 7B). CCL5 neutralization significantly improved hepatic function as measured by AST and ALT (Figure 7C). The therapeutic effect of the anti‐CCL5 antibody was further verified by histological staining. The results revealed a significant alleviation of immune cell infiltration and fibrosis in mice treated with the antibody compared to those receiving isotype IgG, whereas no marked improvement was observed in hepatic steatosis. (Figure 7D). qPCR (Figure 7E) and Western blotting (Figure 7F) confirmed that expression levels of myofibroblast markers were collectively downregulated in the liver as a result of CCL5 neutralization. Moreover, CCL5 neutralization significantly reduced oxidative stress (Figure 7G) and attenuated liver fibrosis in mice (Figure 7H). Combined, these data allude to the translational potential of CCL5 depletion in MASH fibrosis intervention.

### 2.8 Juglone Attenuates HSC Activation and MASH Fibrosis

**FIGURE 8:**
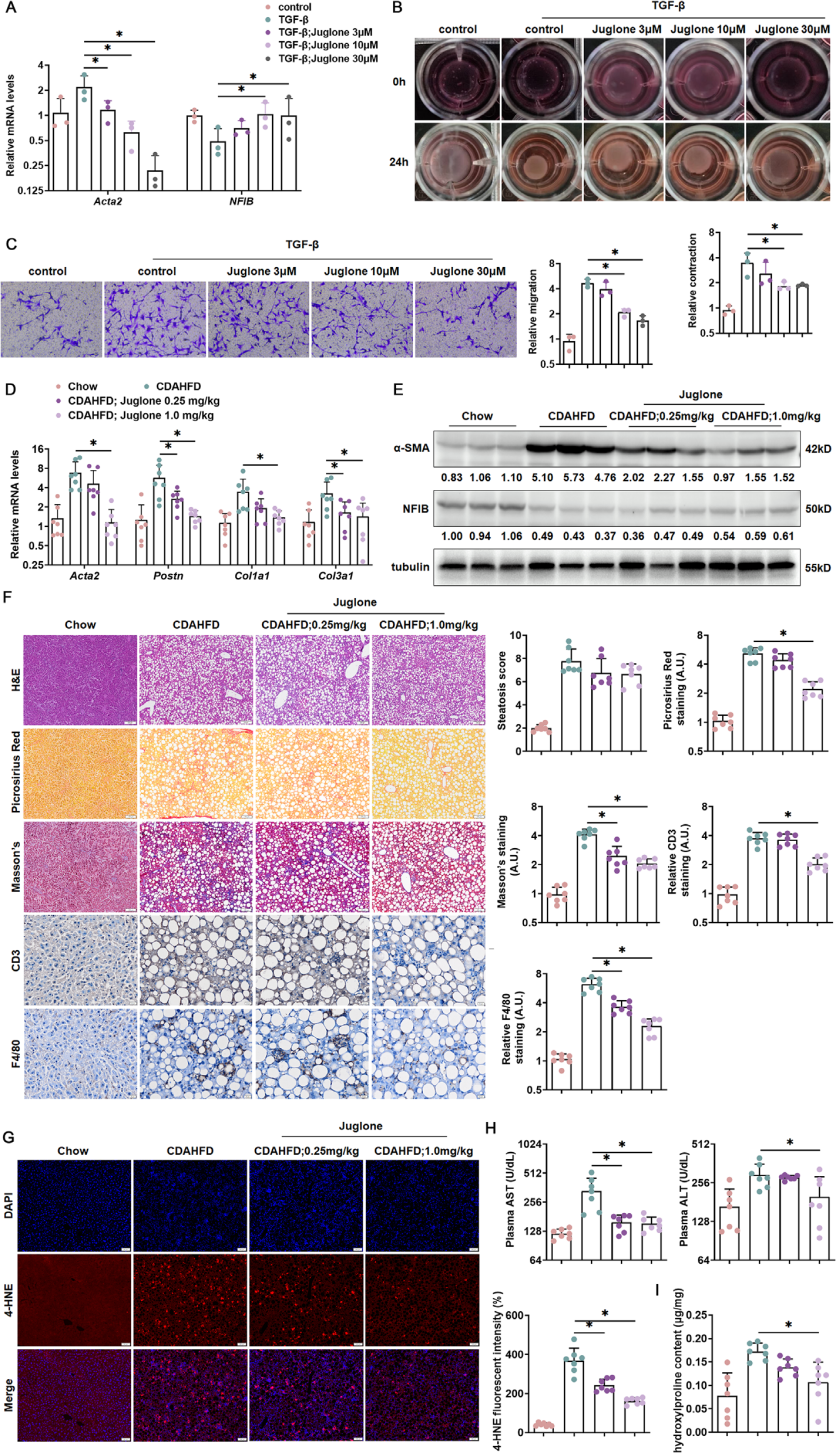
Juglone attenuates HSC activation and MASH fibrosis. (A‐C) LX‐2 cells were subjected to TGF‐β1 (2 ng/mL) treatment for 24 h with or without juglone treatments (3, 10, or 30 µm). Then, all cells were harvested for studies as follows. (A) The mRNA levels of *Acta2* and *NFIB* in LX‐2 cells were measured by qPCR. *N *= 3. (B) Collagen contraction assay. *N *= 3. (C) Boyden chamber transwell assay (100×). *N *= 3. (D–H) C57BL/6 mice were fed on an CDAHFD diet for 8 weeks, during which they concurrently received different doses of Juglone (0.25 and 1.0 mg/kg) via oral gavage. Hepatic profibrogenic genes in mouse liver tissues were examined using quantitative PCR (D) and Western blotting (E). *N *= 3–7. (F) Liver sections were stained with H&E (50×), picrosirius red (50×), Masson's (50×) and IHC (100×) (CD3, F4/80) staining. *N *= 7. (G) IF staining (50×) for 4‐HNE expression in liver tissues. *N *= 7. (H) Plasma AST and ALT levels. *N *= 7. (I) Hepatic hydroxyproline levels. *N *= 7. Data are expressed as the mean±SD. *p < 0.05, two‐tailed Student's *t*‐test.

To further confirm the significance of NFIB transformation, Juglone, an indirect agonist of NFIB [28, 29], was used. Indeed, Juglone upregulated the level of NFIB, and then inhibited pro‐fibrosis gene expression (Figure 8A). Gel construction and transwell analysis showed the same results of Juglone, which dramatically inhibits HSC activation (Figure 8B,C). Besides, injecting with a high dose of Juglone significantly downregulated the gene and protein expression levels of pro‐fibrotic molecules (Figure 8D,E). Histological stainings revealed a significant reduction in immune cell infiltration and fibrosis in mice treated with a high dose of Juglone, whereas its effect on steatosis was not pronounced. (Figure 8F). 4‐HNE staining also showed the lower level of ROS accumulation in both low and high doses of Juglone (Figure 8G). Furthermore, plasma ALT and AST levels were markedly alleviated as a result of Juglone administration (Figure 8H). Hepatic hydroxylproline quantification confirmed that Juglone injection attenuated liver fibrosis in mice (Figure 8I). All the data identified NFIB may be a potential target in the treatment of MASH fibrosis.

## 3 Discussion

Liver fibrosis represents a dynamic and progressive pathological process, marked by the aberrant deposition of ECM as a maladaptive consequence of the wound‐healing cascade triggered by hepatic injury, irrespective of etiology. Severe liver fibrosis profoundly influences patient outcomes and significantly elevates the risk of HCC [30]. Consequently, the identification of pivotal therapeutic targets capable of mitigating liver fibrosis is an urgent medical priority. Herein, we conducted an analysis of multiple single‐cell sequencing datasets and ultimately identified NFIB as a key target due to its pronounced alterations. Previous studies have reported NFIB's inhibitory effects on endothelial–mesenchymal transition and pulmonary fibrosis [20]. Nonetheless, its functional involvement in HSCs‐myofibroblast transition and liver fibrosis progression, as well as the underlying molecular mechanisms, remains unclear. In the present study, we observed a significant downregulation of NFIB expression in HSCs within fibrotic liver tissues and found that NFIB might exert a pronounced inhibitory effect on liver fibrosis by transcriptionally repressing CCL5 in HSCs.

The NFI family is a group of DNA‐binding transcription factors that serve as pivotal regulators in the development and maturation of multiple organ systems [31]. The absence of NFI family members can lead to a range of organ developmental disorders, particularly affecting the nervous system, resulting in structural brain anomalies, intellectual disabilities, nonspecific craniofacial anomalies, short stature, urinary tract defects, and so on [31, 32]. Recent studies have found that the dysregulation of NFI family members may also be closely related to other diseases. As a member of the NFI family, NFIB has also been extensively studied in the field of tumors in recent years. As a member of the NFI family, NFIB has been extensively studied in the field of oncology in recent years. Interestingly, NFIB exhibits divergent effects in tumorigenesis. On one hand, NFIB can inhibit tumorigenesis by promoting cell differentiation, such as high grade gliomas, lung adenocarcinoma and oral cancer [33, 34, 35]; on the other hand, NFIB can also facilitate tumorigenesis and metastasis, such as small cell lung cancer, prostate cancer and colorectal cancer, by altering chromatin accessibility, accelerating epithelial–mesenchymal transition (EMT) and changing the genomic landscape, which leads to the formation of a cancer epigenome [14, 36, 37]. Therefore, it is apparent that the role of NFIB is multifaceted and may differ according to the specific organ or cell type, with its functions not yet fully elucidated. In this study, we found that NFIB is also likely to be involved in the progression of liver fibrosis. We discovered that NFIB, as a transcription factor, could inhibit the transition of HSCs into myofibroblasts, thereby suppressing the activation of HSCs. This finding aligns closely with those of Pattnaik et al. in their investigation of pulmonary fibrosis, wherein they revealed that NFIB could mitigate lung fibrosis by inhibiting EMT [20]. However, their study did not explore how NFIB affects EMT. In our research, we proposed that the inhibitory effect of NFIB on the transition of HSCs into myofibroblasts might be associated with a reduction in CCL5 expression within HSCs. The administration of recombinant CCL5 significantly reversed the inhibitory effect of NFIB on HSC activation. However, considering the intricate nature of NFIB's role, it remains to be investigated whether NFIB exerts contradictory effects on the progression of fibrosis in other organs, akin to its influence on tumorigenesis.

The way NFIB exerts its effects is closely related to changes in chromatin accessibility. NFIB can establish a pro‐metastatic gene expression program by increasing the accessibility of distal regulatory elements of the genome, thereby promoting the upregulation of adjacent genes [14, 38]. Interestingly, in our study, we found that NFIB did not increase the chromatin accessibility of CCL5; rather, it decreased its accessibility and reduced CCL5 expression. This distinction may be related to the specific genes involved, as previous literature has found that NFIB could promote the expression of genes associated with metastasis, neuronal maturation, and stem cell identity [14, 38, 39], while its effects on CCL5 have not been reported. Additionally, it has also been reported that NFIB tends to bind to distal regions of genes, thereby increasing chromatin accessibility and regulating distal regulatory elements [14]; in our study, however, we found that it reduced chromatin accessibility at the proximal region of the CCL5 gene, which consequently decreased IRF3's binding at the CCL5 promoter region. Therefore, we speculate that this difference in chromatin accessibility may also be related to the different regions where NFIB acts (the gene proximal region versus the gene distal region), but further research is needed to confirm this.

Inflammation and oxidative stress can activate HSCs, thereby accelerating the process of fibrosis [40, 41, 42]. Research has reported that C─X─C motif chemokines are key regulators in the pathways regulating inflammation and oxidative stress [25]. In our study, we found that NFIB could transcriptionally inhibit the expression of CCL5 and significantly suppress inflammation and oxidative levels in fibrotic tissues. Moreover, recombinant CCL5 markedly reversed the anti‐fibrotic effects of NFIB. Therefore, we believed that NFIB might alleviate inflammation and oxidative stress by inhibiting the expression of CCL5 in HSCs, thereby delaying the progression of liver fibrosis. Nuclear erythroid 2‐related factor 2 (Nrf2) is a key transcription factor that protects against oxidative stress by upregulating antioxidative genes [43]. Studies have demonstrated that promoting the nuclear translocation of Nrf2 can effectively inhibit HSC activation, thereby ameliorating the progression of liver fibrosis [43, 44]. It is a surprising observation in our study that NFIB overexpression promoted the nuclear translocation of Nrf2 (Figure S14). The specific mechanisms underlying this process remain to be further elucidated. Our subsequent research will focus on the functional analysis of the NFIB/Nrf2 pathway. Interestingly, we also found that inflammation and oxidative stress might conversely affect the protein stability of NFIB. In this study, we observed that both H_2_O_2_ and IL‐6 significantly promoted the degradation of NFIB. Therefore, we proposed that there might exist a closed‐loop cycle of NFIB‐inflammation/oxidative stress‐NFIB in HSCs, which had not been reported before. Specifically, the low expression of NFIB in fibrotic tissues leads to a decreased inhibitory effect on inflammation and oxidative stress, which in turn further promotes the degradation of NFIB. This creates a vicious cycle that continuously exacerbates inflammation, oxidative stress, and the fibrosis process. Given that fibrosis is always accompanied by inflammation and oxidative stress [45], we speculate that the low expression of NFIB during the progression of liver fibrosis may be caused by the inflammation and oxidative stress resulting from multiple factors. These findings not only provide novel insights into fibrosis progression but also position the NFIB/CCL5 axis as a promising therapeutic target for breaking this pathological loop, offering substantial potential for developing targeted anti‐fibrotic strategies and prognostic biomarkers.

However, our study also has some limitations. For instance, we did not investigate the direct and detailed mechanism of action between NFIB and IRF3. Additionally, we did not validate whether the inhibitory effect of NFIB on liver fibrosis is mediated by CCL5 in animal experiments. In our future research, we will address these issues further.

## 4 Conclusion

In summary, our research uncovered a previously unidentified NFIB/CCL5 axis in HSCs and confirmed its significant role in liver fibrosis. This discovery provided critical insights into the regulation of damage‐induced cell redox stress and inflammation, offering a deeper understanding of the molecular mechanisms underlying liver fibrosis. Meanwhile, several questions have arisen for our consideration. Given that fibrosis also serves as a mechanism for tissue repair, we must consider whether NFIB may also impede the repair process of injury or if it exclusively inhibits excessive fibrosis. Moreover, the majority of literature suggests that NFIB may have the potential to promote tumorigenesis and metastasis. Therefore, when selecting NFIB as a therapeutic target for liver fibrosis, we should assess whether this choice could elevate the risk of tumor development. These questions may represent valuable directions for future research. Altogether, we believe that NFIB is a highly promising target for the treatment of liver fibrosis.

## 5 Materials and Methods

### 5.1 Patient Samples

Liver samples of patients with chronic liver disease and different liver fibrosis stages were obtained during liver puncture. The Scheuer score (range, S0–S4) was used to evaluate the liver fibrosis stages. Written informed consent was obtained from patients included in this research. This research protocol received ethical approval from the Institutional Review Board of Nanjing Drum Tower Hospital (Approval No. 2024‐482‐02) and was conducted in accordance with the ethical principles of the Declaration of Helsinki (1975). Demographic and clinical characteristics of the study participants are summarized in Table S1.

### 5.2 Animals

Male wildtype (WT) C57BL/6 mice (6 weeks old; body weight, 20–22 g) were obtained from Nanjing Medical University (Nanjing, China). All mice were fed sufficient water and food in a pathogen‐free environment with a constant temperature and humidity (23 ± 2°C; 50% − 60%) and a standard 12‐h dark–light cycle at Southeast University Laboratory Animal Center (Nanjing, China). The animal study protocol was approved by the Ethics Committee of Nanjing Drum Tower Hospital (no. 2024AE01018).

To establish the liver fibrosis model, mice were subjected to CCl_4_ administration (1.0 mL/kg body weight as 50% volume/volume, twice per week for 3 weeks), BDL, and feeding with CDAHFD for 8 weeks [46, 47]. A BDL model was established in mice (n = 5) anesthetized with isoflurane. A midline laparotomy (∼1 cm) was performed to expose the common bile duct, which was then doubly ligated with 5–0 silk sutures and transected between the ligatures. The peritoneal layer was reapproximated, followed by sequential closure of the muscle and skin layers using the same suture material. In sham‐operated controls, the common bile duct was isolated but not ligated before abdominal closure. Primary HSCs were isolated from mice at 2 weeks post‐procedure. The CDAHFD (#CDAHF60, Dyets, Wuxi, China) contained 60% kcal from fat, 0.1% methionine, and lacked choline supplementation. To construct HSCs‐specific NFIB‐overexpressing mice, AAV6*‐postn‐*LV*NFIB* virus and a corresponding control virus (AAV6*‐postn‐*LV*NC*) (1 × 10^11^ VG/g; Nanjing Biotech Co., Ltd., Nanjing, China) were injected into the tail vein before the liver fibrosis model was constructed. The NFIB^loxp^ mice were generated by introducing the LoxP sites to flank exon 2 of the NFIB alleles (GemPharmatech Co., Ltd, Nanjing, China), and then injected the AAV6‐*postn*‐CRE virus by tail vein to induce HSCs conditional blocking NFIB mice (NFIB^△HSCs^). Additionally, we constructed a BDL model to investigate the expression levels of NFIB in mice with cholestasis‐induced liver fibrosis. Details can be found in the supplementary material.

Juglone (CAS: 481‐39‐0; ≥98% pure), purchased from MedChemExpress LLC (Shanghai, China), was dissolved in ethanol. All mice were randomly assigned to one of four groups: (1) control group; (2) CDAHFD group; (3) CDAHFD + low‐dose juglone (0.25 mg/kg); (4) CDAHFD + high‐dose juglone (1 mg/kg). The mice received oral administration of juglone at the specified doses.

### 5.3 Single‐Cell Suspension Preparation

Following collection, liver tissue specimens were promptly immersed in ice‐cold University of Wisconsin solution for transportation. Upon arrival at the laboratory, the tissues were enzymatically dissociated into single‐cell suspensions using the sCelLiVE Tissue Dissociation Solution, according to the manufacturer's instructions.

### 5.4 10×Genomics 3’ Single‐Cell RNA‐seq

Single‐cell suspensions were prepared for 3' RNA sequencing using the 10× Genomics Chromium platform. Following the manufacturer's guidelines, we isolated 10 000–15 000 viable cells and loaded them onto a Single Cell A Chip. Subsequently, cells were co‐encapsulated with barcoded gel beads in emulsion droplets using the Chromium controller for reverse transcription. The process continued with cDNA amplification, library fragmentation, and the ligation of 5' adapter and sample index. The final libraries were subjected to sequencing on an Illumina HiSeqX platform.

### 5.5 Analysis of Single‐Cell RNA‐seq Data

Alignment of the sequencing reads against the GRCh38 reference genome (Ensembl release 88) was performed with CellRanger (version 3.0.2) using default parameters. Quality control filtered cells expressing <500 genes, with unique molecular identifier (UMI) counts <800 or >8000, or >10% mitochondrial UMIs. Data integration was performed using Seurat V3 (v3.1.1). Datasets were split by donor, log‐normalized, and 2000 variable features were selected per subset using the ‘vst’ method. Integration anchors were identified with normal samples as reference, followed by batch correction using IntegrateData (30 dimensions). The integrated data were scaled, regressing out mitochondrial percentage and total UMIs. PCA was conducted with component selection guided by elbow plot visualization. Clustering was performed using the top 30 PCs (FindNeighbors; FindClusters, resolution = 0.5) and visualized via UMAP (n.neighbors = 40, dims = 1:30, min.dist = 0.3). Cluster markers were identified (FindAllMarkers; min.pct = 0.3, logfc.threshold = 0.6) for manual annotation, excluding multiphenotype doublets. Subclustering of specific cell types employed population‐specific parameters (PCs: 15–25; resolution: 0.3–1.2), with subsequent removal of high‐gene/UMI doublets.

We utilized the Seurat package's “FindMarkers” function with the MAST method to identify differentially expressed genes (adj. *p* < 0.05, |logFC| > 1, min.pct > 0.1). Following conversion of gene symbols to Entrez IDs, significant GO terms and KEGG pathways (*p* < 0.05) were identified using the clusterProfiler package (v3.9.2).

### 5.6 Data Collection and Preprocessing of Single‐Cell RNA‐seq Datasets

Gene expression data from three publicly available single‐cell RNA‐seq datasets (GSE136103, GSE147581, GSE174748, GSE136103, GSE147581 and GSE174748) were obtained from the Gene Expression Omnibus (GEO; http://www.ncbi.nlm.nih.gov/geo). Subsequent pre‐processing of the raw data, encompassing background correction, normalization, data summarization, and gene probe annotation, was conducted utilizing the R programming environment (version 4.4.2). In the process of analyzing the data of each single‐cell datasets, we regard the activated HSCs as a kind of cell group of concern and mark them with biomarkers (“Aebp1,” “Angptl6,” “Bgn,” “C4b,” “Col14a1,” “Col1a1,” “Col1a2,” “Col3a1,” “Colec11,” “Lrat,” “Igfbp6,” “Igfbp5,” “Ifitm1,” “Cxcl12,” “Acta2”).

### 5.7 Pseudo‐Time Analysis

The differentiation potential of individual cells was quantified with the CytoTRACE2 package (version 1.0.0) and visualized via UMAP. An inferred root state, comprising cells with the maximum CytoTRACE2 scores, was established for trajectory analysis. Pseudotemporal ordering and lineage inference were subsequently performed using the Monocle package (version 2.28.0) to model the developmental pathways of cell subtypes.

### 5.8 Cell Culture and Treatment

Human immortalized hepatic stellate cells (LX‐2) were sourced from American Type Culture Collection (Manassas, VA, USA) and routinely cultured in Dulbecco's modified Eagle's medium (DMEM) (Gibco, Gaithersburg, MD, USA) supplemented with 10% fetal bovine serum (FBS). All cells were cultured in a 5% CO_2_ incubator at 37°C.

Prior to TGF‐β (2 ng/mL; #246‐LP‐025; R&D System, Minneapolis, MN, USA) stimulation, LX‐2 cells were subjected to an overnight serum starvation, which imitated HSC activation in vitro [48]. During certain experiments, recombinant human CCL5 protein (20 ng/mL; #HY‐P7282, MedChemExpress) was added to DMEM with TGF‐β for 24 h. Primary human HSCs (H‐HSCs) were obtained from Lonza Bioscience (Walkersville, MD, USA) and maintained in the culture medium supplied by the vendor.

To overexpress NFIB in LX‐2 and primary human HSCs, recombinant lentivirus expressing FLAG‐tagged NFIB protein was generated by Nanjing Biotech and directly added to the culture medium (LX‐2: multiplicity of infection = 40; primary human HSCs: multiplicity of infection = 80). After 48 h, the medium containing the lentivirus was discarded and further treatment was conducted. The expression sequence fragments pertaining to NFIB is atgatgtattctcccatctgtctcactcaggatgaattccacccatttattgaggcacttcttcctcacgtccgtgcaattgcctatacttggttcaacctgcaggctcgaaaacgcaagtactttaaaaagcatgagaaacgaatgtcgaaggatgaagaaagggcagtcaaagacgagctgctcagtgagaagcccgaaatcaagcagaagtgggcatccaggctcctggccaaactgcgcaaagatatccgccaggagtaccgggaggactttgtgcttaccgtgactggcaagaagcacccgtgctgtgtcttatccaatccagaccagaagggtaagattaggaggatcgactgcctgcgacaggcagacaaagtctggcgtctggatctagtcatggtgatcctgttcaaaggcatccctttggagagtacggatggagagcgactcatgaagtccccgcactgcacaaacccagcactttgtgttcagccacaccacatcacagtatcagttaaggagcttgacttgtttttggcatactacgtgcaggagcaagattctggacaatcaggaagtccaagccacagtgatcctgccaagaatcctccagggtacctcgaggacagctttgtaaaatccggagtcttcaatgtatcagagcttgtgagagtatccagaacacccataacccagggaactggagtcaacttcccaatcggagaaattcccagccaaccatactatcatgacatgaactctggtgtgaacctgcagaggtcgctgtcttctccaccgagcagcaaaagacccaaaactatatctatagatgaaaatatggagccaagtcctacaggagacttttacccctctccaaattcaccagctgctggaagtcgaacatggcatgaacgagatcaagatatgtcttctccaactacaatgaagaagcctgagaagccactgtttagctctacatctccacaggattcttccccaagattgagcactttcccccagcaccatcatcccggaatacctggagtcgcgcacagtgtcatctcaactcgaactccacctccgccctcaccgttgccatttccgacgcaagctatccttcctccggcaccttccagctacttctctcatccaacaatcagatatcctcctcacctgaatcctcaggatactctgaagaactacgtaccttcttatgacccatccagtcctcaaacgagccagcctaacagcagtggtcaagtagtagggaaagtgcctggccatttcacacctgtcttggcaccctctccccatcccagtgcagtgcgacctgtgaccctgaccatgacagatactaaacccatcactacatccactgaaggtgaggcagcttcacctacagcaaccacctacacagcctcaggcacatctcaagccaatcgatatgtgggactaagcccaagagacccatccttcctgcatcagcaacagctgaggatttgtgactggaccatgaatcaaaacggcaggcatttataccccagtaccagtgaggatacattgggaattacttggcaaagtcctggtacctgggctagcttggttccttttcaagtgtcaaataggacacccatcttaccggcaaatgtccaaaattatggtttgaacataattggagagcctttccttcaagcggagacaagcaac. Moreover, CCL5‐overexpressing lentivirus (LV‐CCL5), obtained from Nanjing Biotech, were used to transfect LX‐2 cells at different doses. In the juglone intervention experiment, LX‐2 cells were co‐treated with TGF‐β1 (2 ng/mL) and varying concentrations of juglone (3, 10, 30 µm) for 24 h prior to harvesting for subsequent analysis.

### 5.9 Primary Hepatocytes and HSCs Isolation

Primary murine hepatocytes and HSCs were isolated from WT and CCl_4_‐induced *C57BL/6* mice as previously described [49]. Mice were anesthetized and subjected to in situ perfusion via the portal vein using pre‐warmed calcium‐ and magnesium‐free HBSS to flush out circulating blood cells. Subsequent perfusion was carried out using HBSS containing Ca^2^⁺/Mg^2^⁺ and 0.03% collagenase IV (Cat# AF788, R&D Systems, Minneapolis, MN, USA) to digest the hepatic extracellular matrix. The liver was then promptly excised and placed in DMEM medium for cell dissociation. The resulting cell suspension was processed by filtration through a 70 µm nylon mesh and centrifugation (400 ×g, 8 min). The pellet was resuspended in 50% Percoll (Cat# 17089109, Cytiva, Pittsburgh, PA, USA)/DMEM solution and further centrifuged at 1200 rpm for 3 min to isolate hepatocytes. The supernatant enriched with non‐parenchymal cells was collected for subsequent purification of HSCs by Nycodenz density gradient centrifugation. Isolated primary hepatocytes and HSCs were cultured in high‐glucose DMEM with 10% FBS and 1% penicillin/streptomycin. The HSCs were cultured for 7 days to allow for spontaneous activation, as previously described [40]. All cells were cultured in a 5% CO_2_ incubator at 37°C.

### 5.10 siRNA Transfection

For siRNA transfection, recipient cells were incubated with 20 pmol of siRNA against CCL5 using RNAiMAX reagent (# 13778‐075, ThermoFisher Scientific, Waltham, MA, USA), while control cells received a non‐targeting siRNA. Briefly, the siRNA was mixed with RNAiMAX in Opti‐MEM medium and incubated for 15 min to form complexes. Then, the resulting complex was administered to the culture medium for 6 h before replacement with fresh medium. Subsequent experiments were performed 24 h following transfection. The siRNA targeting CCL5 (sense: 5'‐GUGCCCACGUCAAGGAGUATT‐3') was synthesized by General Biotechnology, Chuzhou, China.

### 5.11 RNA Isolation and Real‐Time PCR (qPCR)

RNA was extracted from cells and mouse liver samples using RNAiso reagent (#9109; Takara, Kyoto, Japan) according to the manufacturer's instructions and reverse‐transcribed using the HiScript two‐step method (#R222‐01; Vazyme, Nanjing, China). Then, a quantitative PCR was performed using SYBR Green (#Q411‐02; Vazyme) and an ABI PRISM 7900HT system (Applied Biosystems). The results were determined using fluorescence curves and quantified using the cycle threshold value and a standard curve. The mRNA values for each gene were normalized to 18s. The primer sequences (General Biotechnology, Chuzhou, China) used during this study are listed in Table S2.

### 5.12 Protein Extraction and Western Blotting

Liver or cell homogenates were prepared using RIPA lysis buffer (KGB5203‐100; KeyGEN BioTECH, Nanjing, China) with an added protease inhibitor (#A32955; ThermoFisher Scientific, Waltham, MA, USA). After bicinchoninic acid assay quantification (#23225; ThermoFisher Scientific) according to the manufacturer's protocols, equivalent amounts of protein were run on a 10% polyacrylamide gel (#E303‐C1; Vazyme) and transferred to nitrocellulose filter membranes (#88018; ThermoFisher Scientific). The membranes were first blocked with 5% skim milk for 1 h at room temperature. Then, they were incubated with the primary antibody at 4°C overnight, followed by a 1‐h incubation with the horseradish peroxidase‐conjugated secondary antibody. Subsequently, the blots were visualized in an exposure apparatus (Tanon, Shanghai, China) using an electrochemiluminescence kit (#E422‐C1‐P1; Vazyme). Finally, protein expression levels were calculated as grey values (version 1.52v; ImageJ) and standardized to ɑ‐tubulin. The details of all antibodies used in this study are listed in Table S3.

### 5.13 Biochemical Analysis

After the two models were completed, blood samples were collected by retro‐orbital bleeding and were centrifuged at 3500 rpm for 10 min to obtain supernatant, which was then preserved at −80°C for further analysis. The liver samples were either stored at −80°C or fixed with 4% paraformaldehyde for histological analysis.

The levels of serum/liver lipid (total cholesterol [TC; #A111‐1‐1] and triglycerides [TG; #A110‐1‐1]), hydroxylproline content (#A030‐2‐1), and liver/cells oxidative stress markers (MDA [#A003‐1‐2], SOD [#A001‐3‐2], CAT [#A007‐1‐1], H_2_O_2_ [#A064‐1‐1], and GSH [#A006‐2‐1]) were examined using standard enzymatic procedures following the manufactures’ protocols (Nanjing Jiancheng Bioengineering Institute, Nanjing, China). Serum ALT (#BC1555, Solarbio, Beijing, China), AST (#BC15165, Solarbio, Beijing, China), and CCL5 levels (#ml002135v, Mlbio, Shanghai, China) were also determined. Finally, the absorbance values were determined with a microplate spectrophotometer (SpectraMax iD3, Molecular Devices, USA).

### 5.14 Reactive Oxygen Species Production

After the treatments were applied, cellular reactive oxygen species were determined using viable cells incubated with 10 µm of dichlorofluorescin diacetate dye (#S0033M; Beyotime Biotechnology, Shanghai, China) for 20 min at 37°C. The treated cells were washed with PBS and stained with Hoechst at room temperature in the dark for 10 min. Images were captured using a fluorescent microscope.

### 5.15 H&E, Masson's Trichrome and Sirius Red Staining

Liver tissues were fixed with 4% paraformaldehyde for 24 h, dehydrated with anhydrous ethanol at sequential concentrations of 70%, 80%, 90%, and 100%, embedded in paraffin blocks, sectioned transversely (thickness, 5 µm), and stained for routine histology. All slices were dewaxed and hydrated before staining. H&E(#G1005‐100ML; Servicebio Technology, Wuhan, China), Masson (#G1006‐100ML; Servicebio Technology, Wuhan, China), and Sirius (#RS1240; G‐CLONE, Beijing, China) staining were performed according to the manufacturer's instructions. After the slices were sealed using neutral gum, the pathological changes were assessed and recorded using a Leica DMI 3000 microscope (Leica, Wetzlar, Germany).

### 5.16 IHC and IF

IHC testing was performed using antigens that were retrieved by heating with sodium citrate. Following permeabilization (1% Triton X‐100, 10 min) and peroxidase blockade (30% H_2_O_2_, 15 min), sections were blocked with 5% goat serum (#AR0009; Boster, Wuhan, China) and incubated with primary antibodies (CD3, F4/80, Ly6G, NOD‐like receptor thermal protein domain associated protein 3; 1:200 diluted with phosphate‐buffered saline [PBS]Ed) overnight at 4°C. After sections were washed, they were incubated with secondary antibodies for 1 h at room temperature, blocked using the 3,3′‐diaminobenzidine substrate kit (#SK‐4100; Vector, Torrence, CA, USA), and counterstained with hematoxylin. Images were photographed using a microscope. The collagen regions and positive signal expression areas were determined using Image Pro Plus software (Media Cybernetics, Rockville, MD, USA). Regarding IF staining, the liver sections were incubated with NFIB antibody (1:200 dilution) and 4‐HNE antibody (1:200 dilution) respectively at 4°C overnight after processing using the same steps as those used for immunohistochemistry. Secondary antibodies tagged with Alexa Fluor 488 and 594 were co‐incubated for 1 h. Nuclear labeling was performed using DAPI staining, and protein expression was observed using a confocal microscope (Leica DMI 3000). NFIB presented as green fluorescence, and 4‐HNE presented as red fluorescence. All antibody details are listed in Table S3.

### 5.17 Gel Constraction

The treated cells were trypsinized, centrifuged, and suspended in 1 mL of DMEM to achieve a concentration of 1.0×10^6^ cells/mL. Rat tail collagen type 1 (#354236; Corning, Bedford, MA, USA), 0.1% acetic acid solution, and cell suspensions were added to sterile tubes. Immediately thereafter, 1 m NaOH solution was added and mixed thoroughly until the liquid turned pink. Finally, the mixed gel was added to a 48‐well plate, and photographs were obtained using a digital camera at specific time points.

### 5.18 Migration Assay

After treatments were applied, the cells were seeded in upper transwell chambers (8‐µm pores, 24‐well format; BIOFIL, Guangzhou, China) in serum‐free DMEM. The cells were incubated for 24 h with specific culture medium in the lower chambers according to the specific experimental groups. Following fixation with 4% paraformaldehyde, migrated cells were stained by 0.1% crystal violet (#KEG1202‐100; KeyGEN BioTECH). Images were obtained using a Leica DMI 3000 microscope (10× magnification).

### 5.19 EdU Incorporation Assay

To evaluate cell proliferation, a EdU incorporation assay (#C0078S; Beyotime) was performed. Briefly, after treatment, the cells were seeded in 48‐well plates and maintained in a specific culture medium for 24 h, and EdU solution for 3 h. After nuclear staining was performed according to the manufacturer's instructions, fluorescence was detected using a Leica DMI 3000 microscope (20× magnification).

### 5.20 Luciferase Reporter Assay

We purchased a CCL5 promoter–luciferase from General Biol Company (Anhui, China), which was constructed by performing PCR amplification of genomic DNA encompassing the proximal promoter region and the first exon of the CCL5 gene (−1000/+10), subsequently cloned into the pGL3 basic vector (Promega, Madison, WI, USA). Transient transfections were performed using Lipofectamine 2000 (Invitrogen, Waltham, MA, USA), and luciferase activity was measured 24 to 48 h after transfection using a luciferase reporter assay system (Promega) according to established protocols.

### 5.21 Chromatin immunoprecipitation

Chromatin immunoprecipitation assays were performed according to previously established methods [50]. Briefly, chromatin cross‐linking was achieved with 1% formaldehyde for 8 min at room temperature, followed by sequential washing with ice‐cold PBS, Solution I (10 mm HEPES, pH 7.5, 10 mm EDTA, 0.5 mm EGTA, 0.75% Triton X‐100), and Solution II (10 mm HEPES, pH 7.5, 200 mm NaCl, 1 mm EDTA, 0.5 mm EGTA). Cells were lysed in buffer containing 150 mm NaCl, 25 mm Tris (pH 7.5), 1% Triton X‐100, 0.1% SDS, and 0.5% deoxycholate and complemented with a protease inhibitor cocktail. Sonication with a Branson 250 sonicator was used to fragment DNA into approximately 500‐bp segments. For each immunoprecipitation, lysates containing 100 µg of protein were used in conjunction with specific antibodies, followed by capture using protein A/G PLUS agarose beads (Santa Cruz Biotechnology, Santa Cruz, CA, USA). After sequential washing with RIPA buffer, high‐salt buffer, LiCl buffer, and Tris‐EDTA buffer, protein‐DNA cross‐links were reversed by incubating samples at 65°C overnight. Proteins were digested with proteinase K (Sigma, Burlington, MA, USA), and DNA was purified by phenol/chloroform extraction and ethanol precipitation. Enriched DNA was analyzed by real‐time PCR amplification with primers provided in Table S4. Input chromatin (10% of the initial material) served as the internal control.

### 5.22 RNA Sequencing and Data Analysis

RNA‐sequencing was performed as previously described [51]. Total RNA was extracted from primary human HSCs using RNAiso reagent (#9109; Takara, Kyoto, Japan) and underwent inspection for purity; quantification and integrity were determined using a NanoDrop 2000 spectrophotometer (ThermoFisher Scientific) and Agilent 2100 Bioanalyzer (Agilent Technologies, Santa Clara, CA, USA), respectively. Libraries were prepared according to the manufacturer's protocols using the TruSeq Stranded mRNA LT Sample Prep Kit (Illumina, San Diego, CA, USA), and sequencing was conducted using an Illumina HiSeq X Ten platform. Following initial acquisition, raw sequencing reads in FASTQ format were processed with Trimmomatic for quality control. Subsequently, high‐quality reads were aligned to the GRCh38 reference genome (Ensembl release 88) using HISAT2. The FPKM and read counts of each gene were obtained using Cufflinks and HTSeq count. The DESeq (2012) R package was utilized for DEGs during each pairwise comparison (*p*<0.05; fold change >2). We performed hierarchical clustering of DEGs to visualize sample‐specific expression patterns, followed by GO and KEGG enrichment analysis using a hypergeometric test to identify significantly overrepresented biological functions and pathways.

### 5.23 Statistical Analysis

Data are expressed as mean ± SD. Analyses used GraphPad Prism 8.0: two‐group comparisons by two‐tailed *t*‐test. Correlation analysis was performed using Pearson. Significance was set at *p* < 0.05. Sample sizes (n) are detailed in figure legends.

### 5.24 Study Approval

All animal experiments were performed in strict compliance with NIH guidelines and approved by the Ethics Committee of Nanjing Drum Tower Hospital (no. 2024AE01018). The protocol for obtaining human liver samples for immunohistochemical, qPCR, and western blotting was approved by the Ethics Committee of Nanjing Drum Tower Hospital (no. 2024‐482‐02). All patients and healthy liver donors provided written informed consent.

## Conflicts of Interest

The authors declare no conflict of interest.

## Author Contributions

Jie Li, Qianwen Zhao, Xia Lu, and Wei An conceived the project. Qianwen Zhao, Chao Wu, and Junping Shi designed the experiments. Qianqian Chen, Fajuan Rui, Zhiwen Fan, Hongju Yang, Nan Geng, Chenqi Lu, Wenjing Ni, Yue Huan, and Shengxia Yin performed experiments, collected data, and analyzed data. Qianwen Zhao and Qianqian Chen drafted the manuscript. All authors edited and finalized the manuscript. Jie Li, Qianwen Zhao and Hongju Yang secured funding and provided supervision.

## Supporting information




**Supporting File 1**: advs73472‐sup‐0001‐SuppMat.doc.

**Supporting File 2**: advs73472‐sup‐0002‐DataFile.docx.

## Data Availability

All study data are fully presented in the main article and accompanying appendix. Individual participant data will not be made available to others, as per ethical committee agreements. For further information, please contact the lead contact, Jie Li (lijier@nju.edu.cn). This paper does not report the original code.
