# Supplementary material for: Nuclear Factor I‐B Delays Liver Fibrosis by Inhibiting Chemokine Ligand 5 Transcription
Source: Adv Sci (Weinh). 2025 Dec 23;13(13):e11311. doi: 10.1002/advs.202511311 (PMC12955860; doi:10.1002/advs.202511311)
Supplement: Supplementary file 1 — Supporting File 1: advs73472‐sup‐0001‐SuppMat.doc. [file ADVS-13-e11311-s002.doc]

**Supplementary material**

**Nuclear Factor I-B Delays Liver Fibrosis by Inhibiting Chemokine Ligand 5 Transcription**

Qianqian Chen ^1,2,3,4#^, Fajuan Rui ^1,2,3#^, Zhiwen Fan ^5#^, Hongju Yang ^6#^, Nan Geng ^2,3^, Chenqi Lu ^2,3^, Wenjing Ni ^1,2,3^, Yue Huan ^7^, Junping Shi ^8^, Chao Wu ^2,3^, Shengxia Yin ^2,3^, Wei An ^9*^, Xia Lu ^10*^, Qianwen Zhao ^1,2,3*^, Jie Li ^2,1,3*^

1. Department of Infectious Diseases, Nanjing Drum Tower Hospital Clinical College of Nanjing University of Chinese Medicine, Nanjing, Jiangsu, China.

1. Department of Infectious Disease, Nanjing Drum Tower Hospital, Affiliated Hospital of Medical School, Nanjing University, Nanjing, Jiangsu, China.
2. Institute of Viruses and Infectious Diseases, Nanjing University, Nanjing, Jiangsu, China.
3. Department of Gastroenterology, The Affiliated Huai'an Hospital of Xuzhou' Medical University and Huai'an Second People's Hospital, Huai’an, Jiangsu, China.
4. Department of Pathology, Nanjing Drum Tower Hospital, Affiliated Hospital of Medical School, Nanjing University, Nanjing, Jiangsu, China.
5. Division of geriatric Gastroenterology, The First Afliated Hospital of Kunming Medical University, Kunming, Yunnan, China.
6. Jiangsu Key Laboratory of Oral Diseases, Jiangsu Province Engineering Research Center of Stomatological Translational Medicine, Department of General Dentistry Affiliated Hospital of Stomatology Nanjing Medical University, Nanjing, Jiangsu, China.
7. Department of Infectious & Hepatology Diseases, The Affiliated Hospital of Hangzhou Normal University, Hangzhou, Zhejiang, China.
8. Department of Cell Biology, Capital Medical University and the Municipal Key Laboratory for Liver Protection and Regulation of Regeneration, Beijing, China.
9. Department of Cardiology, Shanghai Sixth People’s Hospital Affiliated to Shanghai Jiao Tong, University School of Medicine, Shanghai, China.

**Supplementary figures: 14**

**Supplementary tables: 4**

**Fig. S1**

**
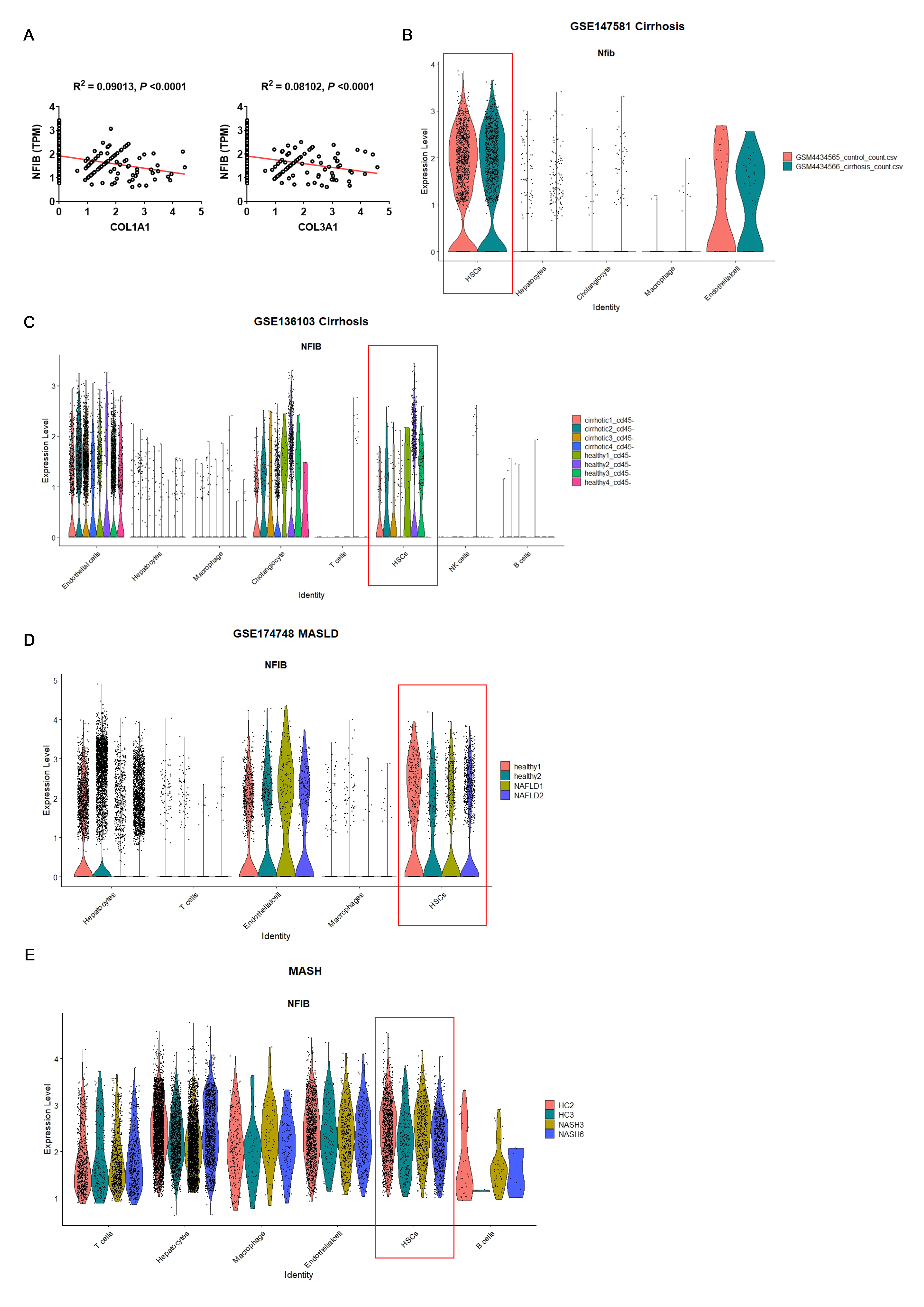
**

**Fig. S1**. (A) The NFIB level, Col1a1, and Col3a1 were re-analyzed based on the single cell sequencing of patients with cirrhosis (GSE136103), and the Pearson correlation was performed using GraphPad. (B-E) The level of NFIB was re-analyzed from single cell sequence of cirrhosis patients (GSE147581 and GSE136103) (B and C), and MASH patients (GSE174748, and our own MASH single-cell dataset) (D and E).

**Fig. S2**

**
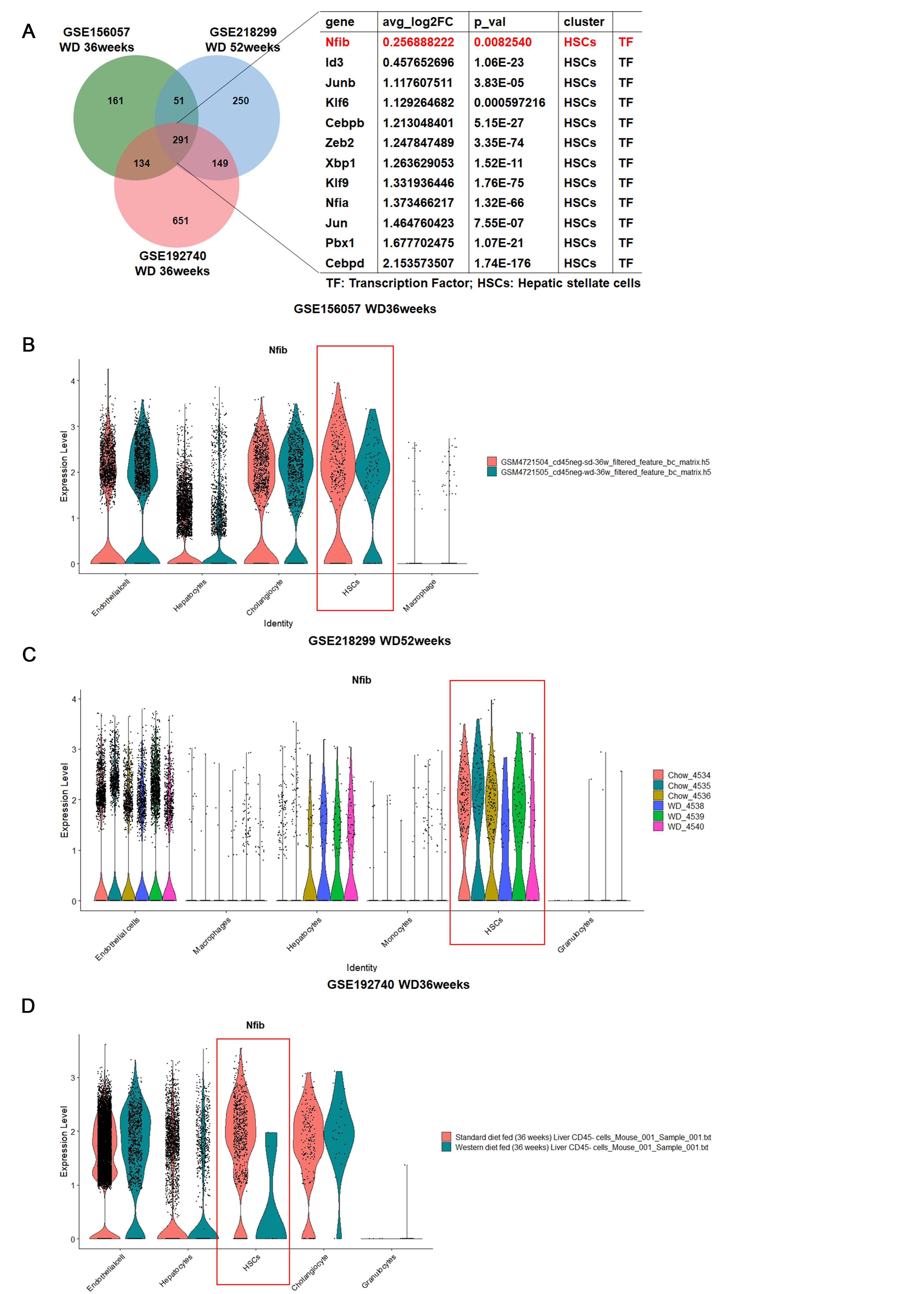
**

**Fig. S2**. (A) Wayne analysis of mouse single-cell sequencing datasets under MASH condition (GSE156057, GSE192740, and GSE218299). (B-D) The level of NFIB was re-analyzed from mouse single cell sequence of MASH condition (GSE156057, GSE192740, and GSE218299).

**Fig. S3**

**
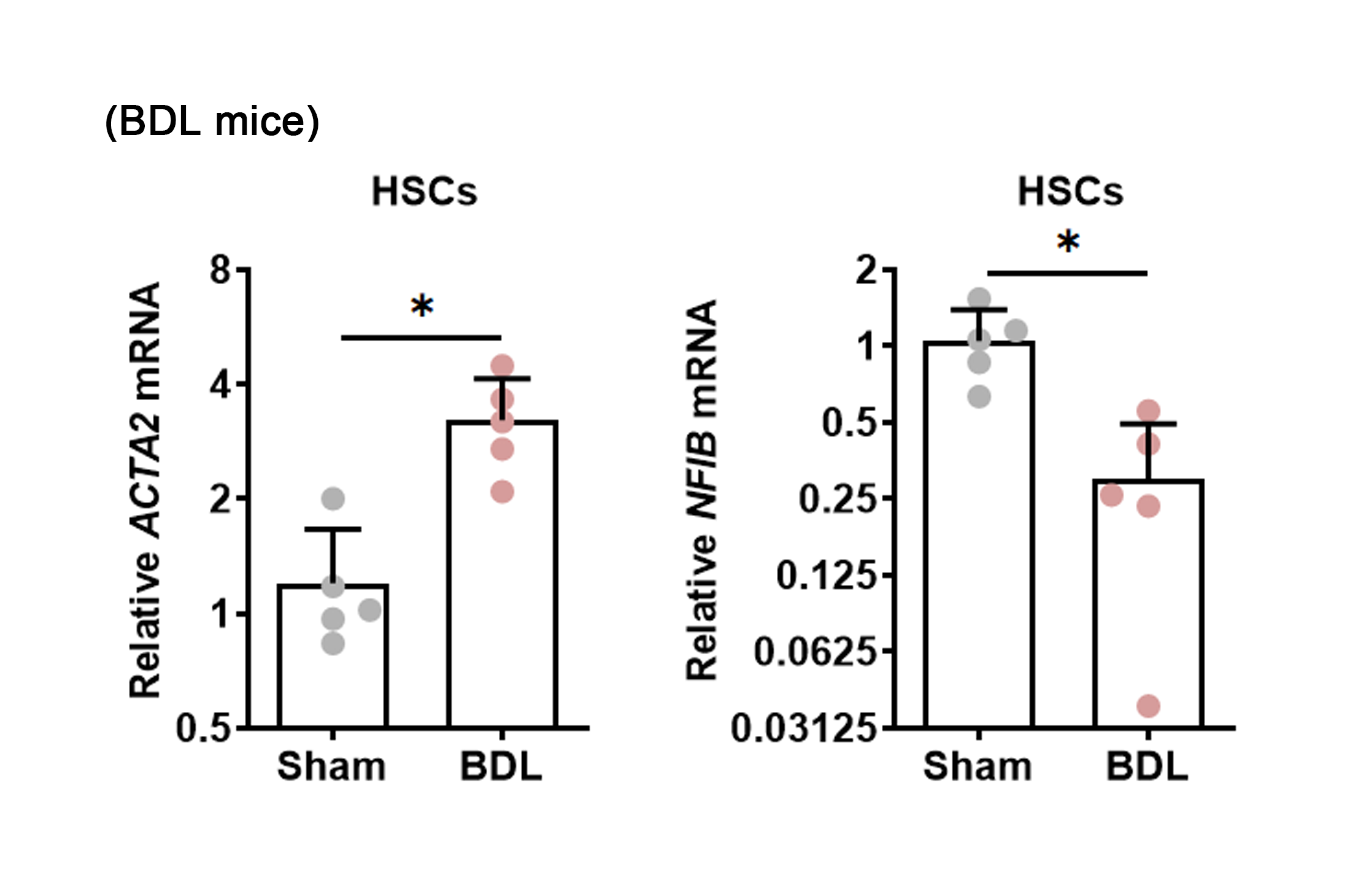
**

**Fig. S3**. Primary HSCs were isolated from mice with bile duct ligation (BDL)-induced liver fibrosis. The cells were harvested after 2 weeks and NFIB, ACTA2 mRNA were examined. N=5. Data are mean±SD. *p<0.05 by two-tailed t-test.

**Fig. S4**

**
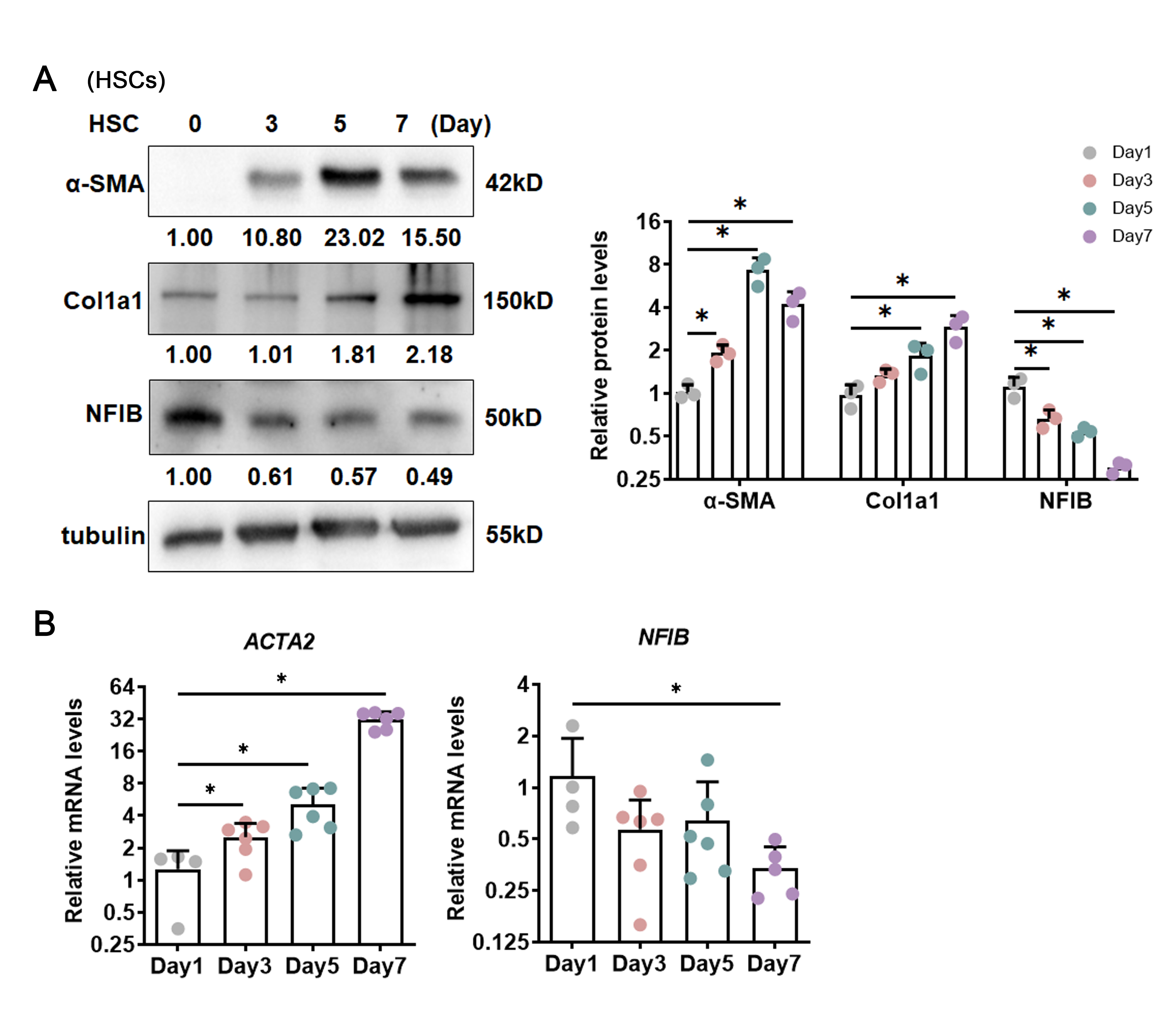
**

**Fig. S4**. Primary HSCs were isolated from C57BL/6 mice. The cells were harvested after 1, 3, 5, 7 days. (A) The protein expression of NFIB, α-SMA and Col1a1 were examined by western blotting. N=3. (B) The mRNA levels of ACTA2 and NFIB were examined by qPCR. N=4-6. Data are mean ± SD. *p<0.05 by two-tailed t-test.

**Fig. S5**

**
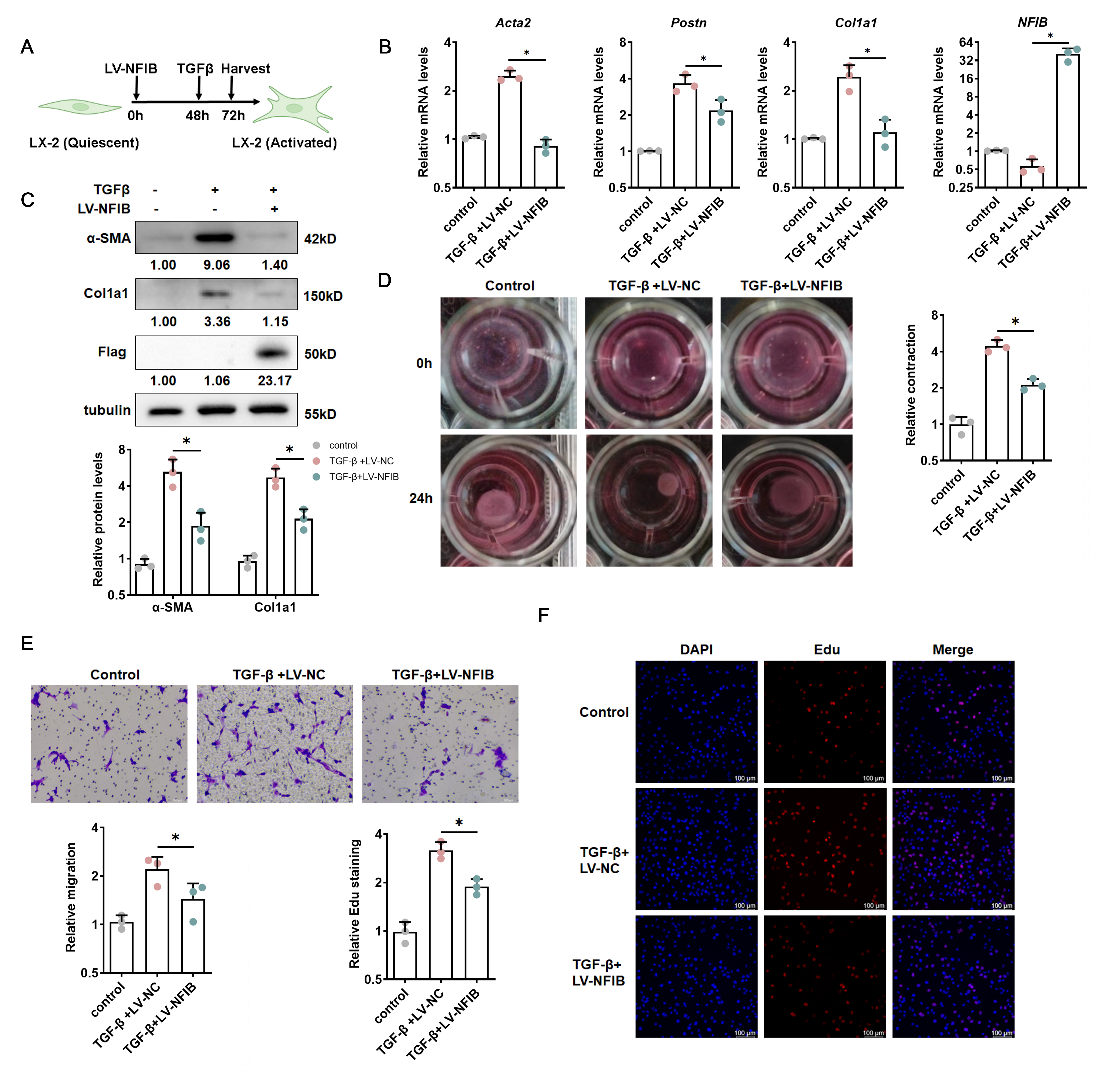
**

**Fig. S5**. LX-2 cells were transfected with overexpressed NFIB (LV-NFIB) and control virus (LV-NC) followed by treatment with TGF-β (2ng/ml) for 24h. (A) Scheme of cell protocol *in vitro*. (B, C) Myofibroblast marker genes were examined by qPCR and Western blotting. (D) Collagen contraction assay. (E) Boyden chamber transwell assay (100x). (F) Cell proliferation was evaluated by EdU incorporation (200x). N = 3 biological replicates. Data are as mean ± SD. *p<0.05 by two-tailed t-test.

**Fig. S6**


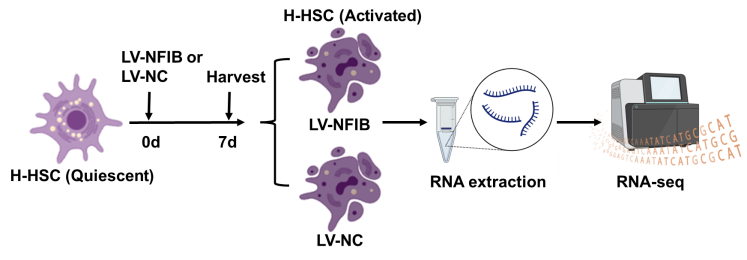


**Fig. S6**: Workflow for the single cell sequence of HSCs overexpressing NFIB.

**Fig. S7**

**
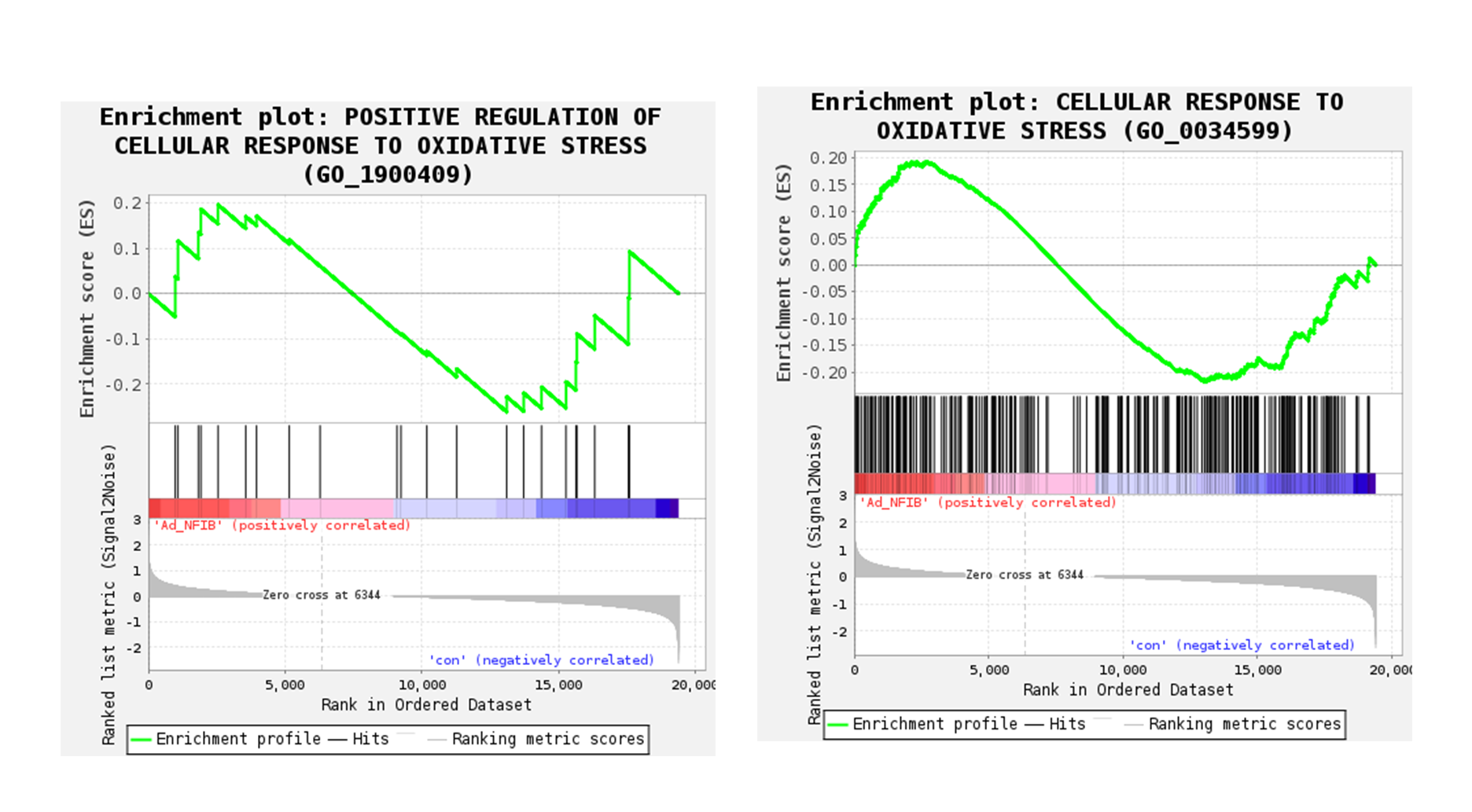
**

**Fig. S7**. Primary murine HSCs were transfected with overexpressed NFIB (LV-NFIB) and control virus (LV-NC). RNA-seq was performed and Gene set enrichment analysis (GSEA) was detected.

**Fig. S8**

**
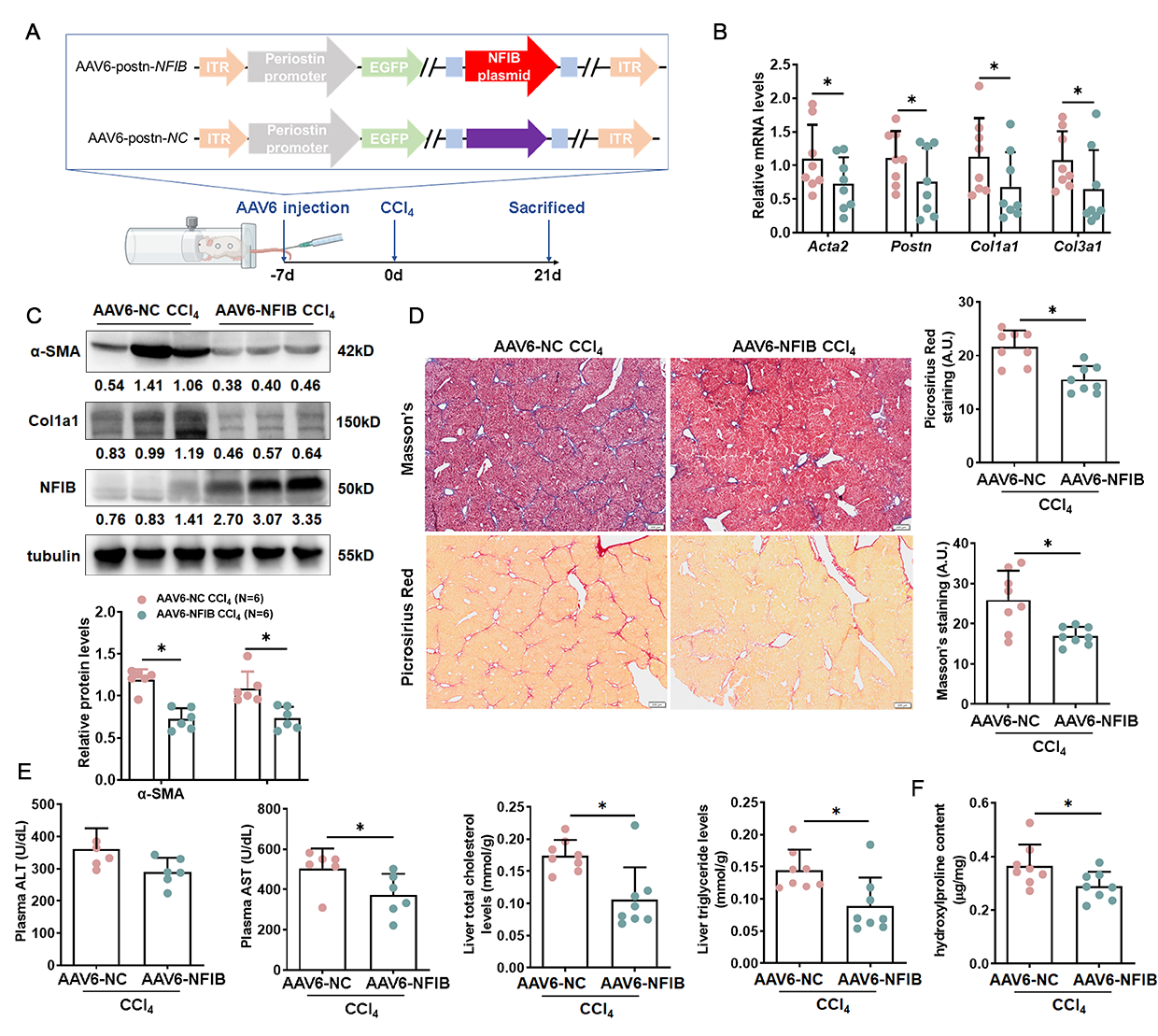
**

**Fig. S8**. C57BL/6 mice were injected with lentivirus carrying the overexpression plasmid targeting NFIB (AAV6-NFIB) or control plasmid (AAV6-NC) followed by injection with CCl_4_ for 3 weeks. (A) Schematic of the animal protocol. The expression of **profibrogenic markers in the liver** was assessed by qPCR (B) and Western blotting (C). (D) Paraffin sections were stained with picrosirius red (100x) and Masson’s trichrome (100x). (E) Plasma AST, ALT and liver total cholesterol, triglyceride level. (F) Hepatic hydroxyproline levels. N = 6-8. Data are mean±SD. *p<0.05 by two-tailed t-test.

**Fig. S9**

**
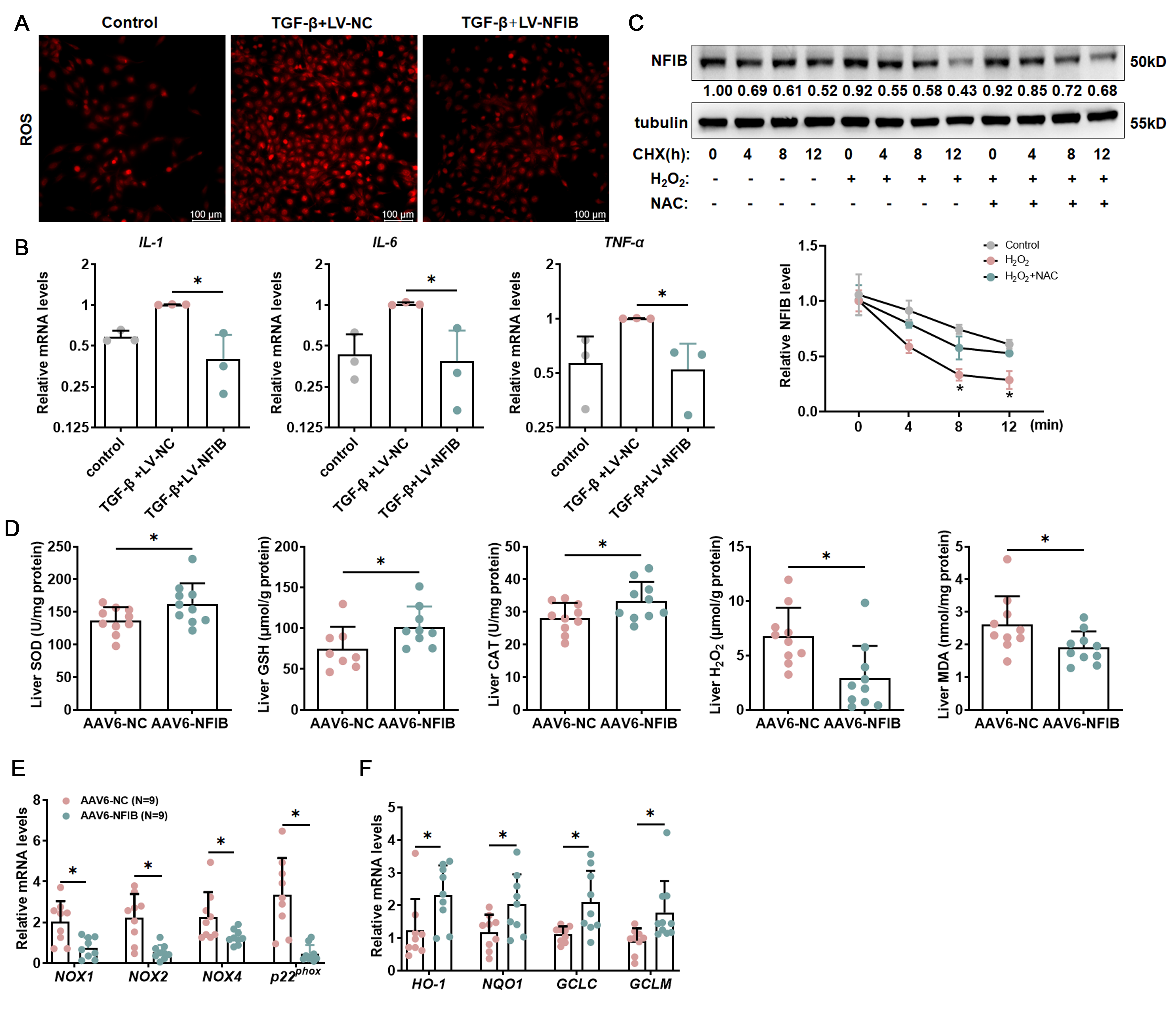
**

**Fig. S9**. LX-2 cells were transfected with overexpressed NFIB (lentivirus containing the NFIB [LV-NFIB]) or control virus (LV-NC). (A) DCF-DA staining (200×) for ROS production in LX-2 cell. (B) Inflammatory marker genes were examined using qPCR. N=3. (C) Western blotting analysis of extracts from LX-2 cells stimulated with H_2_O_2_ for 4 hours and then treated various times with cycloheximide (CHX; 100 μg) with or without N-acetyl-L-cysteine (NAC; 5 mM). N=3. (D) Examination for SOD, GSH, CAT, H_2_O_2_ and MDA in liver tissues of CDAHFD-induced mice. N=8-10. (E) Measurement of NOX1, NOX2, NOX4, and p22^phox^ gene expression by qPCR in liver samples of CDAHFD-induced mice. N=8-10. (F) Measurement of HO-1, NQO1, GCLC and GCLM gene expression by qPCR in liver tissues of CDAHFD-induced mice. N=8-10 . Data are mean±SD. *p<0.05 by two-tailed t-test.

**Fig. S10**

**Fig. S10**. C57BL/6 mice were injected with lentivirus carrying overexpression plasmid targeting NFIB (AAV6-NFIB) or control plasmid (AAV6-NC) followed by injection with CCl4 for 3 weeks. Examination for (A) SOD, (B) GSH, (C) CAT, (D) H_2_O_2_, (E) MDA in liver tissues of all groups of mice. (F) Measurement of NOX1, NOX2, NOX4 and p22^phox^ gene expression by qPCR in liver samples. (G) RT-qPCR analysis for antioxidants including HO-1, NQO1, GCLC and GCLM in liver tissues. (H) IF staining for 4-HNE expression in hepatic sections. (I) RT-qPCR results for inflammation markers IL-1, IL-6 and TNF-α gene expression levels in liver samples. (J) Immunohistochemical stain detected NLRP3, CD3, F4/80 and Ly6G level in all groups. N=6-8. Data are mean ± SD. *p<0.05 by two-tailed t-test.

**Fig. S11**

**
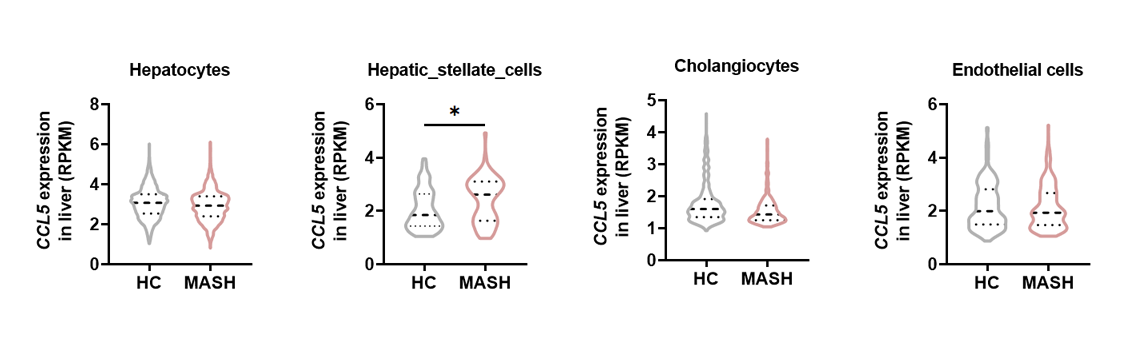
**

**Fig. S11.** MASH single cell analysis the CCL5 expression in hepatocytes, HSCs, cholangiocytes and endothelial cells. Data are mean±SD. *p<0.05 by two-tailed t-test.

**Fig.S12**


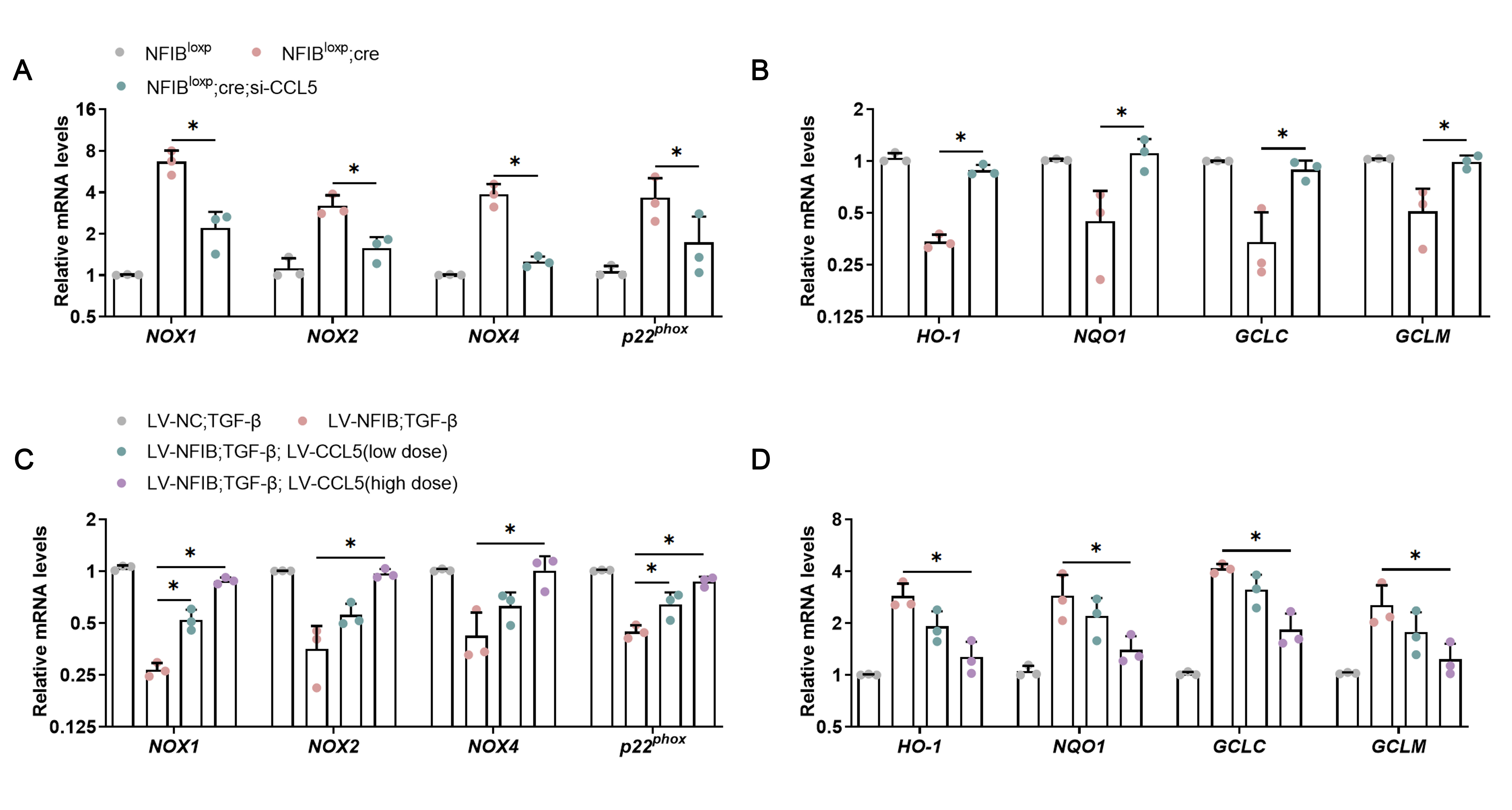


**Fig. S12.** (A and B) Primary murine HSCs were isolated from NFIB LoxP mice. The cells were treated with lentivirus carrying CRE for 72 hours and then transfected with siRNA against CCL5. (A) NOX1, NOX2, NOX4 and p22phox mRNA levels were examined by qPCR. N = 3. (B) HO-1, NQO1, GCLC and GCLM mRNA levels by qPCR. N = 3. (C and D) LX-2 cells were transfected with NFIB-overexpressing lentivirus (LV-NFIB) and were subsequently treated with low- (4x10^5^ TU) or high-dose (8x10^5^ TU) CCL5-overexpressing lentivirus (LV-CCL5). (C) NOX1, NOX2, NOX4 and p22^phox^ mRNA levels by qPCR. N = 3. (D) HO-1, NQO1, GCLC and GCLM mRNA levels were examined by qPCR. N = 3. Data are mean±SD. *p<0.05 by two-tailed t-test.

**Fig. S13**

**
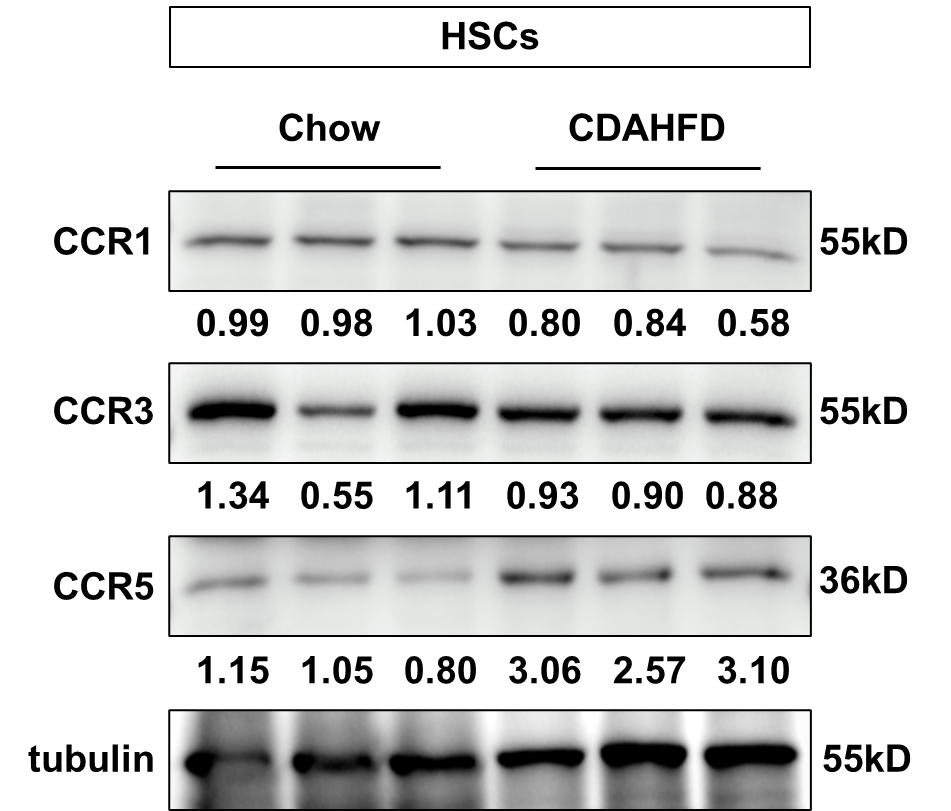
**

**Fig. S13.** Mice were fed the CDAHFD diet for 8 weeks, and CCR1, CCR2, and CCR5 protein levels from isolated HSCs were measured by Western blotting. N=3.

**Fig. S14**


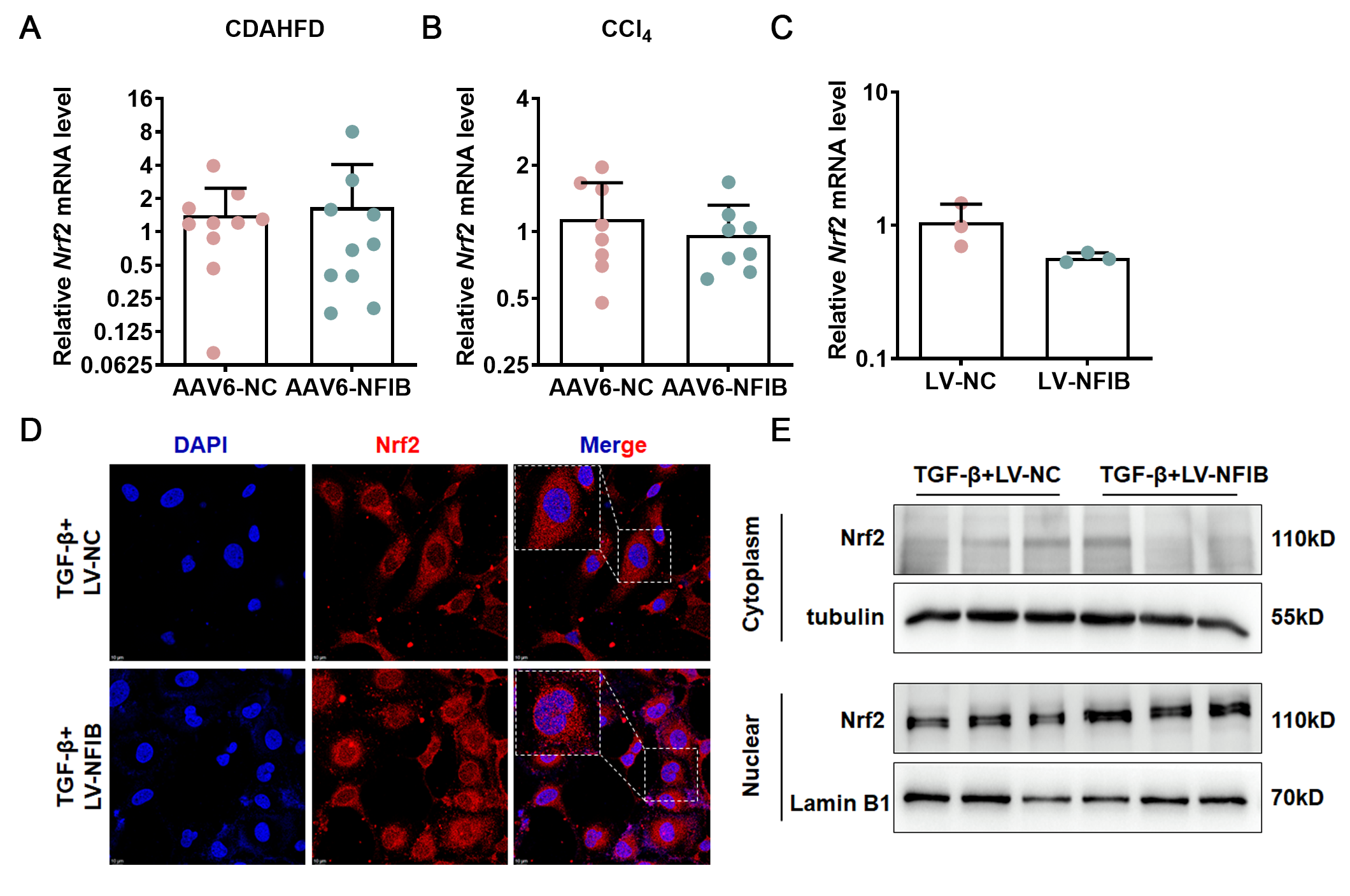


**Fig. S14.** *Nrf2* mRNA levels were measured by qPCR in CDAHFD-(A) and CCl_4_-induced(B) fibrosis mice, and primary human HSCs (C). (D and E) LX-2 cells were transfected with NFIB-overexpressing lentivirus (LV-NFIB) or control virus (LV-NC) followed by treatment with TGF-β (2ng/ml) for 24h. (D) IF staining for Nrf2 expression in LX-2 cells. (E) The protein level of Nrf2 in Nuclear and Cytoplasm. N=3.

**Supplementary tables**

**Supplementary table 1. Information of participants for tissue analysis**

| **ID** | **Age (years)** | **Sex** | **Scheuer score** | **Etiology** | **ALT** | **AST** |
| --- | --- | --- | --- | --- | --- | --- |
| 1 | 44 | M | 0 | DILI | 124.4 | 60.9 |
| 2 | 19 | M | 0 | MASLD | 471.0 | 277.7 |
| 3 | 46 | M | 0 | MASLD | 62.8 | 240.5 |
| 4 | 40 | M | 0 | MASLD | 268.0 | 158.0 |
| 5 | 40 | M | 0 | MASLD | 39.5 | 28.6 |
| 6 | 34 | F | 0 | MASLD | 83.2 | 63.7 |
| 7 | 51 | F | 0 | MASLD | 32.2 | 24.4 |
| 8 | 31 | F | 0 | MASLD | 26.7 | 16.2 |
| 9 | 32 | M | 0 | MASLD | 60.7 | 26.2 |
| 10 | 38 | F | 0 | MASLD | 29.5 | 18.4 |
| 11 | 29 | F | 0 | Unexplained liver disease | 46.4 | 26.3 |
| 12 | 39 | M | 0 | MASLD | 105.5 | 50.4 |
| 13 | 34 | F | 0 | CHB | 12.4 | 22.5 |
| 14 | 35 | F | 0 | MASLD | 29.5 | 18.4 |
| 15 | 29 | F | 0 | MASLD | 18.2 | 13.2 |
| 16 | 23 | F | 0 | MASLD | 19.0 | 13.2 |
| 17 | 41 | M | 0 | MASLD | 75.6 | 49.1 |
| 18 | 24 | F | 0 | MASLD | 63.6 | 33.9 |
| 19 | 32 | M | 0 | MASLD | 101.0 | 58.2 |
| 20 | 50 | F | 0 | MASLD | 60.1 | 31.4 |
| 21 | 63 | F | 0 | MASLD | 33.3 | 29.3 |
| 22 | 35 | F | 0 | MASLD | 30.1 | 21.7 |
| 23 | 24 | M | 0 | MASLD | 32.6 | 20.5 |
| 24 | 30 | F | 0 | MASLD | 49.9 | 21.3 |
| 25 | 32 | F | 0 | MASLD | 59.4 | 38.4 |
| 26 | 37 | F | 0 | MASLD | 81.8 | 36.1 |
| 27 | 32 | F | 0 | MASLD | 31.2 | 25.1 |
| 28 | 48 | F | 0 | MASLD | 10.8 | 13.9 |
| 29 | 28 | F | 0 | MASLD | 26.7 | 16.2 |
| 30 | 15 | M | 0 | MASLD | 22.7 | 17.9 |
| 31 | 18 | M | 0 | MASLD | 63.1 | 31.1 |
| 32 | 30 | M | 0 | MASLD | 60.7 | 26.2 |
| 33 | 15 | F | 0 | MASLD | 89.9 | 45.5 |
| 34 | 24 | F | 0 | MASLD | 28.1 | 17.6 |
| 35 | 40 | M | 0 | MASLD | 33.7 | 27.6 |
| 36 | 25 | F | 0 | MASLD | 32.4 | 31.0 |
| 37 | 33 | M | 0 | MASLD | 53.7 | 32.5 |
| 38 | 32 | F | 0 | MASLD | 54.0 | 45.7 |
| 39 | 28 | M | 0 | MASLD | 42.3 | 18.9 |
| 40 | 32 | F | 0 | MASLD | 29.1 | 19.8 |
| 41 | 37 | F | 0 | MASLD | 30.1 | 22.6 |
| 42 | 36 | F | 0 | MASLD | 31.6 | 24.3 |
| 43 | 27 | F | 0 | MASLD | 37.4 | 26.9 |
| 44 | 31 | F | 0 | MASLD | 39.9 | 35.6 |
| 45 | 35 | F | 0 | MASLD | 17.0 | 11.7 |
| 46 | 21 | F | 0 | MASLD | 13.6 | 10.8 |
| 47 | 34 | M | 0 | MASLD | 19.4 | 15.8 |
| 48 | 23 | F | 0 | MASLD | 61.3 | 28.6 |
| 49 | 31 | M | 0 | MASLD | 102.5 | 53.0 |
| 50 | 30 | F | 0 | MASLD | 112.4 | 60.6 |
| 51 | 25 | M | 0 | MASLD | 72.6 | 37.1 |
| 52 | 37 | F | 0 | MASLD | 80.6 | 50.7 |
| 53 | 33 | F | 1 | MASLD | 178.1 | 78.6 |
| 54 | 41 | M | 1 | MASLD | 70.0 | 32.0 |
| 55 | 50 | M | 1 | MASLD | 170.0 | 143.0 |
| 56 | 37 | M | 1 | MASLD | 248.8 | 124.4 |
| 57 | 28 | M | 1 | MASLD | 132.9 | 78.7 |
| 58 | 31 | M | 1 | MASLD | 154.9 | 54.6 |
| 59 | 36 | M | 1 | MASLD | 56.3 | 36.2 |
| 60 | 42 | F | 1 | MASLD | 237.0 | 133.0 |
| 61 | 18 | M | 1 | MASLD | 52.0 | 23.5 |
| 62 | 29 | M | 1 | MASLD | 41.2 | 24.3 |
| 63 | 34 | M | 1 | MASLD | 52.8 | 34.1 |
| 64 | 39 | M | 1 | MASLD | 50.3 | 33.0 |
| 65 | 35 | M | 1 | MASLD | 13.6 | 32.1 |
| 66 | 29 | M | 1 | MASLD | 12.9 | 86.2 |
| 67 | 35 | F | 1 | MASLD | 10.3 | 22.7 |
| 68 | 30 | M | 1 | MASLD | 18.1 | 116.4 |
| 69 | 21 | F | 1 | MASLD | 13.5 | 74.3 |
| 70 | 38 | F | 1 | MASLD | 12.2 | 10.5 |
| 71 | 34 | F | 1 | MASLD | 21.2 | 15.0 |
| 72 | 32 | F | 1 | MASLD | 8.7 | 12.2 |
| 73 | 36 | F | 1 | MASLD | 64.6 | 98.4 |
| 74 | 37 | F | 1 | MASLD | 79.4 | 43.2 |
| 75 | 32 | M | 1 | MASLD | 135.9 | 77.0 |
| 76 | 57 | F | 1 | MASLD | 25.2 | 24.9 |
| 77 | 36 | F | 1 | MASLD | 17.0 | 16.2 |
| 78 | 28 | F | 1 | MASLD | 19.6 | 27.4 |
| 79 | 28 | F | 1 | MASLD | 58.7 | 20.3 |
| 80 | 51 | F | 1 | MASLD | 17.5 | 22.7 |
| 81 | 38 | F | 1 | MASLD | 12.2 | 10.5 |
| 82 | 34 | F | 1 | MASLD | 21.2 | 15.0 |
| 83 | 40 | M | 1 | MASLD | 41.5 | 23.1 |
| 84 | 62 | F | 1 | MASLD | 15.9 | 19.7 |
| 85 | 33 | F | 1 | MASLD | 26.4 | 20.3 |
| 86 | 30 | F | 1 | MASLD | 112.4 | 60.6 |
| 87 | 37 | M | 1 | MASLD | 80.2 | 29.6 |
| 88 | 18 | M | 2 | MASLD | 151.1 | 58.4 |
| 89 | 47 | M | 2 | MASLD | 41.7 | 29.7 |
| 90 | 52 | F | 2 | MASLD | 148.5 | 44.1 |
| 91 | 53 | F | 2 | MASLD | 89.8 | 67.5 |
| 92 | 24 | M | 2 | MASLD | 576.0 | 339.0 |
| 93 | 29 | M | 2 | MASLD | 17.0 | 19.0 |
| 94 | 26 | M | 2 | MASLD | 38.4 | 17.8 |
| 95 | 37 | M | 2 | MASLD | 21.5 | 15.9 |
| 96 | 30 | M | 2 | CHB | 305.4 | 113.9 |
| 97 | 58 | F | 2 | Unexplained liver disease | 28.9 | 48.4 |
| 98 | 24 | F | 2 | MASLD | 26.0 | 15.6 |
| 99 | 27 | M | 2 | MASLD | 34.5 | 25.8 |
| 100 | 31 | M | 2 | MASLD | 62.3 | 39.8 |
| 101 | 12 | M | 2 | MASLD | 101.1 | 61.4 |
| 102 | 26 | F | 2 | MASLD | 18.6 | 11.8 |
| 103 | 35 | M | 2 | MASLD | 33.3 | 20.0 |
| 104 | 40 | M | 2 | MASLD | 64.4 | 84.8 |
| 105 | 19 | F | 2 | MASLD | 14.4 | 11.7 |
| 106 | 36 | M | 2 | MASLD | 41.9 | 21.7 |
| 107 | 31 | M | 2 | MASLD | 47.4 | 24.2 |
| 108 | 33 | F | 2 | MASLD | 40.1 | 33.0 |
| 109 | 26 | F | 2 | MASLD | 29.1 | 27.3 |
| 110 | 37 | F | 2 | MASLD | 35.6 | 24.0 |
| 111 | 26 | M | 3 | MASLD | 253.4 | 114.7 |
| 112 | 53 | M | 3 | MASLD | 94.4 | 40.4 |
| 113 | 24 | F | 3 | MASLD | 139.4 | 74.9 |
| 114 | 23 | F | 3 | MASLD | 12.9 | 12.5 |
| 115 | 28 | M | 3 | MASLD | 75.0 | 59.1 |
| 116 | 44 | F | 3 | Unexplained liver disease | 19.4 | 20.4 |
| 117 | 36 | F | 3 | CHB | 7.7 | 69.7 |
| 118 | 31 | M | 3 | CHB | 53.0 | 39.0 |
| 119 | 53 | M | 3 | CHB | 73.2 | 83.6 |
| 120 | 31 | M | 3 | CHC | 90.9 | 92.0 |
| 121 | 50 | F | 3 | CHF | 72.7 | 41.7 |
| 122 | 60 | M | 4 | CHB | 13.6 | 20.6 |
| 123 | 59 | F | 4 | MASLD | 39.2 | 45.5 |
| 124 | 33 | M | 4 | ALD | 60.8 | 40.2 |
| 125 | 44 | F | 4 | DILI | 40.2 | 134.2 |
| 126 | 38 | F | 4 | MASLD | 29.8 | 21.9 |
| 127 | 37 | M | 4 | CHB | 41.6 | 37.1 |
| 128 | 40 | M | 4 | ALD | 27.0 | 23.5 |
| 129 | 47 | F | 4 | CHB | 10.9 | 30.8 |
| 130 | 43 | F | 4 | AIH | 326.0 | 628.0 |
| 131 | 58 | F | 4 | AIH | 132.0 | 230.0 |
| 132 | 41 | F | 4 | CHB | 12.7 | 28.9 |
| 133 | 51 | M | 4 | Unexplained liver disease | 13.4 | 21.2 |
| 134 | 50 | M | 4 | CHB | 20.9 | 30.6 |
| 135 | 56 | M | 4 | CHB | 23.2 | 20.7 |
| 136 | 51 | M | 4 | CHB | 66.5 | 84.3 |
| 137 | 32 | M | 4 | CHB | 11.4 | 13.7 |
| 138 | 50 | M | 4 | CHB | 11.3 | 19.5 |
| 139 | 50 | M | 4 | AIH | 48.0 | 82.0 |
| 140 | 49 | M | 4 | CHB | 40.2 | 53.7 |
| 141 | 56 | F | 4 | CHC | 18.7 | 36.1 |
| 142 | 52 | M | 4 | CHB | 15.0 | 37.4 |
| 143 | 55 | M | 4 | CHB | 69.8 | 26.5 |
| 144 | 54 | M | 4 | ALD | 31.0 | 58.0 |
| 145 | 40 | M | 4 | CHB | 16.5 | 19.6 |
| 146 | 53 | M | 4 | CHB | 48.1 | 33.1 |
| 147 | 60 | M | 4 | CHB | 25.7 | 32.7 |
| 148 | 30 | M | 4 | CHB | 15.0 | 11.0 |
| 149 | 52 | F | 4 | CHB | 19.9 | 22.9 |
| 150 | 38 | M | 4 | ALD | 38.0 | 43.3 |
| 151 | 37 | F | 4 | PSC | 27.5 | 24.2 |
| 152 | 53 | M | 4 | CHB | 25.4 | 46.9 |
| 153 | 37 | M | 4 | CHB | 19.7 | 11.3 |
| 154 | 43 | M | 4 | CHB | 12.2 | 17.5 |
| 155 | 72 | F | 4 | AIH | 7.0 | 16.4 |
| 156 | 52 | M | 4 | CHB | 43.9 | 123.5 |
| 157 | 51 | F | 4 | CHB | 42.9 | 48.1 |
| 158 | 40 | F | 4 | AIH | 30.0 | 41.0 |

Note: M, male; F, female; ALT, alanine aminotransferase; AST, aspartate aminotransferase; DILI, drug-induced liver injury; MASLD, metabolic dysfunction-associated steatotic liver disease; CHB, chronic hepatitis B; CHF, congenital hepatic fibrosis; ALD, Alcohol-associated liver disease; AIH, autoimmune hepatitis; PSC, primary sclerosing cholangitis; CHC, chronic hepatitis C.

**Supplementary table 2. Primer sequences used for RT-qPCR analysis**

| **Items** | **Primer sequences (5’→3’)** |
| --- | --- |
| NFIB (Homo sapiens) | Forward: GAGAGCGGCTCATGAAATCC |
|  | Reverse: CCAGAATCTTGCTCCTGCAC |
| Acta2 (Homo sapiens) | Forward: GGGACGACATGGAAAAGATCTG |
|  | Reverse: GCAGGGTGGGATGCTCTTC |
| POSTN (Homo sapiens) | Forward: CTCATAGTCGTATCAGGGGTCG |
|  | Reverse:ACACAGTCGTTTTCTGTCCAC |
| COL1A1 (Homo sapiens) | Forward: GTGCTCCTGGTATTGCTGGT |
|  | Reverse: TCCTTGAACACCAACAGGGC |
| COL3A1 (Homo sapiens) | Forward:TTCGACTTCTCTCCAGCCGA |
|  | Reverse: TCCACTGGCCTGATCCATGT |
| NLRP3 (Homo sapiens) | Forward: AGGAAAAGGAAGGCCGACAC |
|  | Reverse: TGGAAGTGAGGTGGCTGTTC |
| HO-1 (Homo sapiens) | Forward: GAGAATGCTGAGTTCATG |
|  | Reverse: ATGTTGAGCAGGAAGGC |
| NQO1 (Homo sapiens) | Forward: ACGCCCGAATTCAAATCCT |
|  | Reverse: CCTGCCTGGAAGTTTAGGTC |
| GCLC (Homo sapiens) | Forward: TGTTTCCTGGACTGATCCCAA |
|  | Reverse: TGCGATAAACTCCCTCATCCAT |
| GCLM (Homo sapiens) | Forward: GCTTCTTGGAAACTTGCTTCA |
|  | Reverse: CTGTGTGATGCCACCAGATT |
| NOX1 (Homo sapiens) | Forward: CACAAGAAAAATCCTTGGGTCAA |
|  | Reverse: GACAGCAGATTGCGACACACA |
| NOX2 (Homo sapiens) | Forward: GGGTGATGTGGTGGATGTCG |
|  | Reverse: TGTCCTTTGAGTCAGGGCTC |
| NOX4 (Homo sapiens) | Forward: TGGCTGCCCATCTGGTGAATG |
|  | Reverse: CAGCAGCCCTCCTGAAACATGC |
| p22phox (Homo sapiens) | Forward: CGCTTCACCCAGTGGTACTT |
|  | Reverse: CAGCCGCCAGTAGGTAGATG |
| CCL5 (Homo sapiens) | Forward:CCAGCAGTCGTCTTTGTCAC |
|  | Reverse:CTCTGGGTTGGCACACACTT |
| NFIB (Mus musculus) | Forward: TGAGGCAGCTTCACCTACAG |
|  | Reverse:AGGATGGGTCTCTTGGGCTTA |
| IL-6 (Mus musculus) | Forward: TAGTCCTTCCTACCCCAATTTCC |
|  | Reverse: TTGGTCCTTAGCCACTCCTTC |
| TNF-α (Mus musculus) | Forward: CCCTCACACTCAGATCATCTTCT |
|  | Reverse: GCTACGACGTGGGCTACAG |
| IL-1β (Mus musculus) | Forward: GCAACTGTTCCTGAACTCAACT |
|  | Reverse: ATCTTTTGGGGTCCGTCAACT |
| CCL5 (Mus musculus) | Forward: TTTGCCTACCTCTCCCTCG |
|  | Reverse: CGACTGCAAGATTGGAGCACT |
| Acta2 (Mus musculus) | Forward: GTCCCAGACATCAGGGAGTAA |
|  | Reverse: TCGGATACTTCAGCGTCAGGA |
| POSTN (Mus musculus) | Forword: GAACGAATCATTACAGGTCC |
|  | Reverse: GGAGACCTCTTTTTGCAAGA |
| COL1A1 (Mus musculus) | Forward:GCTCCTCTTAGGGGCCACT |
|  | Reverse: CCACGTCTCACCATTGGGG |
| COL3A1 (Mus musculus) | Forward: CTGTAACATGGAAACTGGGGAAA |
|  | Reverse: CCATAGCTGAACTGAAAACCACC |
| HO-1 (Mus musculus) | Forward: CGTGCTCGAATGAACACTCT |
|  | Reverse: GGAAGCTGAGAGTGAGGACC |
| NQO1 (Mus musculus) | Forward: CAGCCAATCAGCGTTCGGTA |
|  | Reverse: TTGCTGTTGAGGTCGCAGGAG |
| GCLC (Mus musculus) | Forward: CCATCACTTCATTCCCCAGA |
|  | Reverse: GATGCCGGATGTTTCTTGTT |
| GCLM (Mus musculus) | Forward: ATGGAGTTCCCAAATCAGCC |
|  | Reverse: ATTGGGTTTTACCTGTGCCC |
| NOX1 (Mus musculus) | Forward: GGTCACTCCCTTTGCTTCCA |
|  | Reverse: GGCAAAGGCACCTGTCTCTCT |
| NOX2 (Mus musculus) | Forward: TTGGGTCAGCACTGGCTCTG |
|  | Reverse: TGGCGGTGTGCAGTGCTATC |
| NOX4 (Mus musculus) | Forward: TGCCTGCTCATTTGGCTGT |
|  | Reverse: CCGGCACATAGGTAAAAGGATG |
| p22phox (Mus musculus) | Forward: AACGAGCAGGCGCTGGCGTCCG |
|  | Reverse: CACAGTGGTATTTCGGCGCC |
| NLRP3 (Mus musculus) | Forward: CGAGACCTCTGGGAAAAAGCT |
|  | Reverse: GCATACCATAGAGGAATGTGATGTACA |
| 18S (Mus musculus) | Forword: CATTCGAACGTCTGCCCTATC |
|  | Reverse: CCTGCTGCCTTCCTTGGA |

**Supplementary table 3. Primary and secondary antibodies used in the study.**

| **Name** | **Catalog** | **Application** | **Dilutions** | **Vendors** |
| --- | --- | --- | --- | --- |
| NLRP3 | MA5-32255 | IHC | 1:200 | Thermo Fisher Scientific |
| α-SMA | ab124964 | WB  IF | 1:1000  1:200 | Abcam |
| 4-HNE | ab48506 | IF | 1:200 | Abcam |
| α-tubulin | ab7291 | WB | 1:5000 | Abcam |
| Alexa FluorTM 488 donkey anti-rabbit antibody | R37118 | IF | 1:400 | Thermo Fisher Scientific |
| Alexa FluorTM 594 donkey anti-mouse antibody | R37115 | IF | 1:400 | Thermo Fisher Scientific |
| NFIB | ab186738 | WB;  IF;  Co-IP | 1:1000;  1:200;  1:50 | Abcam |
| F4/80 | ab300421 | IHC | 1:5000 | Abcam |
| COL1A1 | ab260043 | WB;  IF | 1:1000;  1:200 | Abcam |
| CD3 | ab40763 | IHC | 1:200 | Abcam |
| Flag | ab205606 | WB | 1:1000 | Abcam |
| Ly6G | ab238132 | IHC | 1:200 | Abcam |
| IRF3 | ab68481 | WB;  Co-IP | 1:1000;  1:50 | Abcam |
| Smad2/3 | ab202445 | WB | 1:1000 | Abcam |
| p-Smad2 | ab280897 | WB | 1:1000 | Abcam |
| p-Smad3 | ab52903 | WB | 1:1000 | Abcam |
| P65 | ab32536 | WB | 1:1000 | Abcam |
| p-P65 | ab109458 | WB | 1:1000 | Abcam |
| CCR1 | A18341 | WB | 1:1000 | ABclonal |
| CCR3 | A01748-1 | WB | 1:1000 | BOSTER |
| CCR5 | 82942-1-RR | WB | 1:2000 | Proteintech |

**Supplementary table 4. Real-time ChIP-qPCR primers**

| **Gene** | **Forward primer** | **Reverse primer** |
| --- | --- | --- |
| CCL5 ~ primer1 | GAGACCTGTCCTCTGTGGTACGA | ACCTGGTCGCTTAGCACTTGGA |
| CCL5 ~ primer2 | GAGTGAGAGCCAGGTAGCATTTCTT | TCTGATGTCTCCAGGTCAATGTTCC |
