# Supplementary material for: Nuclear Factor I‐B Delays Liver Fibrosis by Inhibiting Chemokine Ligand 5 Transcription
Source: Adv Sci (Weinh). 2025 Dec 23;13(13):e11311. doi: 10.1002/advs.202511311 (PMC12955860; doi:10.1002/advs.202511311)

**Uncropped original western blots**

Figure···1


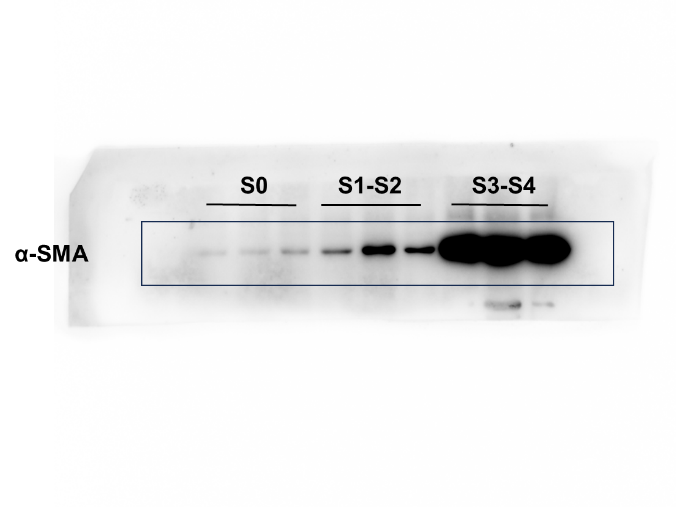

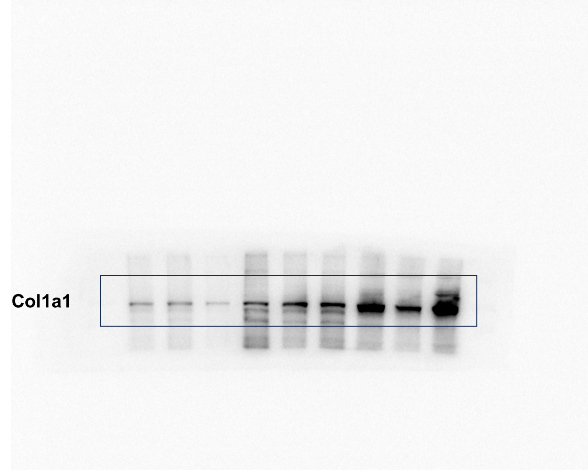


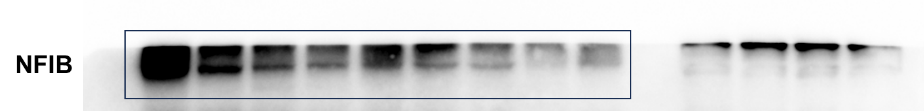


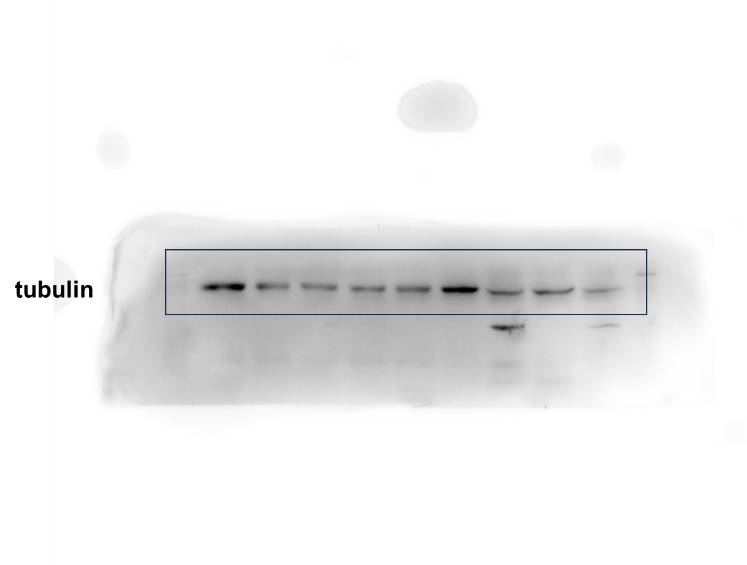


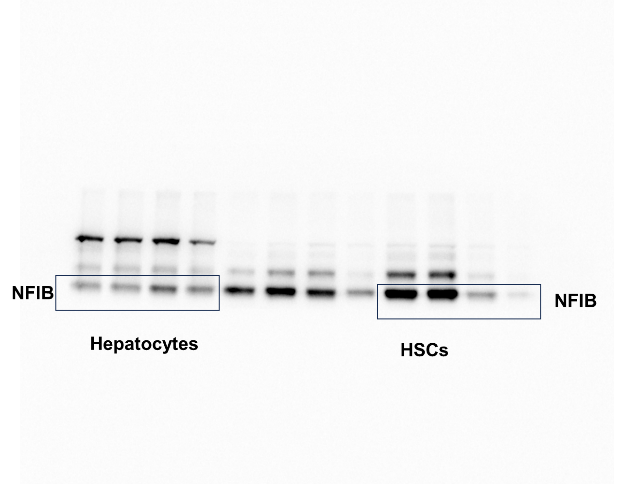

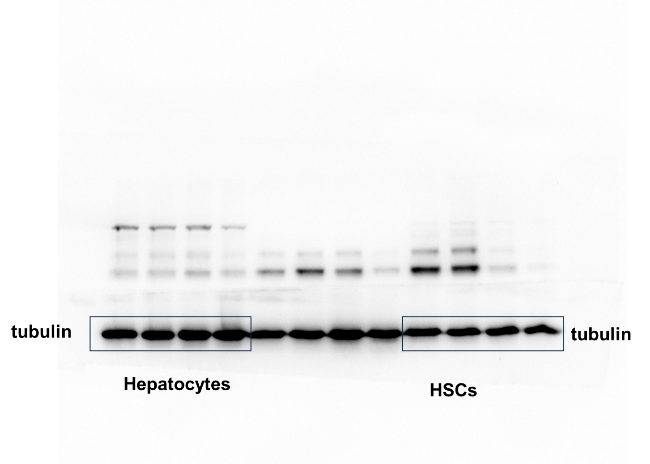


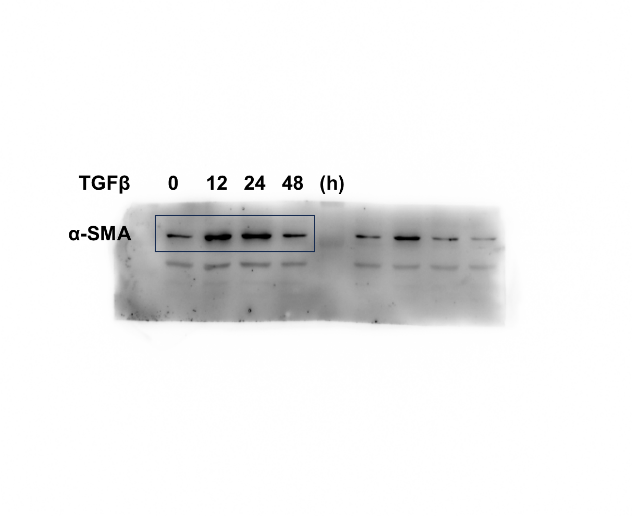

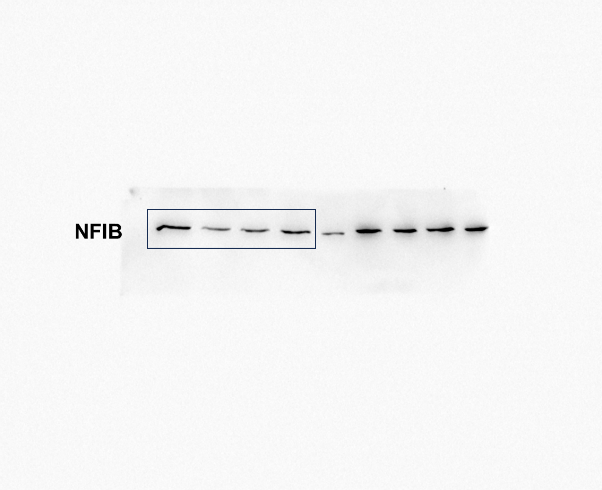


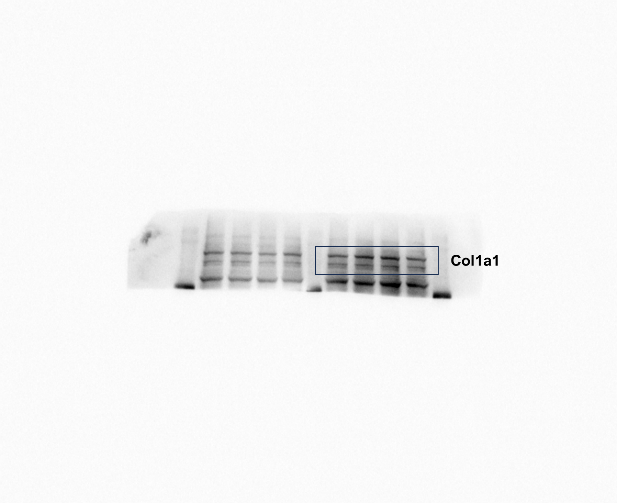

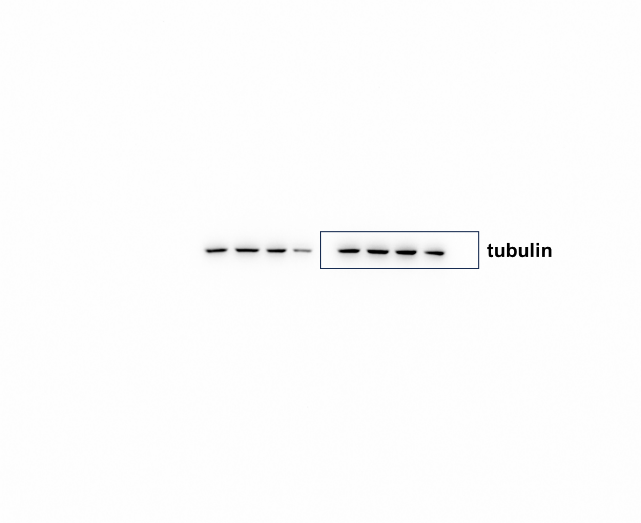


Figure···2


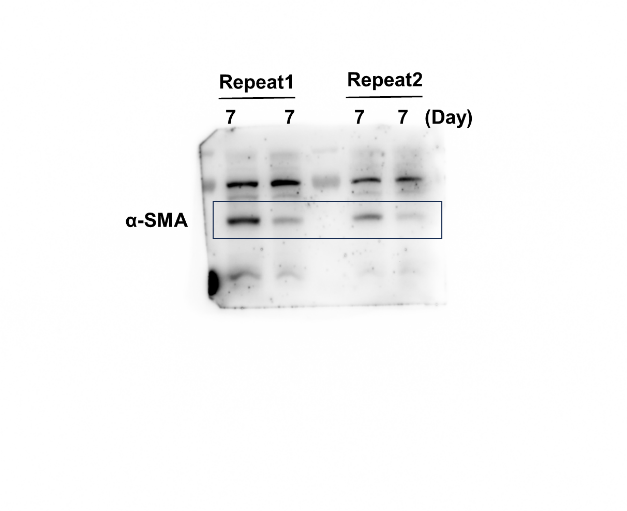

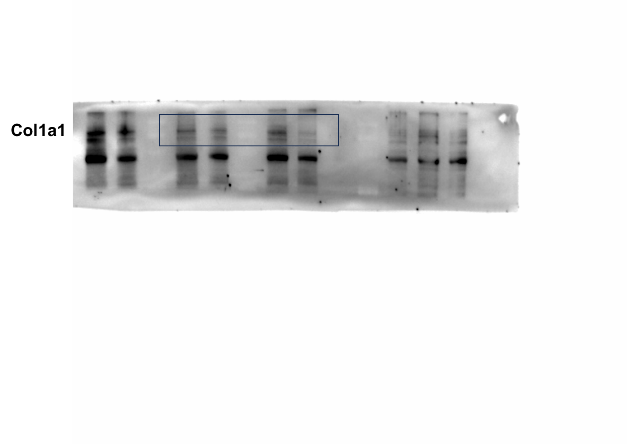


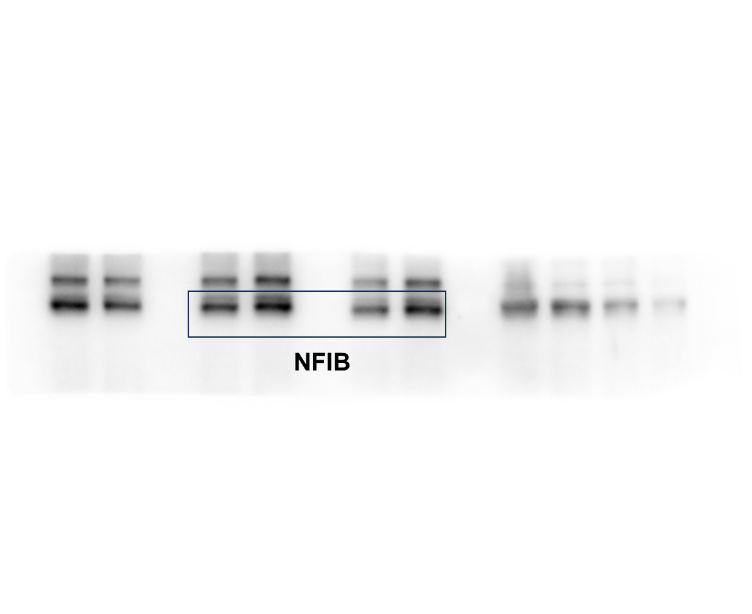

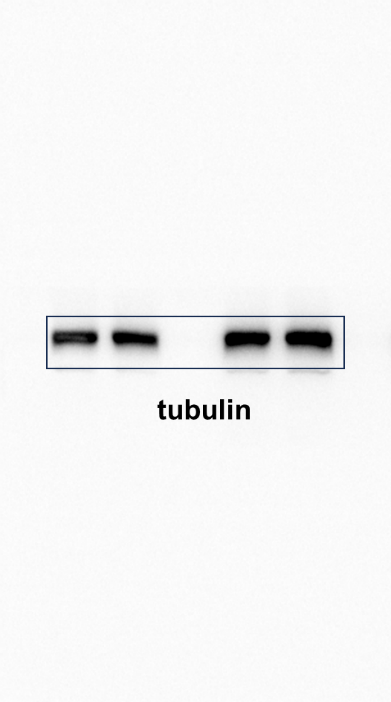


Figure···3


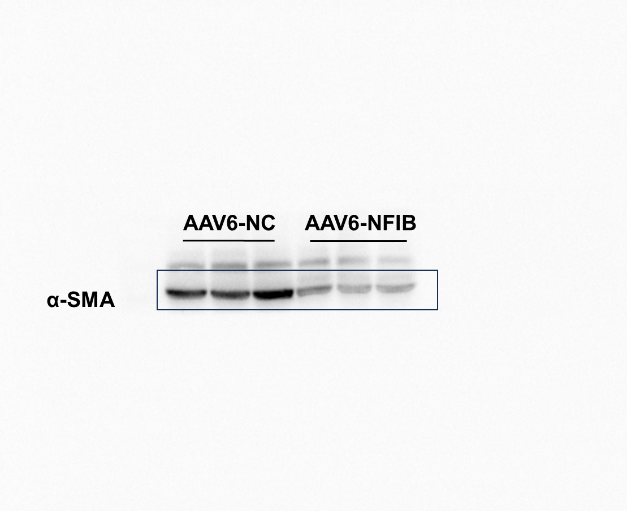

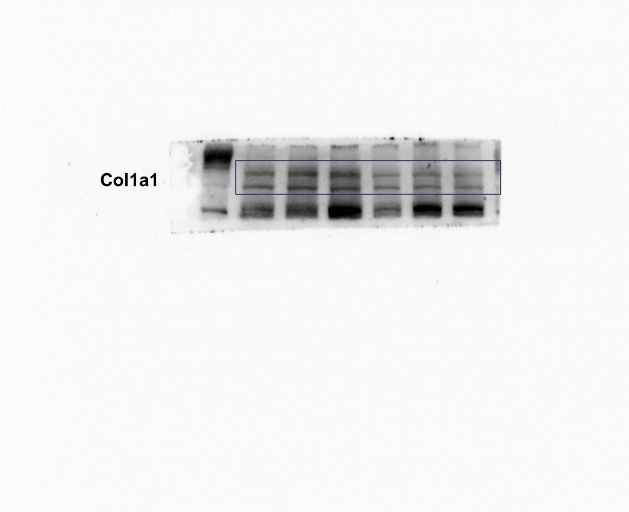


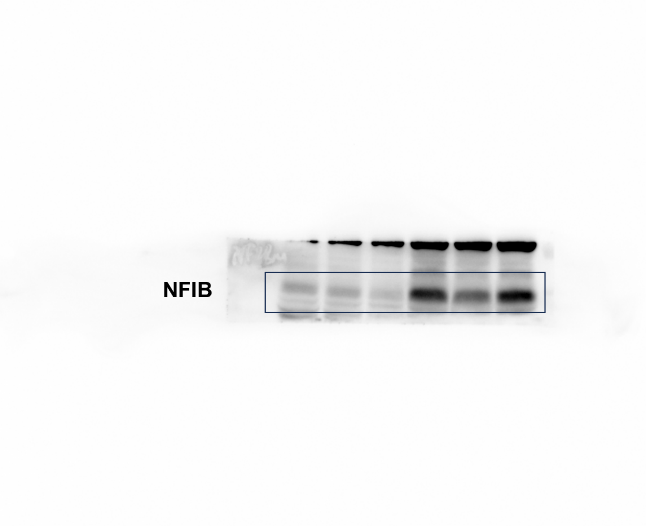

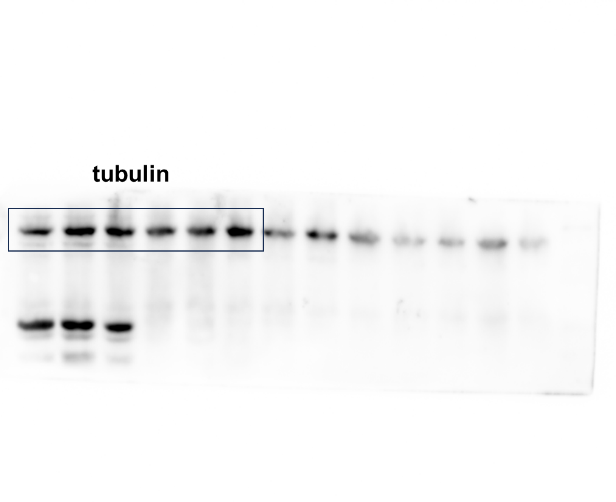


Figure···4


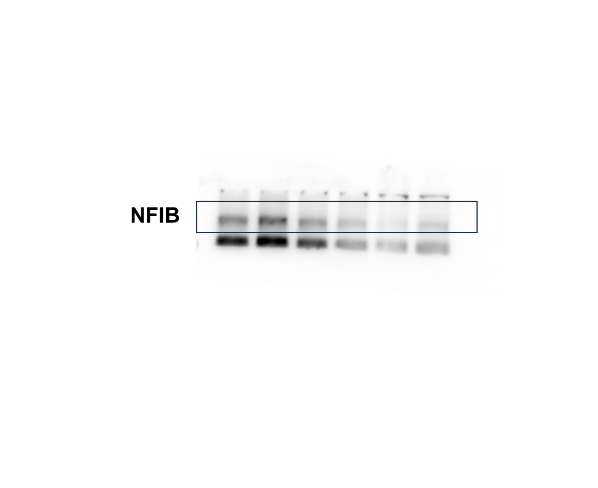

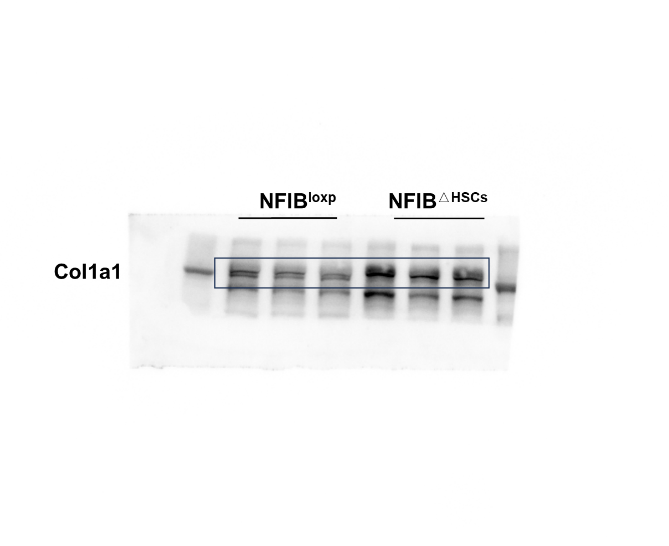


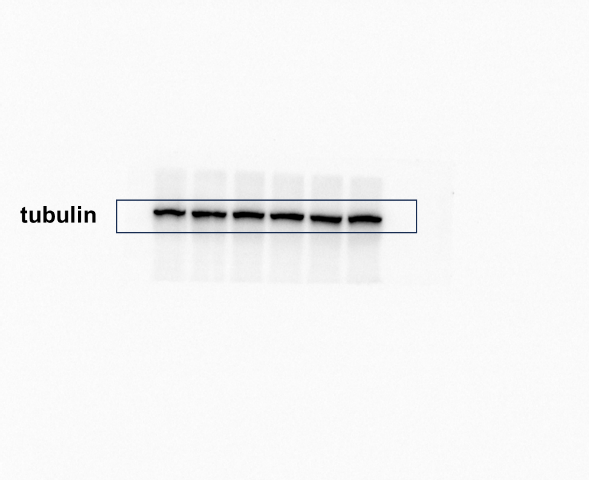


Figure···5


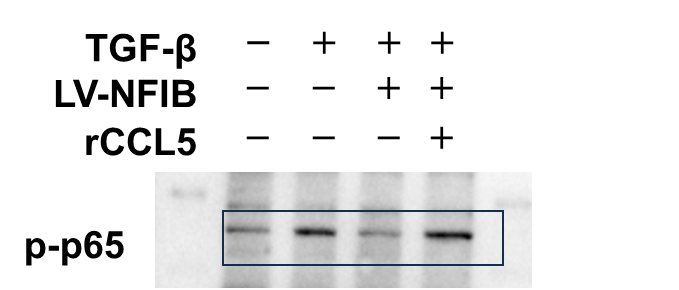

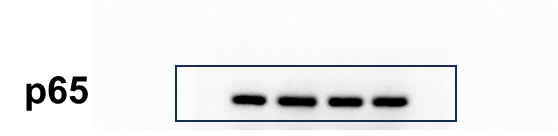


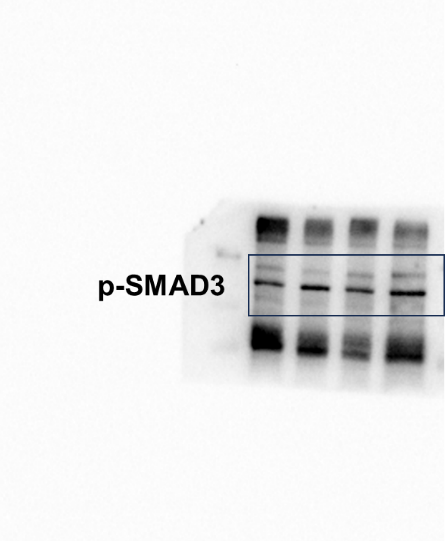

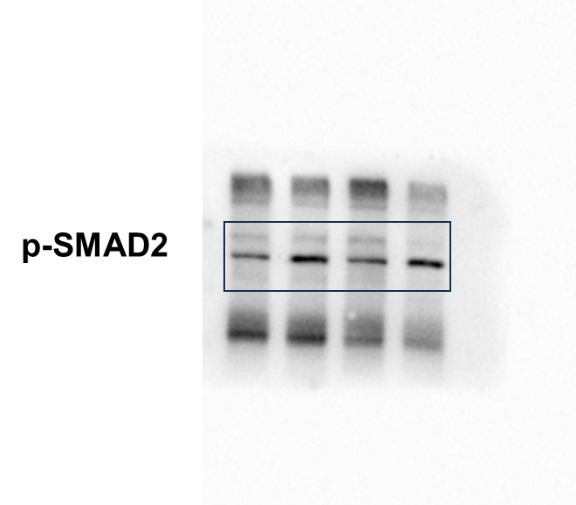


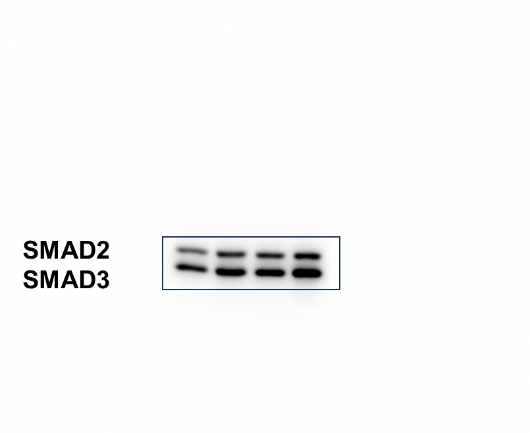

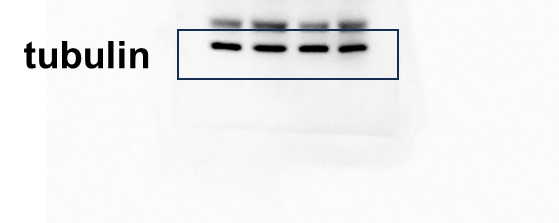


Figure···7


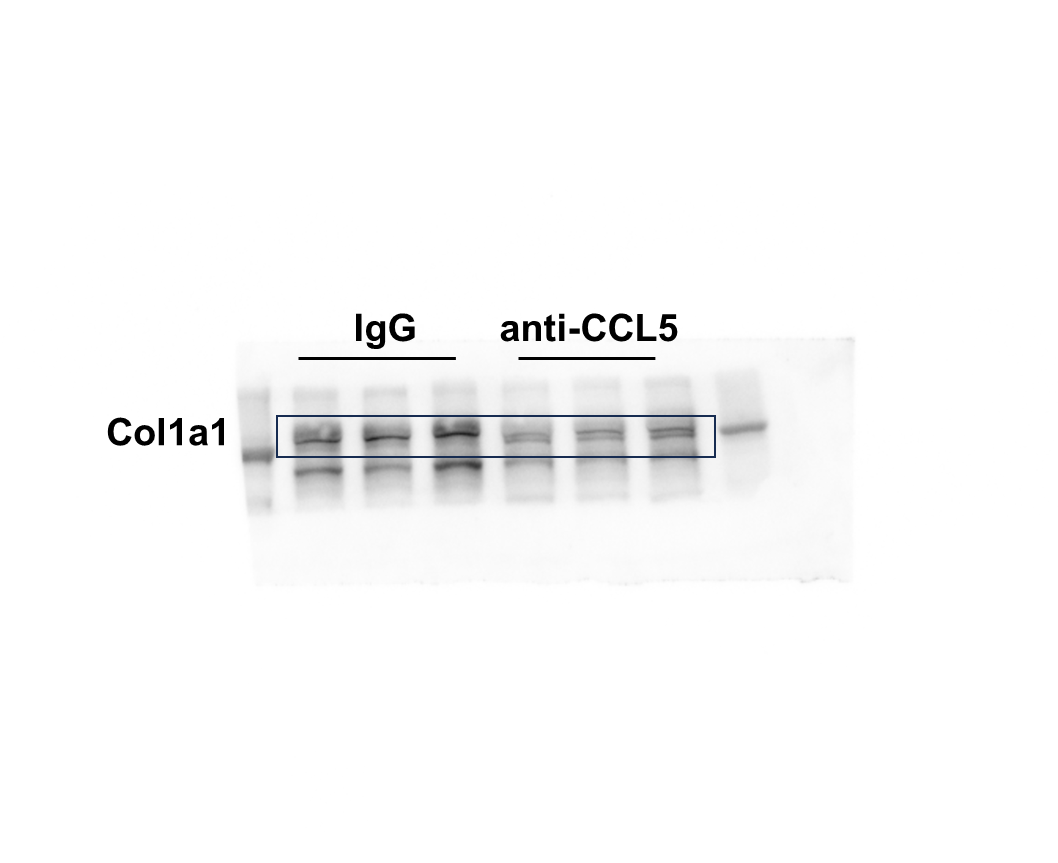

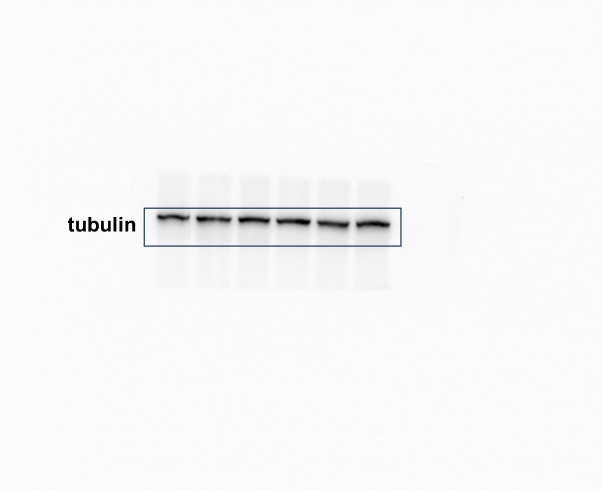


Figure···8


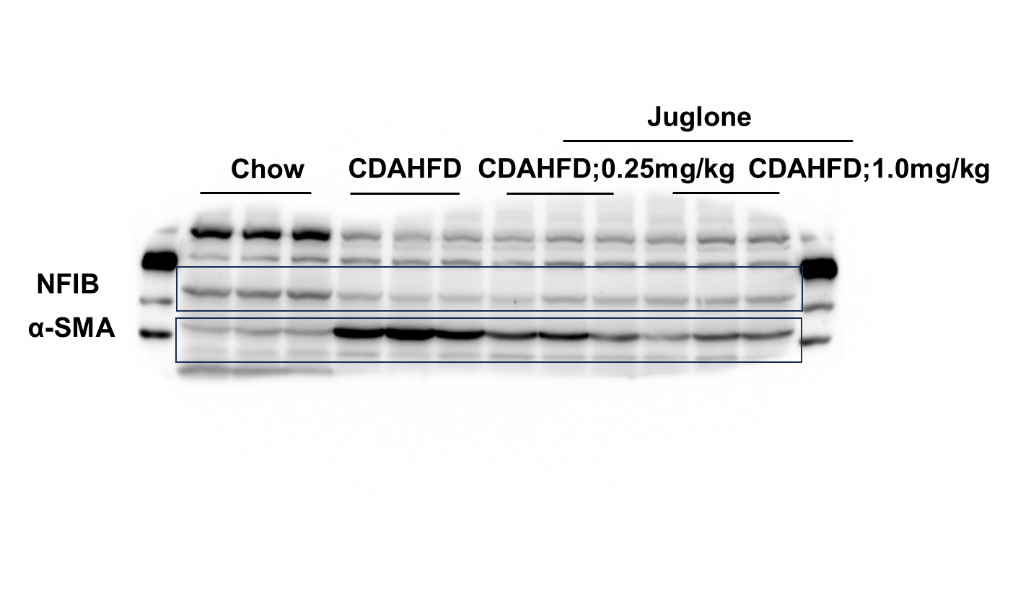


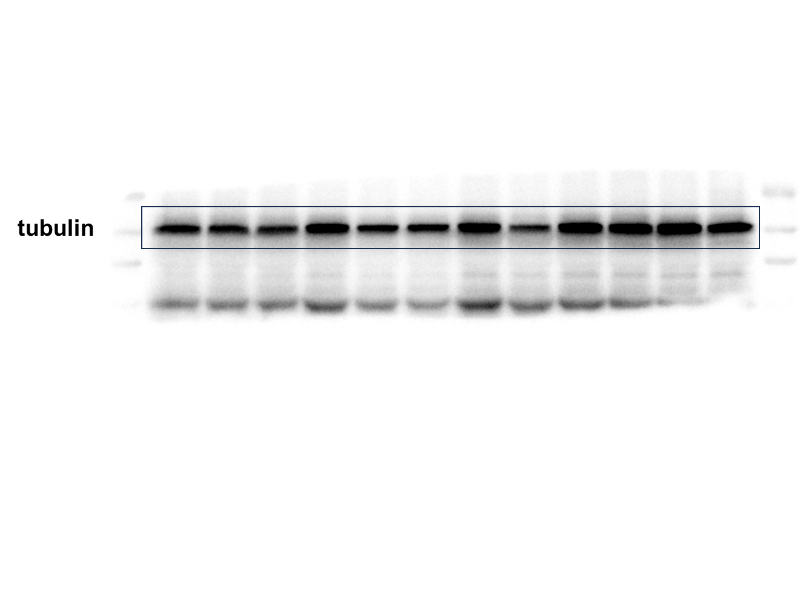


Figure···S4


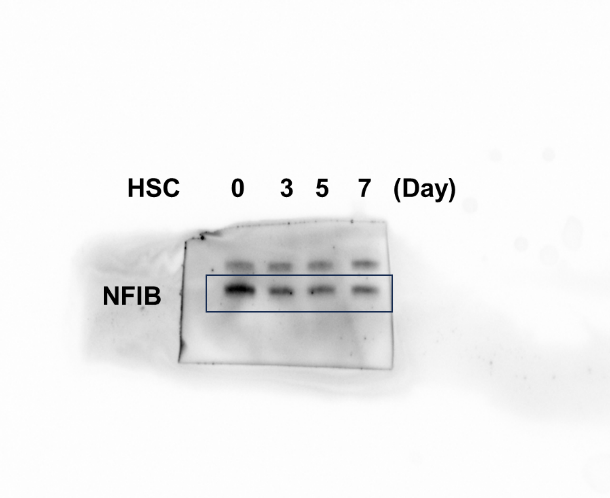

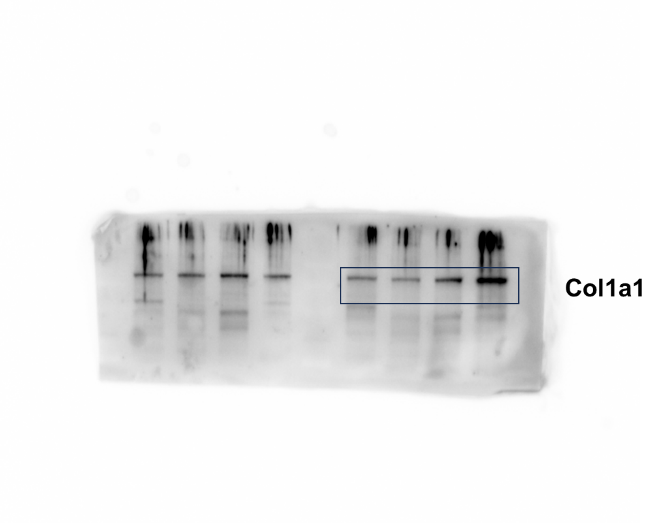


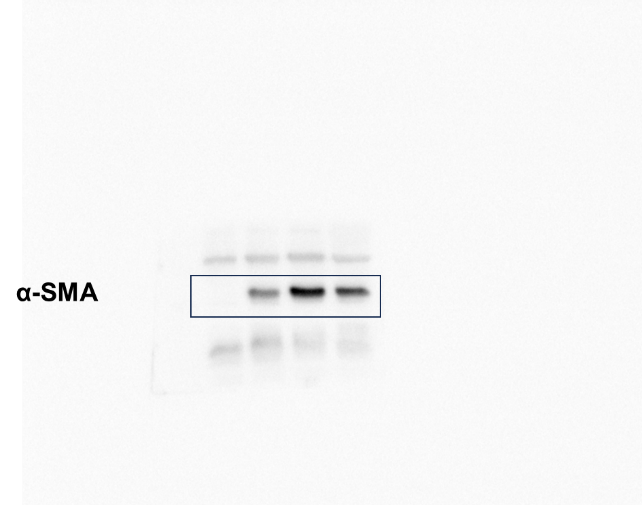

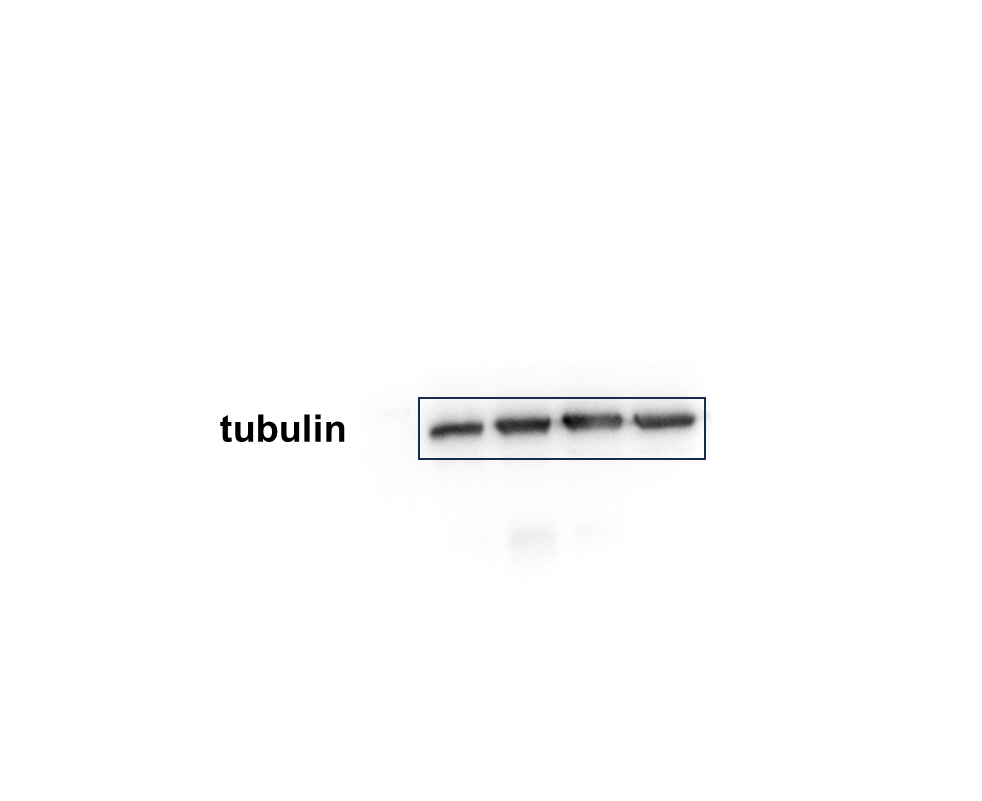


Figure···S5


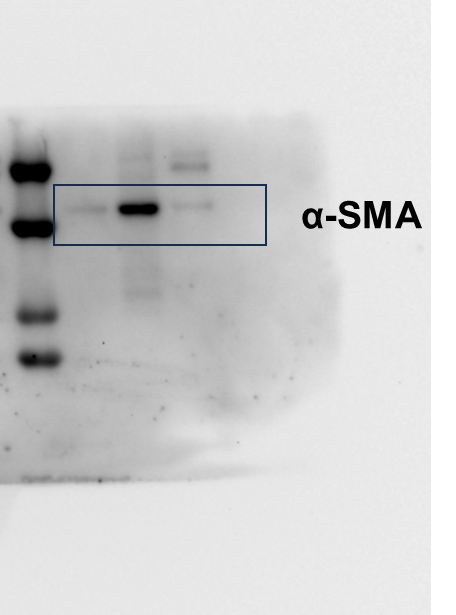

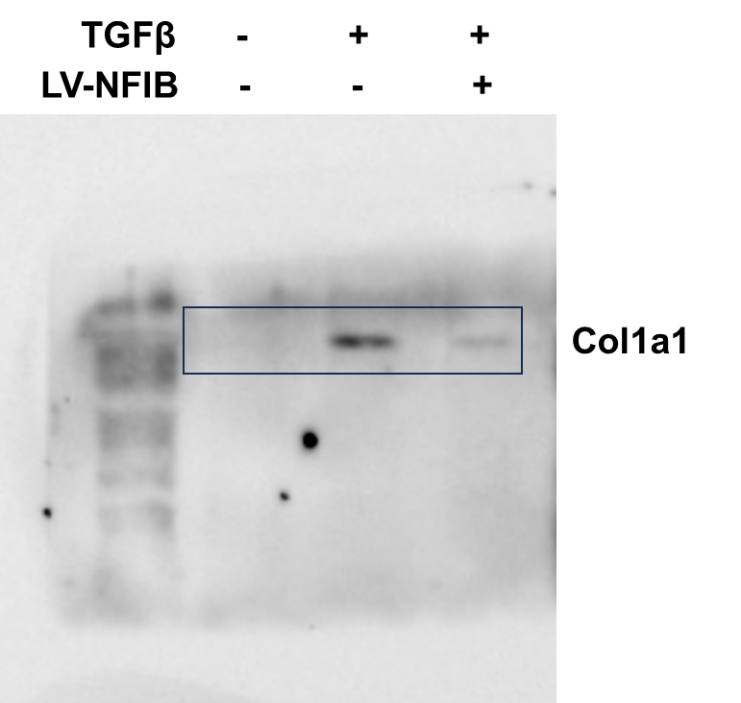


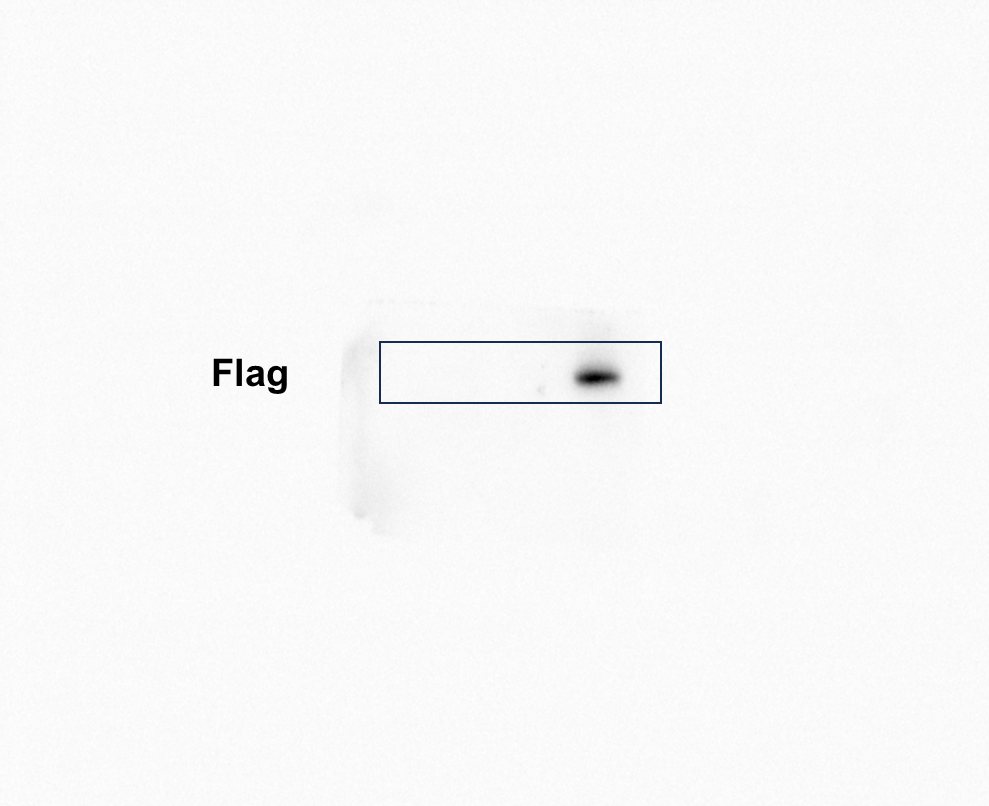

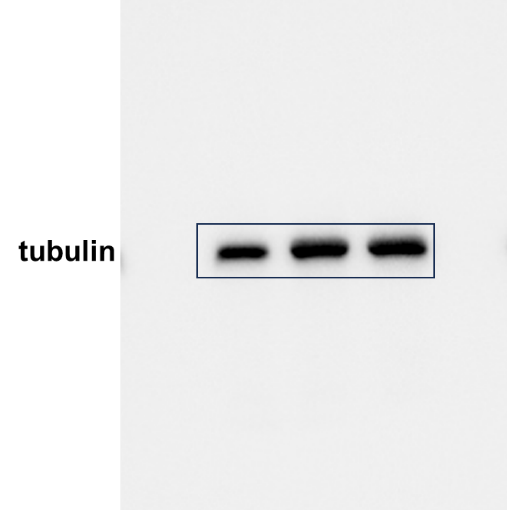


Figure···S8


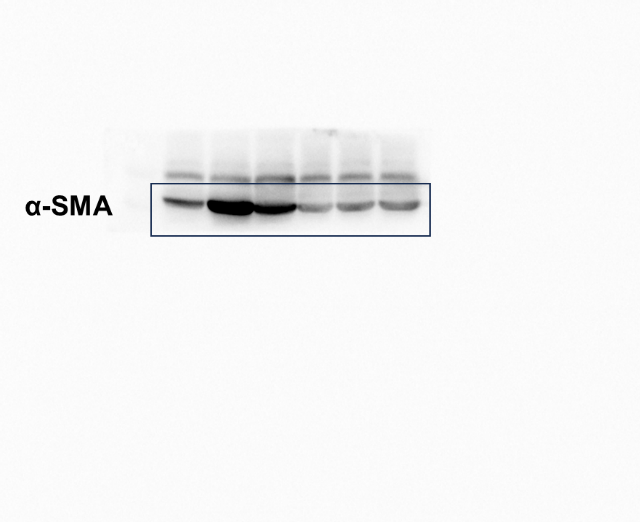

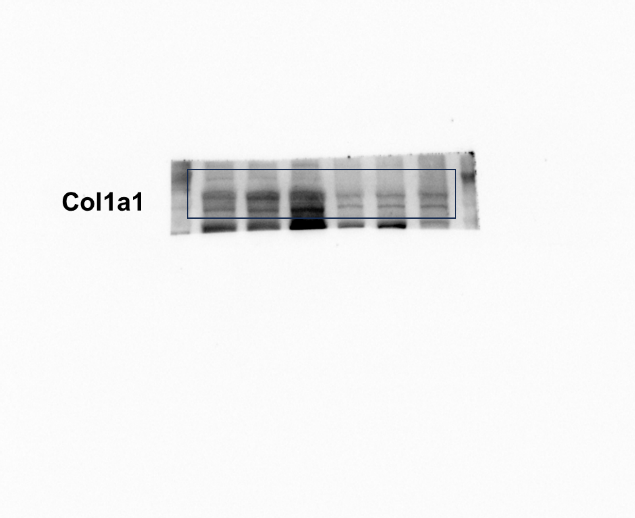


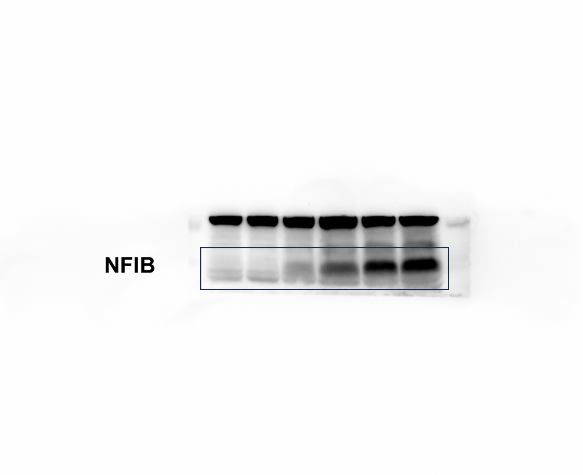

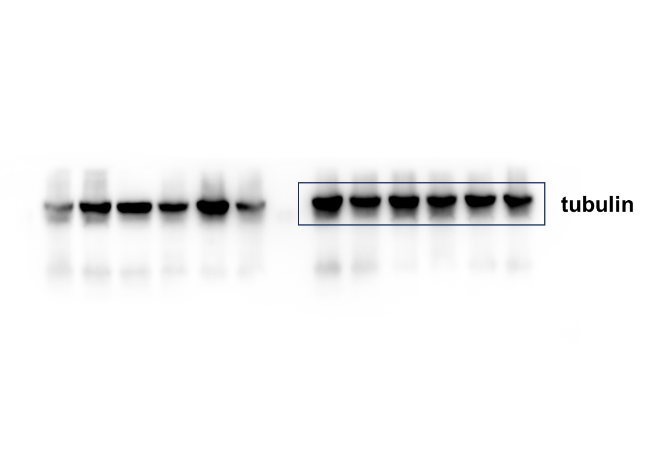


Figure···S9


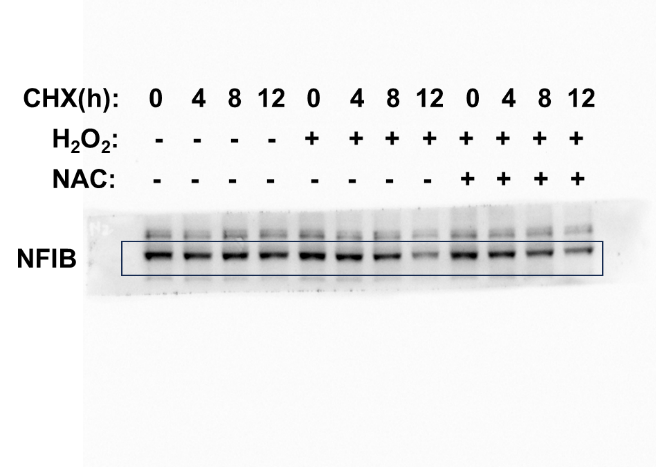

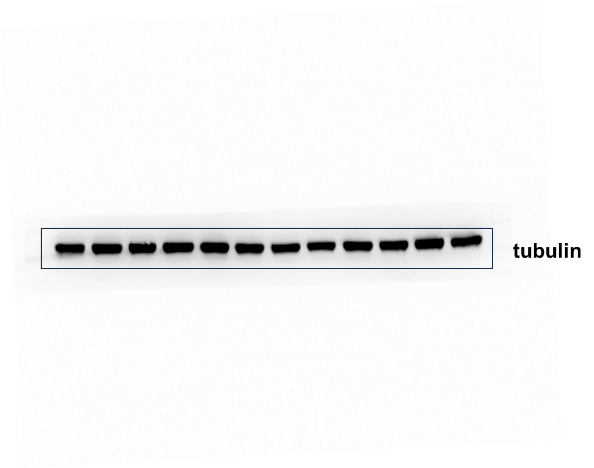


Figure···S13


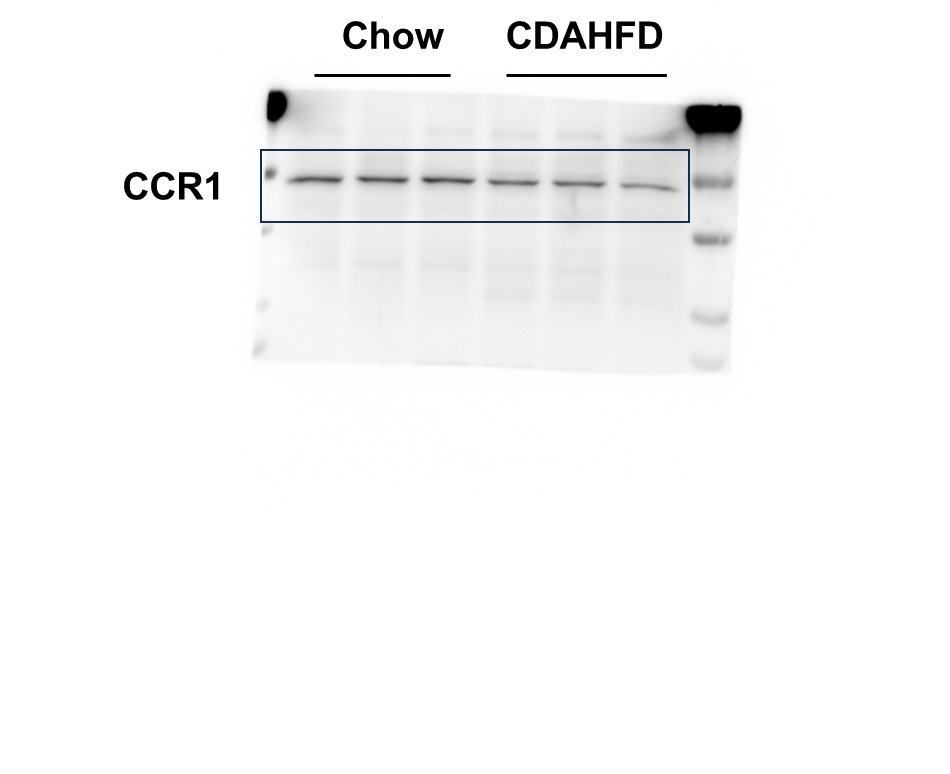

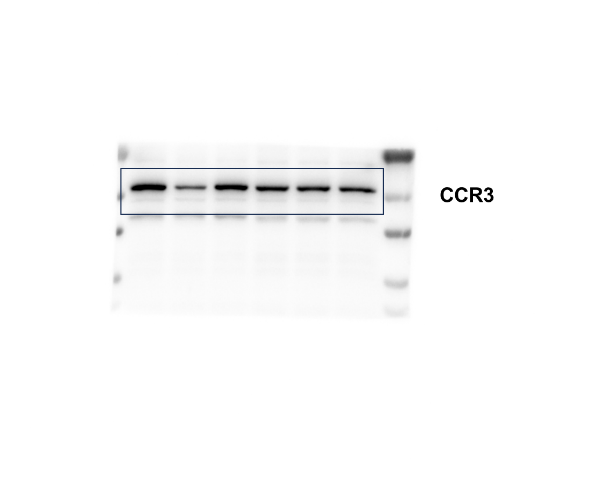


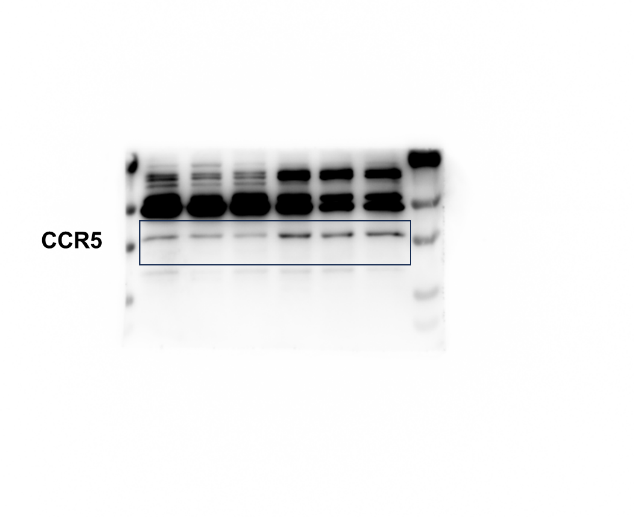

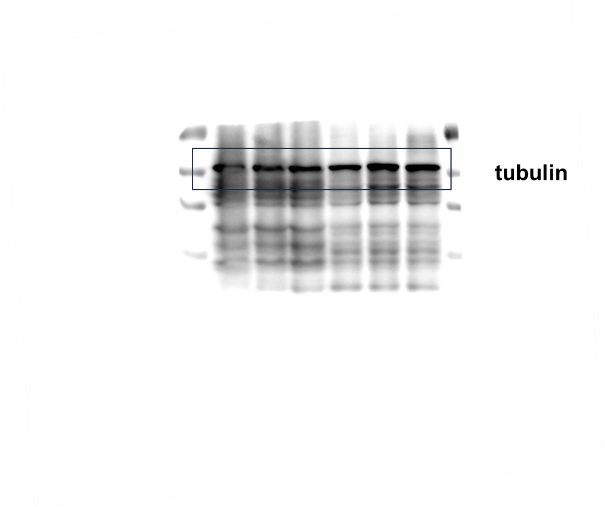


Figure···S14


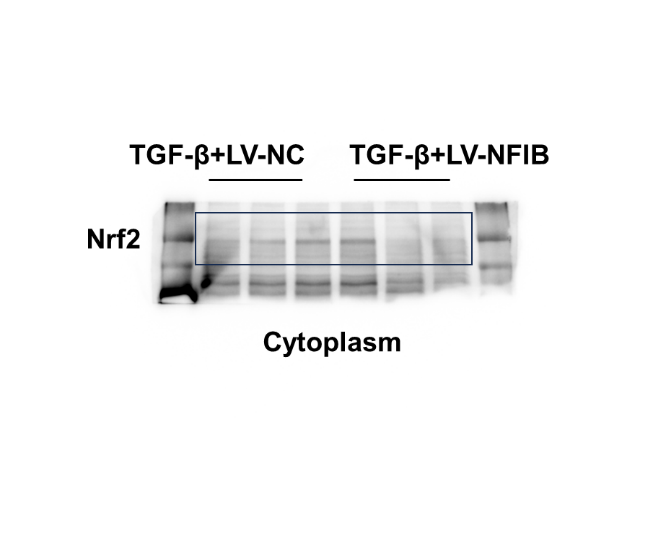

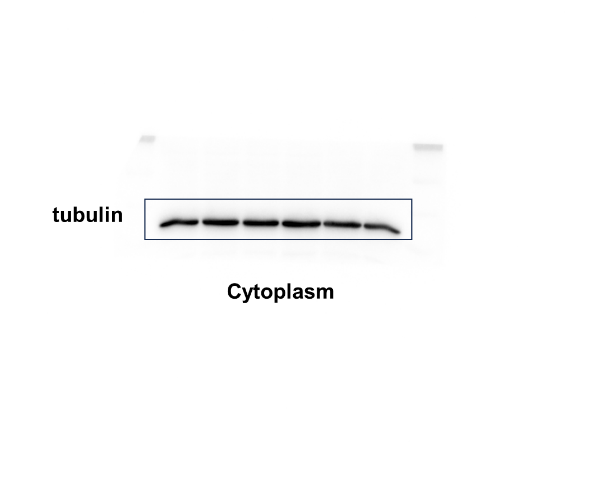


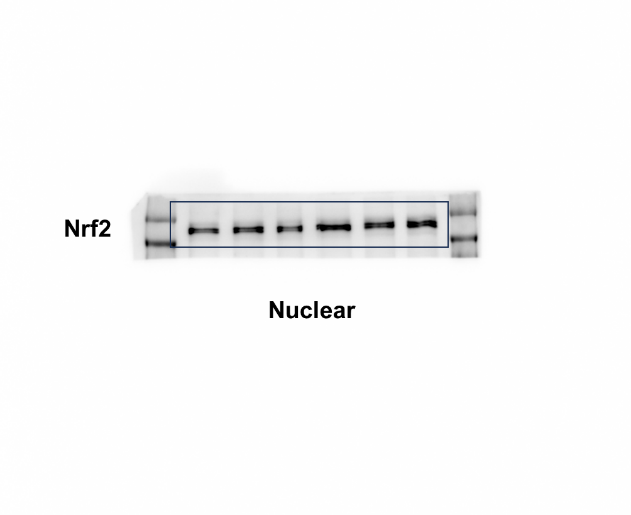

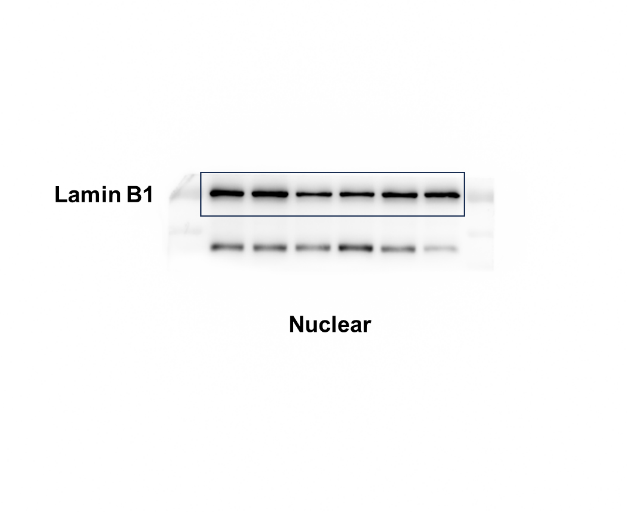

Supplement: Supplementary file 2 — Supporting File 2: advs73472‐sup‐0002‐DataFile.docx. [file ADVS-13-e11311-s001.docx]
